# Chemoenzymatic Dynamic Kinetic Asymmetric Transformations of β‐Hydroxyketones

**DOI:** 10.1002/chem.202102683

**Published:** 2021-10-05

**Authors:** Simon Hilker, Daniels Posevins, C. Rikard Unelius, Jan‐E. Bäckvall

**Affiliations:** ^1^ Department of Organic Chemistry Arrhenius Laboratory Stockholm University 10691 Stockholm Sweden; ^2^ Department of Chemistry and Biomedical Science Linnaeus University 39231 Kalmar Sweden

**Keywords:** β-hydroxyketones, DYKAT, lipase, racemization, ruthenium

## Abstract

Herein we report on the development and application of chemoenzymatic Dynamic Kinetic Asymmetric Transformation (DYKAT) of α‐substituted β‐hydroxyketones (β‐HKs), using *Candida antartica* lipase B (CALB) as transesterification catalyst and a ruthenium complex as epimerization catalyst. An operationally simple protocol allows for an efficient preparation of highly enantiomerically enriched α‐substituted β‐oxoacetates. The products were obtained in yields up to 95 % with good diastereomeric ratios.

## Graphical abstract

**Ruthenium and enzyme catalysis** were combined in a one‐pot procedure to achieve efficient DYKAT of α‐substituted β‐hydroxyketones. This newly developed protocol offers straightforward access to enantiomerically pure and diastereomerically enriched β‐oxoacetates in high yields by employing a ruthenium‐based racemization catalyst for efficient racemization‐epimerization of substrates via formation of non‐chiral 1,3‐diketone intermediates.

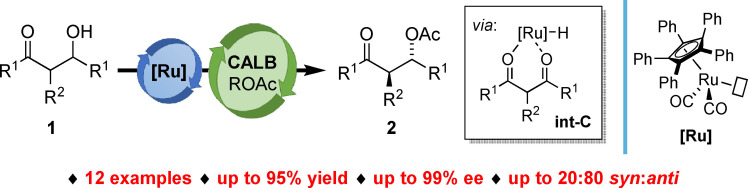

Asymmetric synthesis remains an important part of organic chemistry, strongly impacting other scientific areas.^[1]^ Various areas of chemical industry have a stable growing demand of optically pure compounds,^[2]^ with the resolution of racemic mixtures still being the preferred method industrially.^[3]^ Ever since the possibility to combine enzymes and transition metals in one‐pot procedures was reported,^[4]^ considerable efforts into combining enzymes and transition metals in catalytic systems have been undertaken.^[5]^ Development of systems combining in situ transition metal‐catalyzed racemization with enzymatic kinetic resolution (KR) has resulted in so‐called dynamic kinetic resolution (DKR), efficiently resolving racemic mixtures of e. g. *sec*‐alcohols in theoretically quantitative yields,^[6]^ providing convenient access to valuable functionalized alcohols.^[7]^ Further, chemoenzymatic DKR procedures have been successfully applied in the resolution of α‐hydroxyketones or the asymmetric syntheses of diaryl diols.^[8]^ Recently, also systems using organocatalysis or photocatalysis cooperatively with enzyme catalysis or bi‐enzymatical DKR systems have been developed and applied.^[9]^ Chemoenzymatic DYKAT^[10]^ protocols have been developed for the diastereo‐ and enantioselective transformations of diastereomeric mixtures of diols^[11]^ and found application in the synthesis of enantiomerically pure (+)‐solenopsin A.^[11c]^ We have previously developed a DYKAT of 1,3‐diols to access enantiomerically pure *syn*‐1,3‐diacetates combining enzymatic resolution and Ru‐catalyzed epimerization additionally including intramolecular acyl migration in 1,3‐*syn*‐diol monoacetates (Scheme 1a).^[11a]^ Another example includes preparation of γ‐hydroxyketones from 1,4‐diols that takes advantage of a facile dehydrogenation step when employing Ru‐complex **Ia** together with an acyl donor affording γ‐oxoacetates as products (Scheme 1b).^[11b]^

**Scheme 1:**
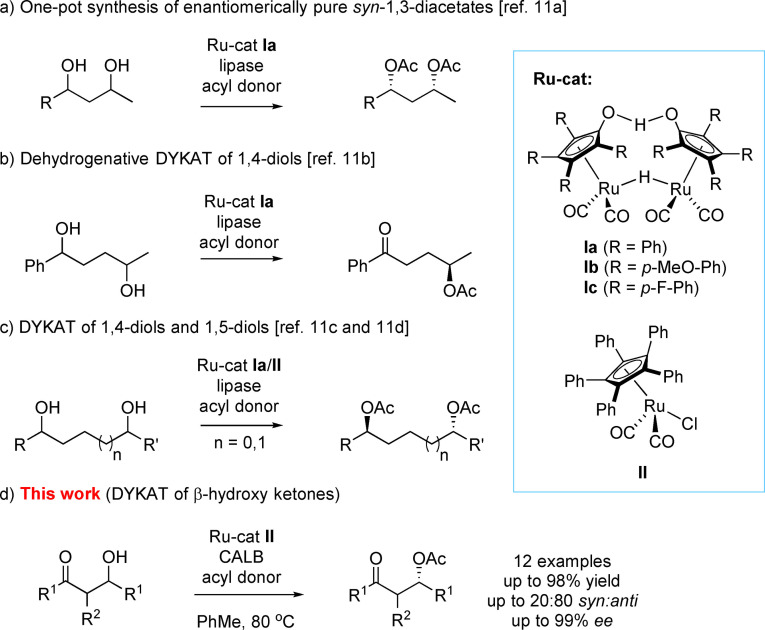
Examples of previously reported methods for DYKAT of diols and this work.

To date various metal‐based racemization catalysts have been reported to be compatible with enzyme catalysis, including ruthenium‐based complexes. Early DKR systems employed a combination of lipase CALB and Shvo's catalyst (**Ia**).^[12]^ Park and co‐workers later introduced a new type of RuCl‐complexes with superior racemization properties.^[13]^ The Bäckvall group developed a highly potent RuCl‐complex (**II**), which has since then found application in various DKR and DYKAT systems.[^6b^, ^6c^, ^6d^, ^14^] With the latter catalyst racemization of 1‐phenylethanol takes place at room temperature in minutes.[^14a^, ^14b^] Further, this catalyst system suppresses the commonly occurring side reaction of substrate oxidation, which is a common problem with the early DKR systems employing catalyst **Ia**.^[6a]^

Chiral β‐hydroxyketones (β‐HKs) are commonly found in nature,^[15]^ for example as pheromone components of Sitona and Sitophilus weevils.[^15e^, ^15f^, ^15g^] In addition, β‐HKs constitute a class of valuable building blocks, commonly employed in the total synthesis of natural products, i. e. polyketides.^[16]^ We envisioned an effective epimerization mechanism for α‐substituted β‐hydroxyketones **1** with RuCl‐complex **II** (Scheme 2a), including a [1,5]‐migration of ruthenium hydride species ([Ru]‐H) between the oxygen atoms of the 1,3‐diketone moiety in intermediates **int‐A** and **int‐B** as the key step (Scheme 2b). The transformation is proposed to proceed via a non‐chiral intermediate **int‐C**. It has been previously demonstrated during mechanistic studies on the racemization of *sec*‐alcohols with RuCl‐complex **II** that the substrate does not leave the coordination sphere of the metal during the oxidation‐reduction process.^[17]^ In contrast, the use of Ru‐complex **I** under analogous reaction conditions would lead to an equilibrium where the 1,3‐diketone would readily dissociate from the corresponding [Ru]‐H moiety, which would in turn lead to oxidation of the substrate.^[18]^ Furthermore, an expected reaction rate difference between the enzymatic acylation of *syn*‐ and *anti‐* diastereoisomers of α‐substituted β‐HKs would lead to formation of highly useful diastereomerically enriched β‐oxoacetates as products.

**Scheme 2:**
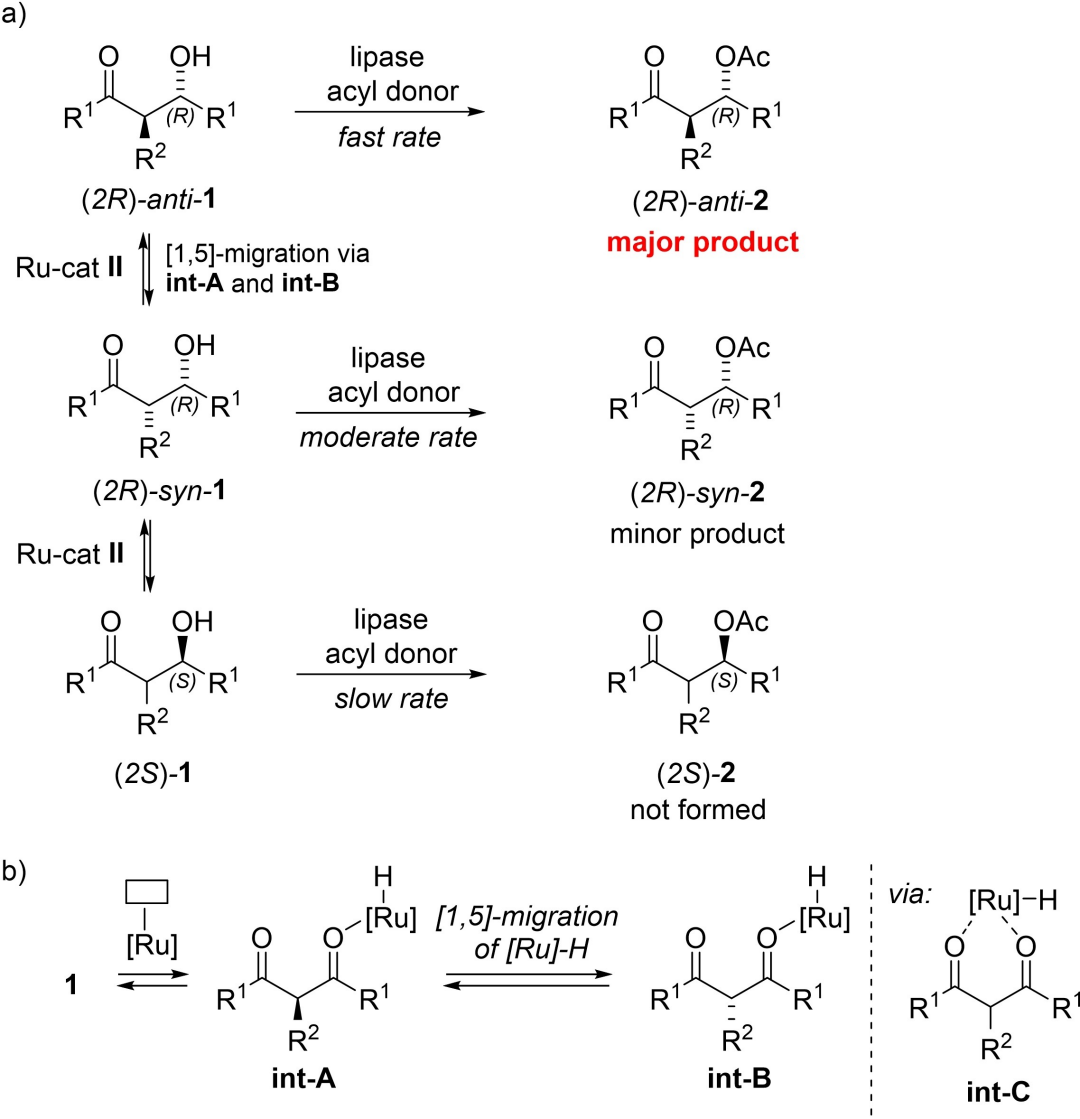
a) Racemization pathways in the DYKAT of β‐HKs employing Ru‐cat **II**. b) [1,5]‐Migration of [Ru]‐H as the key step in the racemization process.

We postulated that an increase of the steric demand of the substituent in the α‐position of the β‐HKs would lead to improved diastereoselectivity of the overall process. Initial attempts to obtain a DYKAT of β‐HKs indicated a significant drop in the rate of the enzymatic acylation when α‐substituted 3‐hydroxy‐5‐heptanones were used as substrates compared to that of 2‐hydroxy‐4‐pentanones. Hence, β‐HK **1 a** bearing a benzyl substituent in the α‐position was chosen as the standard substrate in the optimization of the reaction conditions (Table 1).

**Table 1:** Optimization of the reaction conditions.^[a]^

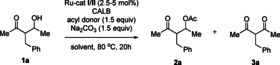							
Entry	Ru‐cat [mol %]	CALB [mg/mmol]	solvent [M]	**2 a** [%]^[b]^	**3 a** [%]^[b]^	*syn‐* **2 a** : *anti‐* **2 a** ^[c]^	*ee* of *anti‐* **2 a** [%]^[c]^
1	**Ia** (2.5)	40	CyH (0.2)	49	8	30 : 70	97
2	**Ia** (2.5)	80	CyH (0.2)	83	8	30 : 70	97
3	**Ia** (2.5)	80	THF (0.2)	39	–	32 : 68	99
4	**Ia** (2.5)	80	DCE (0.2)	27	–	30 : 70	99
5	**Ia** (2.5)	80	*t*BuOH (0.2)	12	8	23 : 77	99
6	**Ia** (2.5)	80	PhMe (0.2)	85	10	30 : 70	97
7	**Ib** (2.5)	80	PhMe (0.2)	81	5	34 : 66	95
8	**Ic** (2.5)	80	PhMe (0.2)	78	5	38 : 62	95
9	**II** (5.0)^[d]^	40	PhMe (0.2)	88	–	30 : 70	99
10^[e]^	**II** (5.0)^[d]^	40	PhMe (0.2)	95 (90)^[f]^	–	35 : 65	99
11^[e,g]^	**II** (5.0)^[d]^	‐	PhMe (0.2)	88	–	48 : 52	96

[a] Unless otherwise noted reactions were conducted under argon atmosphere in the indicated solvent (1.0 mL) at 80 °C using **1 a** (0.2 mmol), *p*‐ClPhOAc (1.5 equiv), Na_2_CO_3_ (1.5 equiv), Ru‐cat (2.5–5 mol%), and CALB (indicated amount). [b] Yield determined by ^1^H NMR using mesitylene as the internal standard. [c] *dr* and *ee* determined by GC on chiral stationary phase. [d] Using KO*t*Bu (0.1 M solution in toluene, 5 mol%) as an additive. [e] Using isopropenyl acetate (1.5 equiv) as the acyl donor. [f] Isolated yield. [g] Using lipase PS‐IM (80 mg/mmol) instead of CALB and the reaction time was 64 h. DCE=1,2‐dichloroethane.

Initially experiments were conducted employing Ru‐complex **Ia** as the racemization catalyst. *p*‐Chlorophenyl acetate was chosen as the acyl donor due to the observed increased formation of the undesired diketone **3 a** when acyl donors such as vinyl acetates were employed. An enzyme loading of 80 mg/mmol of lipase CALB was found to be necessary for achieving good yield of the desired product **2 a** in cyclohexane as the solvent after 20 h at 80 °C. The desired β‐oxoacetate **2 a** was formed in 83 % yield, with a moderate diastereomeric ratio (*dr*) with a *syn*:*anti* ratio of 30 : 70 (Table 1, entry 2). High enantiomeric excess (*ee*) was observed, even though the formation of minor amounts of the undesired enantiomer suggests that epimerization is not sufficiently fast over the whole reaction course. Substrate oxidation to give **3 a** as a byproduct in small amounts occurred as a result of acceptorless dehydrogenation of **1 a** catalyzed by Ru‐complex **Ia**.^[12]^ Use of solvents such as THF, DCE or *t*BuOH in the reaction led to a considerable decrease of the yield of **2 a** (Table 1, entries 3–5). Toluene was found to be the best solvent in this transformation (Table 1, entry 6). Screening of other racemization catalysts such as Ru‐catalysts **Ib** and **Ic** led to decreased yields of **2 a**, as well as lower *ee* values (Table 1, entries 7–8).

To further improve the enantioselectivity of the reaction and suppress formation of the undesired oxidation product **3 a**, RuCl‐complex **II** was tested as a racemization catalyst in this reaction (Table 1, entry 9). Due to the superior epimerization performance of catalyst **II**, the enzyme loading could be reduced to half while retaining high yield and *ee* of **2 a**. The RuCl‐complex **II** was also found to be compatible with isopropenyl acetate as the acyl donor (Table 1, entry 10), which facilitated the product isolation. Under these optimized reaction conditions β‐oxoacetate **2 a** was obtained in 90 % isolated yield, 99 % *ee* and a *syn* : *anti* ratio of 35 : 65 with no detected formation of **3 a**. We also tested the performance of lipase PS‐IM in the DYKAT of **1 a** (Table 1, entry 11). Under analogous reaction conditions after 64 h reaction time the β‐oxoacetate **2 a** was obtained in 88 % yield, albeit with a decrease of both *dr* and *ee*.

To gain further insight into the diastereoselectivity of the enzymatic acylation reaction of **1 a**, the relative rates of the formation of diastereomers *syn*‐**1 a** and *anti*‐**1 a** were measured. First, a parallel experiment was carried out using *syn*‐**1 a** and *anti*‐**1 a** as substrates (Figure 1). In this setting, alcohol *anti‐* **1 a** undergoes acetylation to furnish *anti*‐**2 a** approximately twice as fast as *syn*‐**1 a**.

**Figure 1:**
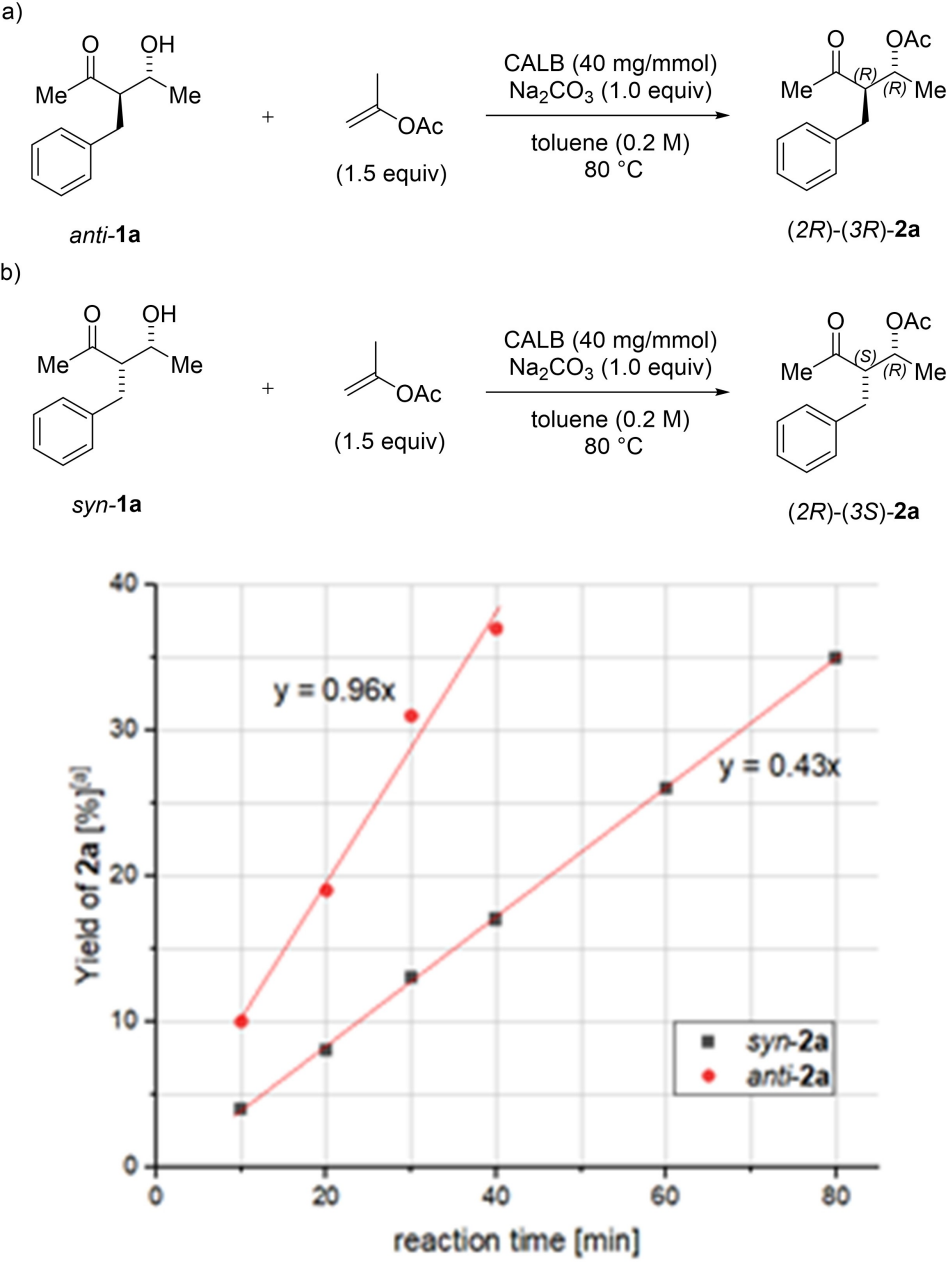
Kinetic resolution (KR) of a) *syn‐* **1 a** and b) *anti‐* **1 a** (parallel experiments). [a] Yield determined by ^1^H NMR using mesitylene as the internal standard.

Additionally, a competitive KR experiment was run, using compound **1 a** with a starting diastereomeric ratio close to 1 : 1 *syn* : *anti* (Scheme 3). The faster reacting diastereomer preferentially binds to the enzyme and thereby prevents access of the slower reacting diastereomer, potentially amplifying the diastereoselection. The competitive reaction indicates a relative rate difference of 1 : 3 between *syn‐* and *anti‐*diastereoisomers, based on the observed *dr* of the product **2 a** at low conversion.

**Scheme 3:**
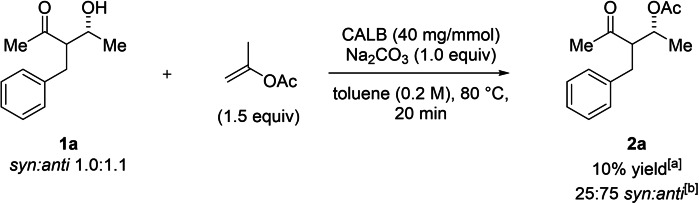
Kinetic resolution (KR) of *syn‐* **1 a** and *anti‐* **1 a** (intermolecular competition experiment). [a] Yield determined by ^1^H NMR using mesitylene as the internal standard. [b] *dr* and *ee* determined by GC on chiral stationary phase.

Next, the scope of the newly developed DYKAT reaction was investigated (Scheme 4). Under the previously established optimal reaction conditions (Conditions A), products **2 a**–**c** were obtained in excellent yields, very high enantioselectivity, and moderate *dr*. However, β‐HK **1 d** with *n*‐butyl substituent in the α‐position led to *dr* of 45 : 55 (*syn* : *anti*) under these reaction conditions. We argued that the rate of enzymatic acylation of **1 d** is too fast for an efficient epimerization to occur by [1,5]‐migration of RuH. Hence, we further investigated if an increased diastereomeric ratio of **2 d** could be achieved by lowering the enzyme‐to‐catalyst ratio. After an additional screening of reaction conditions (see the Supporting Information) new optimized reaction conditions were established (Conditions B). We observed that decreasing the amount of enzyme in the reaction decreased the yield and increased the reaction time, whereas raising the amount of rutheniumcatalyst to 7.5 mol% and diluting the reaction mixture resulted in high yield of product **2 d** with increased *dr* compared to the reaction with the previously used optimized conditions (Conditions A). The newly optimized reaction conditions (Conditions B) were further applied for substrates **1 b**, **1 c**, and **1 e** in order to achieve higher *dr* of the corresponding products. Substrate **1 f** containing a terminal alkyne moiety afforded the corresponding β‐oxoacetate **2 f** in 45 % yield with moderate *dr*, but with decreased *ee* of both diastereoisomers. The yields of acetates **2 g** and **2 h** obtained were low due to their instability under the reaction conditions (they readily underwent elimination reactions to form the corresponding α,β‐unsaturated ketones as side products). Higher enzyme loading and longer reaction times were necessary to achieve efficient DYKAT of the 3‐hydroxy‐5‐heptanone‐derived β‐HKs **1 i**–**k** in good yields. Surprisingly, α‐methyl‐substituted β‐oxoacetate **2 i** showed a slight preference for the *syn*‐diastereomer, in contrast to the previous examples described here. A plausible reason for this preference is the way that the β‐HK adapts to the enzyme pocket, since the methyl substituent is less sterically demanding than the propionyl moiety in **2 i**. Introducing additional steric hindrance by creating a quaternary stereogenic center in β‐HK **1 l** caused an increased steric demand. Even after prolonged reaction time, β‐oxoacetate **2 l** was obtained in low yield, with just marginally better *dr* than **2 a** and decreased *ee*. Interestingly, cyclic β‐HK **1 m** was also found to be compatible with the newly developed DYKAT protocol and afforded the corresponding β‐oxoacetate **2 m** in 80 % yield and *dr* of 40 : 60 (*syn* : *anti*).

**Scheme 4:**
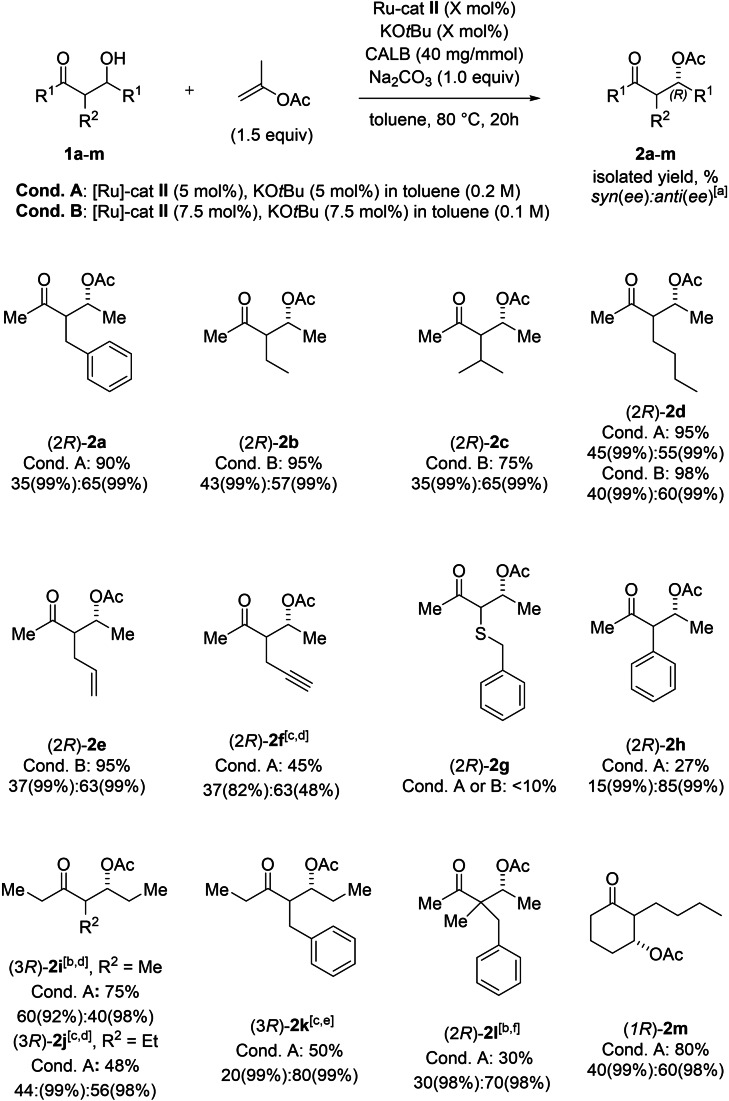
Scope of DYKAT of β‐HKs. Unless otherwise noted the reaction was conducted under argon atmosphere in anhydrous toluene (indicated amount) at 80 °C using **1** (0.2 mmol), Na_2_CO_3_ (1.0 equiv), Ru‐cat **II** (indicated amount), KO*t*Bu (0.1 M solution in toluene, indicated amount) and CALB (40 mg/mmol). [a] *dr* and *ee* determined by GC on chiral stationary phase. [b] 80 mg/mmol of CALB was used. [c] 120 mg/mmol CALB. [d] Reaction time was 48 h. [e] Reaction time was 70 h. [f] Reaction time was 90 h.

As it was previously demonstrated, Ru‐complex **Ia** can be used to efficiently reduce ketones to alcohols via transfer hydrogenation by the use of an external alcohol as a hydrogen donor.^[19]^ Herein we disclose a tandem hydrogenation‐DYKAT of 1,3‐diketone **3 a** as a one‐pot procedure (Scheme 5). By employing Ru‐complex **Ia** as the racemization/transfer hydrogenation catalyst, the mono reduction of the 1,3‐diketone moiety in **3 a** and the subsequent epimerization and enzymatic acylation of the in situ generated β‐HK, afforded the desired β‐oxoacetate **2 a** in 65 % NMR‐yield with high enantiomeric excess.

**Scheme 5:**
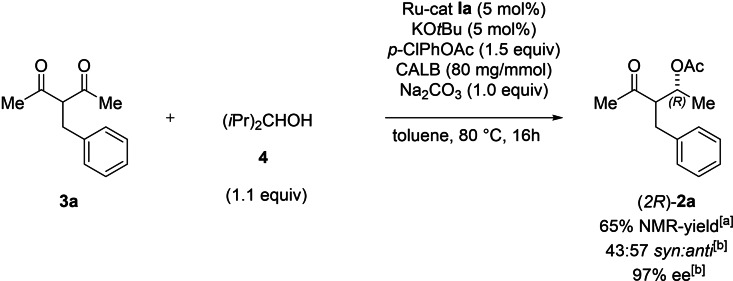
Tandem hydrogenation‐DYKAT of 1,3‐diketone **3 a**. The reaction was conducted under argon atmosphere in anhydrous toluene (1 mL) at 80 °C using **3 a** (0.2 mmol), Na_2_CO_3_ (1.0 equiv), Ru‐cat **Ia** (5 mol%), *p*‐ClPhOAc (1.5 equiv), alcohol **4** (1.1 equiv) and CALB (80 mg/mmol). [a] Yield determined by ^1^H NMR using 1,3,5‐trimethoxybenzene as the internal standard. [b] *dr* and *ee* determined by GC on chiral stationary phase.

In conclusion, we have reported the first protocol for chemoenzymatic DYKAT of α‐substituted β‐HKs. The newly developed method afforded highly useful β‐oxoacetates as products in good yields with high enantioselectivity and moderate diastereoselectivity. The diastereoselectivity of the overall process is proposed to be dependent on the rate difference of the enzymatic acylation of *syn‐* and *anti‐*diastereomers of the β‐HK which is largely influenced by the steric demand of the substituent in the α‐position. While lipase CALB performed well and afforded moderate to good *dr* of the target β‐oxoacetates, the use of lipase PS‐IM led to considerable decrease of the diastereoselectivity of the reaction. We expect, that future improvements in terms of diastereoselectivity can be achieved by the discovery of even more selective lipase enzymes in the near future. A complimentary approach would be to use genetic tools like directed evolution where the enzyme performance could be specifically tailored to the described DYKAT protocol.

## Conflict of interest

The authors declare no conflict of interest.

## Supporting information

As a service to our authors and readers, this journal provides supporting information supplied by the authors. Such materials are peer reviewed and may be re‐organized for online delivery, but are not copy‐edited or typeset. Technical support issues arising from supporting information (other than missing files) should be addressed to the authors.

Supporting Information
