# Supplementary material for: Chemoenzymatic Dynamic Kinetic Asymmetric Transformations of β‐Hydroxyketones
Source: Chemistry. 2021 Oct 5;27(63):15623–7. doi: 10.1002/chem.202102683 (PMC9293354; doi:10.1002/chem.202102683)
Supplement: Supplementary file 1 — Supporting Information [file CHEM-27-15623-s001.pdf]

Chemistry–A European Journal

Supporting Information

Chemoenzymatic Dynamic Kinetic Asymmetric Transformations of β-Hydroxyketones

Simon Hilker, Daniels Posevins,* C. Rikard Unelius, and Jan-E. Bäckvall*

Contents

General Information S1

Synthesis of β-Hydroxyketones (β-HKs) 1a-1m S2

Optimization of the reaction conditions for formation of 2d* S12

Preparation of β-oxoacetates 2a-2m S13

DYKAT of β-hydroxyketones 1a-1m S14

References S32

^{1}H- and ^{13}C-NMR spectra of compounds S33

General Information

Unless otherwise noted, all reagents were used as received from commercial suppliers. Dry solvents were obtained from commercial sources, from a VAC™ drying system or dried over molecular sieves. All reactions were conducted in dry flasks under argon atmosphere. Room temperature is ca. 22 °C. Reactions were monitored using Merck silica gel 60 F254 plates (TLC analysis). TLC plates were visualized with UV light (254 nm) or $KMnO_4$. Flash column chromatography was carried out with 60Å (particle size 35 - 70 µm) silica gel. ^{1}H-/^{13}C-NMR experiments were performed on a Bruker NMR (400/101 MHz) or (500/125 MHz) at room temperature. Chemical shifts (δ) are reported in parts per million (ppm) relative to the $CDCl_3$ peak (δ(H) = 7.26 and δ(C) = 77.16 ppm). Coupling constants (*J*) are reported in Hertz (Hz). HRMS were recorded on a Bruker MicroTOF spectrometer equipped with an ESI or APCI as ion sources. The enantiomeric excess (*ee*) of compounds was determined by GC on a chiral stationary phase using racemic compounds as references. GC analyses were performed using the following instruments: a) Varian GC 3900 using an IVADEX-1 chiral column from IVA Analysentechnik with FID detector and N_2 as a carrier gas with a flow of 1.8 mL/min; b) Agilent 8860 GC System using an Hydrodex β-DM chiral column with 5977B GC/MSD detector and N_2 as a carrier gas with a flow of 1.8 mL/min.

Candida antarctica lipase B (CALB) was used as immobilized and thermostable Novozyme 435. Lipase Amano was used as commercially available, immobilized and thermostable PS-IM. Na_2CO_3 was dried at 150 °C over night prior to use.

The absolute configurations of the stereogenic centers in products were assigned based on the Kazlauskas rule.

Ruthenium complexes **Ib**, **Ic** and **II** were prepared in accordance with the previously reported procedures.[1]

Synthesis of β-Hydroxyketones (β-HKs) 1a-1m

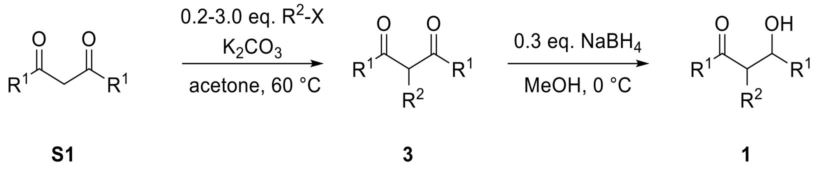

General procedure A **for the preparation of β-hydroxyketones 1a-1f and 1i-1k**

1,3-Diketone **S1** was added (1.0 eq.) to a suspension of K_2CO_3 (1.5 eq.) in acetone (0.3 M). After stirring for 5 min, alkyl halide (0.2 – 3.0 eq.) was added and the resulting reaction mixture was stirred at 60 °C for 16 h to 72 h. The completion of the reaction was determined by TLC. After cooling to room temperature, the reaction mixture was filtered and concentrated in vacuo. Purification over SiO_2 (eluent: *n*-pentane/EtOAc) afforded the corresponding α-substituted 1,3-diketones **3.** Compounds **3** were further used in the next steps without characterisation.

To solution of the 1,3-diketone **3** (1.0 eq.) in MeOH (0.2 M) at 0 °C was added a solution of sodium borohydride (0.3 eq.) in methanol (3 mL). The resulting reaction mixture was stirred at 0 °C for 1-2 h (reaction progress was monitored by TLC). Acetone (25 mL) was added, and the reaction mixture was stirred at room temperature for 15 min. Saturated aqueous ammonium chloride solution (15 mL) was added and the solvent was partially removed under reduced pressure. The resulting mixture was extracted with EtOAc (3 x 50 mL). The combined organic phases were dried over Na_2SO_4, filtered and concentrated in vacuo. The crude products were purified over SiO_2 (eluent: *n*-pentane/EtOAc) to yield the desired β-hydroxyketones **1** as products.

The relative configuration of the α-substituted β-HKs was assigned using NMR spectroscopy. According to previous observations by Kalaitzakis and Heatchcock the ^{1}H-NMR signal of the β-proton of the *syn*-diastereomer is shifted to downfield, whereas ^{13}C-NMR signal of the corresponding β-carbon is shifted upfield compared to the *anti*-diastereomer.[2]

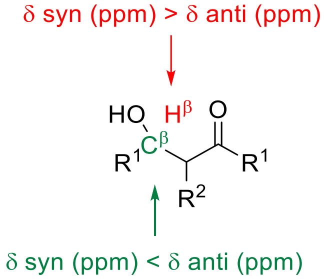

Scheme S1. Observed differences in chemical shift in ^{13}C- and ^{1}H-NMR of β-HK with *syn* and *anti*-configuration.

*3-Benzyl-4-hydroxy-2-pentanone (**1a**)*

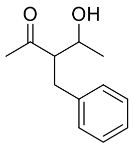

1a

Following procedure A by reacting acetylacetone (75.0 mmol) with benzyl bromide (50.0 mmol) followed by reduction with $NaBH_4$. Purification over SiO_2 (eluent: *n*-pentane/EtOAc 5/1 v/v).

1.25 g, 24% overall yield, colorless oil, *syn:anti* = 1.0:0.9. <u>*Syn*-diastereomer</u>: ^{1}H-NMR (400 MHz, $CDCl_3$): δ 7.26-7.33 (m, 2H), 7.14-7.23 (m, 3H), 4.02-4.10 (m, 1H), 2.85-3.00 (m, 3H), 2.42-2.46 (m, 1H), 1.97 (s, 3H), 1.25 (d, *J* = 6.4 Hz, 3H); ^{13}C-NMR (101 MHz, $CDCl_3$): δ 213.6, 139.6, 128.9, 128.8, 126.6, 67.9, 60.4, 33.5, 32.8, 20.8. <u>*Anti*-diastereomer</u>: ^{1}H-NMR (400 MHz, $CDCl_3$): δ 7.26-7.33 (m, 2H), 7.14-7.23 (m, 3H), 3.86-3.94 (m, 1H), 2.85-3.00 (m, 3H), 2.60 (dd, *J* = 7.5, 4.0 Hz, 1H), 1.89 (s, 3H), 1.26 (d, *J* = 6.4 Hz, 3H); ^{13}C-NMR (101 MHz, $CDCl_3$): δ 214.4, 138.9, 129.0, 128.8, 126.7, 68.7, 60.5, 35.9, 33.0, 22.1.

NMR-data are consistent with literature reports.[3]

*3-Ethyl-4-hydroxy-2-pentanone (**1b**)*

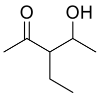

1b

Following procedure A by reacting acetylacetone (75.0 mmol) with ethyl bromide (50.0 mmol) followed by reduction with $NaBH_4$. Purification over SiO_2 (eluent: *n*-pentane/EtOAc 10/1 v/v).

360 mg, 36% overall yield, colorless liquid, *syn:anti* = 1.0:1.0. <u>Mixture of *syn*- and *anti*-diastereomers</u>: ^{1}H-NMR (400 MHz, $CDCl_3$): δ 4.04-3.90 (m, 1H), 2.55-2.42 (m, 1H), 2.20 (s, 3H), 1.77-1.57 (m, 2H), 1.21 (d, *J* = 6.4 Hz, 3H), 1.17 (d, *J* = 6.4 Hz, 3H), 0.92 (t, *J* = 7.5 Hz, 3H), 0.90 (t, *J* = 7.5 Hz, 3H); ^{13}C-NMR (101 MHz, $CDCl_3$): δ 214.2, 213.5, 68.2, 67.8, 60.9, 60.3, 31.8, 31.4, 22.3, 21.7, 20.8, 20.2, 12.5, 11.7.

NMR-data are are consistent with literature reports.[4]

*4-Hydroxy-3-isopropyl-2-pentanone (**1c**)*

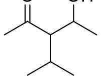

1c

Following procedure A by reacting acetylacetone (50.0 mmol) with isopropyl bromide (150.0 mmol) followed by reduction with $NaBH_4$. Purification over SiO_2 (eluent: *n*-pentane/EtOAc 3/1 v/v).

700 mg, 10% overall yield, colorless liquid, *syn:anti* = 1.0:0.1. *Syn*-diastereomer: ^{1}H-NMR (400 MHz, $CDCl_3$): δ 4.01 (dqd, *J* = 8.5, 6.5, 4.2 Hz, 1H), 2.73-2.79 (m, 1H), 2.32 (dd, *J* = 8.6, 4.2 Hz, 1H), 2.22 (s, 3H), 2.11 (dp, *J* = 9.3, 7.0 Hz, 1H), 1.19 (d, *J* = 6.5 Hz, 3H), 1.02 (d, *J* = 6.7 Hz, 3H), 0.92 (d, *J* = 6.7 Hz, 3H); ^{13}C-NMR (101 MHz, $CDCl_3$): δ 216.2, 66.8, 64.5, 34.6, 28.5, 22.6, 21.0, 20.8. *Anti*-diastereomer: ^{1}H-NMR (400 MHz, $CDCl_3$): δ 4.06-4.13 (m, 1H), 2.73-2.79 (m, 1H), 2.59 (t, *J* = 6.8 Hz, 1H), 2.19 (s, 3H), 2.11 (dp, *J* = 9.3, 7.0 Hz, 1H), 1.18 (d, *J* = 6.3 Hz, 3H), 0.99 (d, *J* = 6.9 Hz, 3H), 0.92 (d, *J* = 6.7 Hz, 3H); ^{13}C-NMR (101 MHz, $CDCl_3$): δ 212.8, 67.6, 64.5, 34.6, 27.5, 21.4, 20.4, 20.3; HRMS (ESI): calc. for $C_8H_{16}O_2Na$ $[M+Na]^+$: 167.1043; found: 167.1040.

*3-(1-Hydroxyethyl)-2-heptanone (**1d**)*

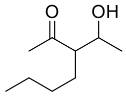

1d

Following procedure A by reacting acetylacetone (50.0 mmol) with *n*-butyl iodide (61.5 mmol) followed by reduction with $NaBH_4$. Purification over SiO_2 (eluent: *n*-pentane/EtOAc 3/1 v/v).

550 mg, 28% overall yield, colorless liquid, *syn:anti* = 1.0:0.4. *Syn*-diastereomer: ^{1}H-NMR (400 MHz, $CDCl_3$): δ 3.93-4.01 (m, 1H), 2.46-2.60 (m, 1H), 2.19 (s, 3H), 1.45-1.75 (m, 2H), 1.22-1.35 (m, 4H), 1.16 (dd, *J* = 6.4, 1.2 Hz, 3H), 0.88 (t, *J* = 7.1 Hz, 3H); ^{13}C-NMR (101 MHz, $CDCl_3$): δ 214.3, 68.0, 58.8, 31.7, 30.3, 29.5, 23.1, 20.8, 14.0. *Anti*-diastereomer: ^{1}H-NMR (400 MHz, $CDCl_3$): δ 3.86-3.93 (m, 1H), 2.46-2.60 (m, 1H), 2.19 (s, 3H), 1.45-1.75 (m, 2H), 1.22-1.35 (m, 4H), 1.24 – 1.17 (m, 3H), 0.88 (t, *J* = 7.1 Hz, 3H); ^{13}C-NMR (101 MHz, $CDCl_3$): δ 213.6, 68.6, 59.5, 31.4, 30.3, 29.0, 23.0, 20.8, 14.0.

NMR-data are consistent with literature reports.[4]

*3-(1-Hydroxyethyl)-5-hexen-2-one (**1e**)*

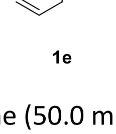

1e

Following procedure A by reacting acetylacetone (50.0 mmol) with allyl bromide (61.5 mmol) followed by reduction with $NaBH_4$. Purification over SiO_2 (eluent: *n*-pentane/EtOAc 3/1 v/v).

250 mg, 17% overall yield, colorless liquid, *syn:anti* = 1.0:1.0. *Syn*-diastereomer: ^{1}H-NMR (400 MHz, $CDCl_3$): δ 5.65-5.85 (m, 1H), 5.00-5.12 (m, 2H), 4.00-4.08 (m, 1H), 2.58-2.70 (m, 1H), 2.31-2.43 (m, 2H), 2.16-2.20 (m, 3H), 1.18 (dd, *J* = 6.4, 1.4 Hz, 3H). *Anti*-diastereomer: ^{1}H-NMR (400 MHz, $CDCl_3$): δ 5.65-5.85 (m, 1H), 5.00-5.12 (m, 2H), 3.94 (h, *J* = 6.3 Hz, 1H), 2.58-2.70 (m, 1H), 2.31-2.43 (m, 2H), 2.16-2.20 (m, 3H), 1.22 (dd,

J = 6.4, 1.3 Hz, 3H); Mixture of *syn*- and *anti*-diastereomers: ^{13}C-NMR (101 MHz, $CDCl_3$): δ 213.4, 212.7, 135.9, 135.9, 134.8, 117.6, 117.1, 68.4, 67.7, 58.9, 58.3, 33.6, 31.8, 31.5, 21.6, 20.7.

NMR-data are consistent with literature reports.[4]

*3-(1-Hydroxyethyl)hex-5-yn-2-one (**1f**)*

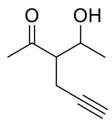

1f

Following procedure A by reacting acetylacetone (80.0 mmol) with propargyl bromide (16.0 mmol) followed by reduction with $NaBH_4$. Purification over SiO_2 (eluent: *n*-pentane/EtOAc 3/2 v/v).

200 mg, 13% overall yield, colorless liquid, *syn:anti* = 1.0:1.4. *Syn*-diastereomer: ^{1}H-NMR (400 MHz, $CDCl_3$): δ 4.07-4.16 (m, 1H), 2.72-2.81 (m, 1H), 2.48-2.58 (m, 2H), 2.30 (s, 3H), 2.00-2.04 (m, 1H), 1.20 (dd, *J* = 6.4, 3.7 Hz, 3H); ^{13}C-NMR (101 MHz, $CDCl_3$): δ 211.2, 82.1, 70.4, 67.3, 57.1, 31.8, 20.7, 16.2. *Anti*-diastereomer: ^{1}H-NMR (400 MHz, $CDCl_3$): δ 3.98-4.07 (m, 1H), 2.72-2.81 (m, 1H), 2.48-2.58 (m, 2H), 2.29 (s, 3H), 2.00-2.04 (m, 1H), 1.24 (dd, *J* = 6.7, 3.4 Hz, 3H); ^{13}C-NMR (101 MHz, $CDCl_3$): δ 211.9, 81.0, 70.7, 68.2, 57.2, 31.9, 21.4, 18.5; HRMS (ESI): calc. for $C_8H_{12}O_2Na$ $[M+Na]^+$: 163.0730; found: 163.0726.

Preparation of β-hydroxyketone 1g

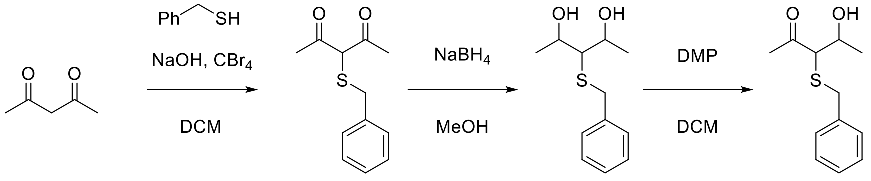

S1 (R^1 = Me) **3g** **S2g** **1g**

To a solution of acetylacetone **S1** (R^1=Me, 3.60 g, 3.7 mL, 36.0 mmol) and NaOH (1.20 g, 30.0 mmol) in DCM (15 mL) tetrabromo methane (9.95 g, 36.0 mmol) was added. The reaction mixture was cooled to 0 °C and a solution of benzyl mercaptane (3.73 g, 3.5 mL, 30.0 mmol) in DCM (30 mL) was added dropwise. The reaction mixture was stirred for 2 h at room temperature and then quenched by pouring the mixture into well stirred ice-water. The phases were separated, and the aqueous phase was extracted with DCM (3 x 50 mL). The combined organic phases were dried over Na_2SO_4, filtered and concentrated. Purification over SiO_2 (eluent: *n*-pentane) afforded the corresponding α-substituted 1,3-diketone **3g**. Compound **3g** was further used in the next step without characterisation.

To a solution of the 1,3-diketone **3g** (1.0 eq.) in MeOH (0.2 M) at 0 °C was added sodium borohydride (3.0 eq.). The resulting reaction mixture was stirred at 0 °C for 30 min and was then allowed to warm to room temperature. After stirring for additional 2 h, the reaction was carefully quenched with sat. aq. NH_4Cl solution (30 mL). The solvent was partially removed under reduced pressure. The resulting mixture was extracted with EtOAc (3 x 30 mL). The combined organic phases were dried over Na_2SO_4,

filtered and concentrated in vacuo. Purification over SiO_2 (eluent: *n*-pentane/EtOAc) to afford the corresponding 1,3-diol **S2g**. Compound **S2g** was further used in the next step without characterisation.

A solution of diol **S2g** (370 mg, 1.6 mmol) in DCM (20 mL) was cooled down to 0 °C. DMP (750 mg, 1.8 mmol) was added in portions at 0 °C and the reaction was stirred at 0 °C for 3 h. The reaction progress was monitored by TLC. Brine (20 mL) was added, and the organic phase was washed with brine (3 x 20 mL) and water (3 x 20 mL). The organic phase was dried over Na_2SO_4, filtered and concentrated. Purification SiO_2 (*n*-pentane/EtOAc 3:1 v/v) afforded the product **1g**.

230 mg, 26% yield over two steps, colorless liquid, *syn:anti* = 1.0:0.8. *Syn*-diastereomer: ^{1}H-NMR (400 MHz, $CDCl_3$): δ 7.23-7.37 (m, 5H), 4.04-4.18 (m, 1H), 3.60-3.86 (m, 2H), 3.12 (d, *J* = 5.2 Hz, 1H), 2.79-2.84 (s br, 1H), 2.20 (s, 3H), 1.30 (d, *J* = 6.3 Hz, 3H). *Anti*-diastereomer: ^{1}H-NMR (400 MHz, $CDCl_3$): δ 7.23-7.37 (m, 5H), 4.04-4.18 (m, 1H), 3.60-3.86 (m, 2H), 3.10 (d, *J* = 7.1 Hz, 1H), 2.44 (d, *J* = 5.1 Hz, 1H), 2.22 (s, 3H), 1.19 (d, *J* = 6.2 Hz, 3H); Mixture of *syn*- and *anti*-diastereomers: ^{13}C-NMR (101 MHz, $CDCl_3$): δ 205.9, 205.3, 137.3, 137.2, 129.3, 128.9, 128.8, 127.7, 127.6, 67.4, 65.6, 60.6, 59.5, 35.7, 35.6, 28.6, 28.1, 20.5, 20.3. HRMS (ESI): calc. for $C_{12}H_{16}O_2SNa$ $[M+Na]^+$: 247.0763; found: 247.0760.

Preparation of β-hydroxyketone 1h

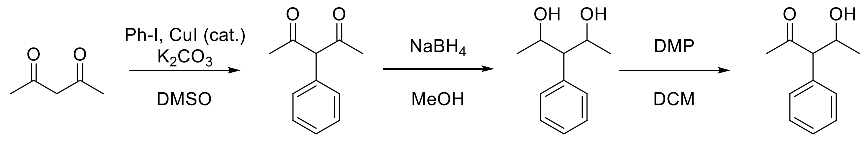

S1 (R^1 = Me) **3h** **S2h** **1h**

CuI (760 mg, 4.00 mmol) was added to a suspension of K_2CO_3 (22.1 g, 160 mmol) in DMSO (40 mL) under an argon atmosphere. Phenyl iodide (8.16 g, 4.5 mL, 40.0 mmol) and acetylacetone **S1** (R^1 = Me, 8.00 g, 8.2 mL, 80.0 mmol) were added. The reaction mixture was stirred for 4 h at 120 °C. The reaction mixture was cooled down to room temperature and poured into hydrochloric acid (1 M, 200 mL). The aqueous phase was extracted with EtOAc (5 x 100 mL). The combined organic phases were dried over Na_2SO_4, filtered and evaporated. Purification over SiO_2 (eluent: *n*-pentane/ethyl acetate) afforded the corresponding α-substituted 1,3-diketone **3h**. Compound **3h** was further used in the next step without characterisation.

To a solution of the 1,3-diketone **3h** (1.0 eq.) in MeOH (0.2 M) at 0 °C was added sodium borohydride (5.0 eq.). The resulting reaction mixture was stirred at 0 °C for 30 min and was then allowed to warm to room temperature. After stirring for additional 2 h, the reaction was carefully quenched with brine (50 mL). The solvent was partially removed under reduced pressure. The resulting mixture was extracted with EtOAc (3 x 50 mL). The combined organic phases were dried over Na_2SO_4, filtered and concentrated in vacuo. Purification over SiO_2 (eluent: *n*-pentane/EtOAc) afforded the corresponding 1,3-diol **S2h**. Compound **S2h** was further used in the next step without characterisation.

A solution of diol **S2h** (4.42 g, 24.5 mmol) in DCM (100 mL) was cooled down to 0 °C. DMP (8.33 g, 19.6 mmol) was added portion wise at 0 °C. The reaction mixture was stirred at room temperature for 3 h. The reaction progress was monitored by TLC. Then EtOAc (150 mL) was added, and the crude mixture was

filtered through silica several times. Purification over SiO_2 (*n*-pentane/EtOAc 20/1 – 1/1 v/v) afforded the product **1h**.

1.14 g, 19% overall yield, colorless oil, *syn:anti* = 1.0:0.4. *Syn*-diastereomer: ^{1}H-NMR (400 MHz, $CDCl_3$): δ 7.24-7.40 (m, 3H), 7.14-7.18 (m, 2H), 4.40-4.47 (m, 1H), 3.61 (d, *J* = 5.4 Hz, 1H), 2.09 (s, 3H), 1.10 (d, *J* = 6.3 Hz, 3H); ^{13}C-NMR (101 MHz, $CDCl_3$): δ 209.7, 134.4, 129.7, 128.9, 127.9, 67.5, 65.5, 30.1, 20.4. *Anti*-diastereomer: ^{1}H-NMR (400 MHz, $CDCl_3$): δ 7.25-7.40 (m, 5H), 4.33-4.40 (m, 1H), 3.57 (d, *J* = 9.2 Hz, 1H), 2.05 (s, 3H), 0.98 (d, *J* = 6.3 Hz, 3H); ^{13}C-NMR (101 MHz, $CDCl_3$): δ 210.1, 135.9, 129.2, 128.7, 127.8, 68.9, 67.8, 29.8, 20.0.

NMR-data are consistent with literature reports.[5]

*5-Hydroxy-4-methylheptan-3-one (**1i**)*

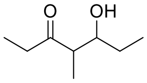

1i

Following procedure A by reacting 3,5-heptanedione (41.6 mmol) with methyl iodide (54.0 mmol) followed by reduction with $NaBH_4$. Purification over SiO_2 (eluent: *n*-pentane/EtOAc 20/1 – 4/1 v/v).

2.70 g, 46% overall yield, colorless liquid, *syn:anti* = 1.0:3.0. *Syn*-diastereomer: ^{1}H-NMR (400 MHz, $CDCl_3$): δ 3.78-3.85 (m, 1H), 2.72 (br s, 1H), 2.40-2.70 (m, 3H), 1.30-1.60 (m, 2H), 1.12 (d, *J* = 7.2 Hz, 3H), 1.05 (t, *J* = 7.3 Hz, 3H), 0.95 (t, *J* = 7.5 Hz, 3H); ^{13}C-NMR (101 MHz, $CDCl_3$): δ 216.9, 72.8, 49.4, 35.2, 27.0, 10.6, 10.0, 7.8. *Anti*-diastereomer: ^{1}H-NMR (400 MHz, $CDCl_3$): δ 3.57-3.65 (m, 1H), 2.72 (br s, 1H), 2.40-2.70 (m, 3H), 1.30-1.60 (m, 2H), 1.12 (d, *J* = 7.2 Hz, 3H), 1.05 (t, *J* = 7.2 Hz, 3H), 0.97 (t, *J* = 7.4 Hz, 3H); ^{13}C-NMR (101 MHz, $CDCl_3$): δ 216.9, 75.1, 50.7, 36.2, 27.7, 14.4, 10.0, 7.7.

NMR-data are consistent with literature reports.[6]

*4-Ethyl-5-hydroxyheptan-3-one (**1j**)*

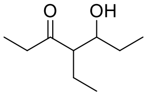

1j

Following procedure A by reacting 3,5-heptanedione (39.0 mmol) with ethyl iodide (27.0 mmol) followed by reduction with $NaBH_4$. Purification over SiO_2 (eluent: *n*-pentane/EtOAc 5/1 v/v).

1.20 g, 29% overall yield, colorless liquid, *syn:anti* = 1.0:0.9. *Syn*-diastereomer: ^{1}H-NMR (400 MHz, $CDCl_3$): δ 3.64-3.72 (m, 1H), 2.43-2.60 (m, 3H), 1.58-1.80 (m, 2H), 1.34-1.55 (m, 2H), 1.04 (t, *J* = 7.2 Hz, 3H), 0.95 (t, *J* = 7.4 Hz, 3H), 0.90 (t, *J* = 7.5 Hz, 3H); *Anti*-diastereomer: ^{1}H-NMR (400 MHz, $CDCl_3$): δ 3.57-3.66 (m, 1H), 2.43-2.60 (m, 3H), 1.58-1.80 (m, 2H), 1.34-1.55 (m, 2H), 1.05 (t, *J* = 7.2 Hz, 3H), 0.97 (t, *J* = 7.4 Hz, 3H), 0.89 (t, *J* = 7.5 Hz, 3H); Mixture of *syn*- and *anti*-diastereomers: ^{13}C-NMR (101 MHz, $CDCl_3$): δ 217.4, 216.4,

73.8, 73.3, 57.8, 57.6, 38.3, 38.0, 28.6, 27.6, 22.8, 19.9, 12.6, 12.1, 10.6, 10.3, 7.5, 7.4; HRMS (ESI): calc. for $C_9H_{18}O_2Na$ $[M+Na]^+$: 181.1199; found: 181.1197.

*4-Benzyl-5-hydroxyheptan-3-one (**1k**)*

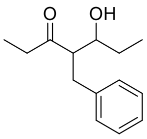

1k

Following procedure A by reacting 3,5-heptanedione (30.0 mmol) with benzyl bromide (20.0 mmol) followed by reduction with $NaBH_4$. Purification over SiO_2 (eluent: *n*-pentane/EtOAc 10/1 v/v).

1.60 g, 35% overall yield, colorless liquid, *syn:anti* = 1.0:1.1. *Syn*-diastereomer: ^{1}H-NMR (400 MHz, $CDCl_3$): δ 7.23-7.31 (m, 2H), 7.10-7.22 (m, 3H), 3.70-3.77 (m, 1H), 2.85-3.0 (3H), 2.69 (d, *J* = 3.1 Hz, 1H), 2.20-2.35 (m, 1H), 1.85 (dq, *J* = 18.5, 7.2 Hz, 1H), 1.38-1.64 (m, 2H), 1.00 (t, *J* = 7.4 Hz, 3H), 0.87 (t, *J* = 7.2 Hz, 3H); *Anti*-diastereomer: ^{1}H-NMR (400 MHz, $CDCl_3$): δ 7.23-7.31 (m, 2H), 7.10-7.22 (m, 3H), 3.52-3.63 (m, 1H), 2.85-3.0 (3H), 2.77 (d, *J* = 8.5 Hz, 1H), 2.20-2.35 (m, 1H), 2.04 (dq, *J* = 18.5, 7.2 Hz, 1H), 1.38-1.64 (m, 2H), 0.97 (t, *J* = 7.4 Hz, 3H), 0.81 (t, *J* = 7.2 Hz, 3H); Mixture of *syn*- and *anti*-diastereomers: ^{13}C-NMR (101 MHz, $CDCl_3$): δ 217.5, 216.4, 139.8, 139.1, 129.0, 128.7, 126.6, 126.5, 74.4, 73.2, 58.2, 57.7, 39.4, 38.8, 36.4, 33.4, 29.0, 27.5, 10.6, 10.5, 7.1; HRMS (ESI): calc. for $C_{14}H_{20}O_2Na$ $[M+Na]^+$: 243.1356; found: 243.1347.

Preparation of β-hydroxyketone 1l

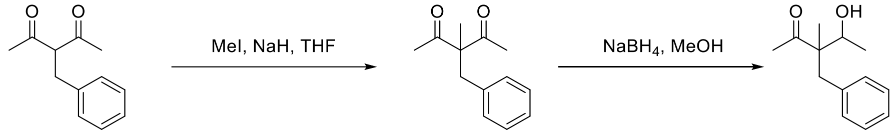

3a **3l** **1l**

NaH (380 mg, 60 w%, 9.46 mmol) was suspended in dry THF (26 mL) and cooled down to 0 °C. Diketone **3a** (1.50 g, 7.88 mmol) was added as a solution in dry THF (5 mL) and the reaction was stirred for 40 min at 0 °C before methyl iodide (2.24 g, 1.0 mL, 15.8 mmol) was added dropwise. The reaction mixture was allowed to warm to room temperature and was stirred for 3 h (reaction progress was monitored by TLC) and then quenched with sat. aq. NH_4Cl solution. The aqueous phase was extracted with Et_2O (3 x 20 mL). The combined organic phases were dried over Na_2SO_4, filtered and concentrated. Purification over SiO_2 (eluent: *n*-pentane/ethyl acetate) afforded the corresponding α-substituted 1,3-diketone **3l**. Compound **3l** was further used in the next step without characterisation.

The 1,3-diketone **3l** obtained was reduced with $NaBH_4$ following procedure A. 450 mg, 23% overall yield, colorless liquid, *syn:anti* = 1.0:1.2. Mixture of *syn*- and *anti*-diastereomers: ^{1}H-NMR (400 MHz, $CDCl_3$): δ 7.31-7.16 (m, 3H), 7.16-7.07 (m, 2H), 3.98 (qd, *J* = 6.4, 4.4 Hz, 1H), 3.80 (dq, *J* = 8.0, 6.5 Hz, 1H), 3.21 (d, *J* = 13.3 Hz, 1H), 3.00 (d, *J* = 13.2 Hz, 1H), 2.83 (d, *J* = 8.0 Hz, 1H), 2.79 (d, *J* = 13.3 Hz, 1H), 2.70 (d, *J* = 13.3 Hz, 1H), 2.43 (d, *J* = 4.4 Hz, 1H), 2.02 (s, 3H), 1.93 (s, 3H), 1.19 (d, *J* = 6.3 Hz, 3H), 1.17 (s, 3H), 1.14 (s, 3H); ^{13}C-NMR (101 MHz, $CDCl_3$): δ 217.2, 215.7, 137.6, 136.8, 130.4, 130.3, 128.6, 126.8, 126.7, 72.3, 71.7, 56.4, 56.0, 42.6, 40.9, 29.4, 29.3, 18.5, 17.7, 17.1; HRMS (ESI): calc. for $C_{13}H_{18}O_2Na$ $[M+Na]^+$: 229.1199; found: 229.1202.

Due to complicated isolation, compound **1l** was obtained in ≈85% purity (see the NMR data of the isolated compound).

Preparation of β-hydroxyketone 1m

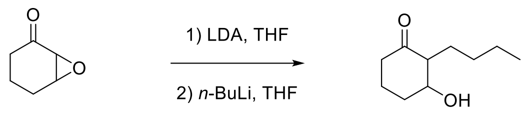

S3 **1m**, 72%

n-Butyl lithium (4.3 mL, 2.5 M in hexane, 10.7 mmol) was added to a solution of diisopropylamine (1.30 g, 1.8 mL, 12.8 mmol) in THF (40 mL) at -78 °C. After stirring for 10 min at -78 °C, epoxide **S3** (1.00 g, 8.92 mmol) was added dropwise and the reaction mixture was stirred for 20 min at -78 °C. Subsequently, *n*-butyl lithium (8.9 mL, 2.5 M in hexane, 22.3 mmol) was added dropwise and the reaction was allowed to warm to -20 °C. The reaction mixture was stirred at -20 °C for 2 h. After addition of sat. aq. NH_4Cl solution, the aqueous phase was extracted with Et_2O (3 x 50 mL). The combined organic fractions were washed with brine (2 x 20 mL), dried over Na_2SO_4, filtered and concentrated. Purification over SiO_2 (*n*-pentane/EtOAc 3/1 v/v) afforded the product **1m**.

1.10 g, 72% yield, colorless oil, *syn:anti* = 1.0:10.0. *Syn*-diastereomer: ^{1}H-NMR (400 MHz, $CDCl_3$): δ 4.34-4.38 (m, 1H), 2.22-2.45 (m, 3H), 1.95-2.15 (m, 2H), 1.51-1.80 (m, 4H), 1.15-1.44 (m, 4H), 0.89 (t, *J* = 7.0 Hz, 3H). *Anti*-diastereomer: ^{1}H-NMR (400 MHz, $CDCl_3$): δ 3.77 (td, *J* = 8.0, 3.6 Hz, 1H), 2.22-2.45 (m, 3H), 1.95-2.15 (m, 2H), 1.51-1.80 (m, 4H), 1.15-1.44 (m, 4H), 0.89 (t, *J* = 7.0 Hz, 3H); ^{13}C-NMR (101 MHz, $CDCl_3$): δ 207.1, 74.1, 59.5, 40.4, 32.3, 30.0, 26.6, 23.1, 21.1, 14.1.

NMR-data are consistent with literature reports.[7]

Preparation of separated diastereomers of β-hydroxyketone 1a

*anti-3-Benzyl-4-hydroxy-2-pentanone (**1a**)*

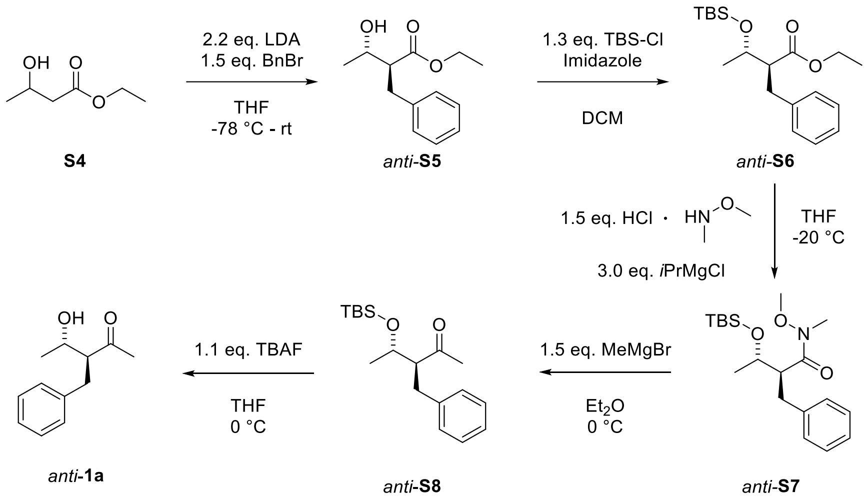

n-Butyl lithium (13.2 mL, 2.5 M in hexane, 33.0 mmol) was added to a solution of diisopropylamine (3.80 g, 5.3 mL, 37.5 mmol) in THF at -78 °C. After stirring for 10 min, ester **S4** (2.00 g, 2.0 mL, 15.0 mmol) was added dropwise and the reaction mixture was allowed to warm to -20 °C and stirred at -20 °C for 30 min. Then, the reaction was cooled down to -78 °C and benzyl bromide (3.90 g, 2.7 mL, 22.5 mmol) was added. The resulting reaction mixture was slowly warmed to room temperature and stirred for further 60 min before being quenched with sat. aq. ammonium chloride solution (20 mL). The phases were separated, and the aqueous phase was extracted with Et_2O (3 x 30 mL). The combined organic phases were dried over NaSO4, filtered and concentrated. Purification over SiO_2 (eluent: *n*-pentane/EtOAc 30/1 v/v) afforded α-substituted β-hydroxyester *anti*-**S5** (*syn:anti* > 1:20). Compound *anti*-**S5** was further used in the next step without characterisation.

TBSCl (2.60 g, 17.0 mmol) was added to a solution of β-hydroxyester **S5** (2.89 g, 13.0 mmol) and imidazole (1.80 g, 26.0 mmol) in DCM (50 mL) at 0 °C. The reaction mixture was allowed to warm to room temperature and stirred for 16 h. Then, Et_2O (50 mL) was added, and the mixture was filtered and evaporated. Purification over SiO_2 (eluent: *n*-pentane/EtOAc 20/1 v/v) afforded the corresponding TBS-protected ester *anti*-**S6**. Compound *anti*-**S6** was further used in the next step without characterisation.

*i*PrMgCl (8.5 mL, 2.0 M in Et_2O, 17.0 mmol) was added dropwise at -20 °C to a solution of protected β-hydroxyester **S6** (1.90 g, 5.6 mmol) and *N,O*-dimethylhydroxylamine hydrochloride (830 mg, 8.5 mmol) in dry THF (30 mL). The reaction mixture was stirred at -20 °C for 3 h (reaction progress monitored by TLC). Then sat. aq. NH_4Cl solution (30 mL) was added and the aq. phase was extracted with Et_2O (3 x 30 mL). The combined organic phases were dried over Na_2SO_4, filtered and concentrated. Purification over SiO_2 (eluent: *n*-pentane/EtOAc 10/1 v/v) afforded the corresponding Weinreb amide *anti*-**S7**. Compound *anti*-**S7** was further used in the next step without characterisation.

MeMgBr (3.0 M in Et_2O, 2.5 mL, 7.2 mmol) was added over 30 min at 0 °C to a solution of Weinreb amide **S7** (1.70 g, 4.8 mmol) in dry Et_2O (20 mL). The resulting mixture was stirred at 0 °C for 1 h. The reaction mixture was allowed to warm to room temperature and was stirred for further 18 h. Then, the reaction was quenched with sat. aq. NH_4Cl solution (20 mL) and extracted with Et_2O (3 x 25 mL). The combined organic phases were dried over Na_2SO_4, filtered and concentrated. Purification over SiO_2 (eluent: *n*-pentane/EtOAc 20/1 v/v) afforded the corresponding TBS-protected β-hydroxyketone *anti*-**S8**. Compound *anti*-**S8** was further used in the next step without characterisation.

TBAF (1.0 M in THF, 1.8 mL, 1.80 mmol) was added dropwise at 0 °C to a solution of protected β-HK *anti*-**S8** (500 mg, 1.60 mmol) in dry THF (10 mL). The resulting mixture was stirred at 0 °C for 3 h (reaction progress monitored by TLC). Then brine (30 mL) was added, and the phases were separated. The aqueous phase was extracted with Et_2O (3 x 25 mL). The combined organic phases were dried over Na_2SO_4, filtered and concentrated. Purification over SiO_2 (eluent: *n*-pentane/EtOAc 3/1 v/v) afforded the product *anti*-**1a**.

250 mg, 30% overall yield, clear oil. ^{1}H-NMR (400 MHz, $CDCl_3$): δ 7.26-7.33 (m, 2H), 7.14-7.23 (m, 3H), 3.86-3.94 (m, 1H), 2.85-3.00 (m, 3H), 2.60 (dd, *J* = 7.5, 4.0 Hz, 1H), 1.89 (s, 3H), 1.26 (d, *J* = 6.4 Hz, 3H); ^{13}C-NMR (101 MHz, $CDCl_3$): δ 214.4, 138.9, 129.0, 128.8, 126.7, 68.7, 60.5, 35.9, 33.0, 22.1.

NMR-data are consistent with literature reports.[3]

*syn-3-Benzyl-4-hydroxy-2-pentanone (**1a**)*

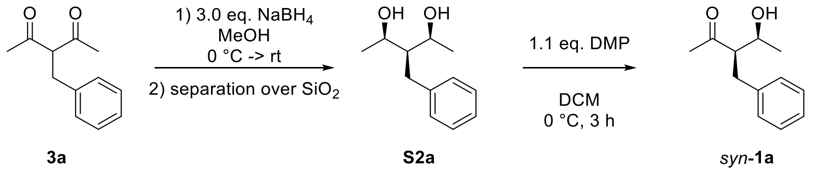

A solution of diketone **3a** (2.18 g, 11.5 mmol) in MeOH (5 mL) was added dropwise at 0 °C to a solution of sodium borohydride (1.30 g, 34.0 mmol) in MeOH (30 mL). The resulting reaction mixture was stirred for 1 h at 0 °C (reaction progress monitored by TLC). Then, acetone (20 mL) and sat. aq. NH_4Cl solution (20 mL) were added and the mixture was stirred for 1 h at room temperature. The solvent was evaporated, and the aqueous phase was extracted with EtOAc (3 x 50 mL). The combined organic phases were dried over Na_2SO_4, filtered and concentrated. Crude diol **S2a** was separated over Si_2O (eluent: *n*-pentane/EtOAc 6/4 v/v).

DMP (570 mg, 1.35 mmol) was added portion wise at 0 °C to a solution of diol **S2a** (250 mg, 1.29 mmol) in DCM (7 mL). The resulting reaction mixture was stirred for 30 min at 0 °C. The reaction mixture was allowed to warm to room temperature and stirred for 3 h (reaction progress monitored by TLC). EtOAc (30 mL) and sat. aq. $NaHCO_3$ solution (30 mL) were added. The phases were separated, and the organic phase was washed with sat. aq. sodium bicarbonate solution (3 x 30 mL) and brine (3 x 30 mL). The organic phase was dried over Na_2SO_4, filtered and concentrated. Purification over SiO_2 (eluent: *n*-pentane/EtOAc 3/1 v/v) afforded the product *syn*-**1a**.

50 mg, 20% overall yield, clear oil. ^{1}H-NMR (400 MHz, $CDCl_3$): δ 7.26-7.33 (m, 2H), 7.14-7.23 (m, 3H), 4.02-4.10 (m, 1H), 2.85-3.00 (m, 3H), 2.42-2.46 (m, 1H), 1.97 (s, 3H), 1.25 (d, *J* = 6.4 Hz, 3H); ^{13}C-NMR (101 MHz, $CDCl_3$): δ 213.6, 139.6, 128.9, 128.8, 126.6, 67.9, 60.4, 33.5, 32.8, 20.8.

NMR-data are consistent with literature reports.[3]

Optimization of the reaction conditions for formation of 2d*

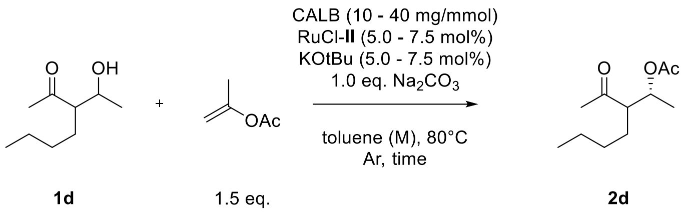

Entry	CALB [mg/mmol]	RuCl-**II** [mol%]**	Time [h]	M	NMR-yield***	*syn*-**2d**: *anti*-**2d** ****	*ee* [%] ****
1	40	5.0	20	0.2	98	50:50	99
2	40	5.0	20	0.1	96	40:60	99
3	20	5.0	20	0.2	85	40:60	99
4	10	5.0	46	0.2	72	35:65	99
5	40	7.5	20	0.2	99	40:60	99
6	40	7.5	20	0.1	95	40:60	99
7	40	7.5	40	0.05	86	40:60	99
8	20	7.5	20	0.2	85	40:60	99

*Reactions conducted using 0.2 mmol of β-HK **1d**. **Activation with equimolar amounts KO*t*Bu-solution (0.1 M in toluene). ***NMR-yield using mesitylene as the internal standard. ****determined by GC on chiral stationary phase.

Preparation of β-oxoacetates 2a-2m

General procedure B for synthesis of racemic β-oxoacetates 2a-2m

DMAP (1.1 eq.) and acetic anhydride (2.0 eq.) were added to a solution of the β-HK (1.0 eq.) in DCM (0.1 M). The resulting mixture was stirred at room temperature for 16 h and concentrated in vacuo. Purification over SiO_2 (eluent: *n*-pentane/EtOAc 20/1 – 5/1 v/v) afforded the corresponding β-oxoacetates.

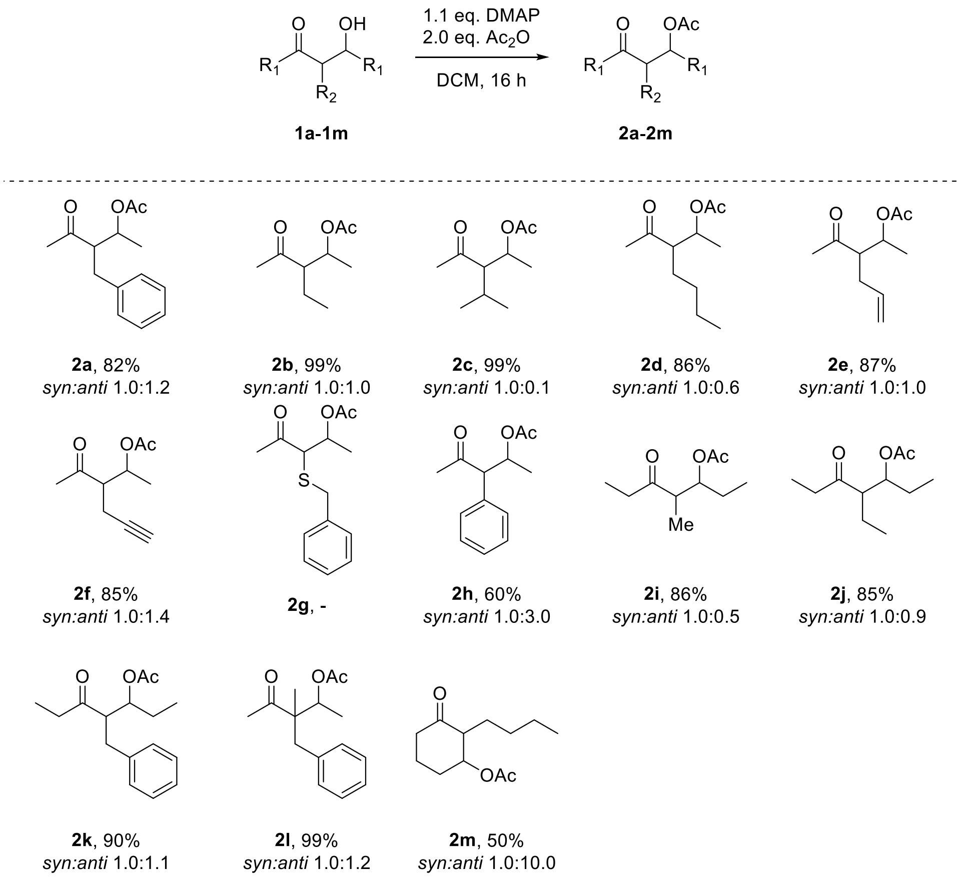

Scheme S1. Preparation of racemic β-oxoacetates **2a-2m**, *d.r.* was determined by ^{1}H-NMR analysis.

DYKAT of β-hydroxyketones 1a-1m

General procedures C and D

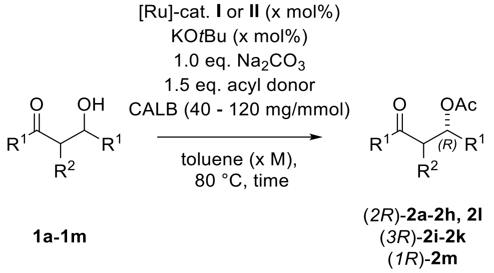

a) with ruthenium complex I (procedure C)

A dry Schlenk flask was charged with β-HK (0.2 mmol) and acyl donor (0.3 mmol). After addition of dry toluene (1 mL), ruthenium complex **I** (2.5 mol%), Na_2CO_3 (0.2 mmol) and CALB (16 mg) were added and the Schlenk flask was repeatedly carefully evacuated and refilled with argon. The reaction mixture was stirred under argon atmosphere at 80 °C. After the reaction was complete (monitored using ^{1}H-NMR analysis), the reaction mixture was filtered and crude β-oxoacetate was purified SiO_2 (eluent: *n*-pentane/EtOAc).

b) with ruthenium complex II (procedure D)

Conditions A:

To a dry Schlenk flask were added ruthenium chloride complex **II** (5.0 mol%), Na_2CO_3 (0.2 mmol), CALB (x mg/mmol) and dry toluene (0.3 mL). A solution of KO*t*Bu (0.1 M in toluene, 5.0 mol%) was added. The resulting mixture was stirred at room temperature for 5 min. The β-HK (0.2 mmol) was added as a solution in dry toluene (0.7 mL) and the resulting mixture was stirred for 5 min at room temperature. The acyl donor (0.3 mmol) was added, and the reaction mixture was stirred under argon atmosphere at 80 °C. After the reaction was complete (monitored using ^{1}H-NMR analysis), the reaction mixture was filtered and crude β-oxoacetate was purified over SiO_2 (eluent: *n*-pentane/EtOAc).

Conditions B:

To a dry Schlenk flask were added ruthenium chloride complex **II** (7.5 mol%), Na_2CO_3 (0.2 mmol), CALB (40 mg/mmol) and dry toluene (0.3 mL). A solution of KO*t*Bu (0.1 M in toluene, 7.5 mol%) was added. The resulting mixture was stirred at room temperature for 5 min. The β-HK (0.2 mmol) was added as a solution in dry toluene (0.7 mL) and the resulting mixture was stirred for 5 min at room temperature. The acyl donor (0.3 mmol) and dry toluene (1.0 mL) were added, and the reaction mixture was stirred under argon atmosphere at 80 °C. After the reaction was complete (monitored using ^{1}H-NMR analysis), the reaction mixture was filtered and crude β-oxoacetate was purified over SiO_2 (eluent: *n*-pentane/EtOAc).

*3-Benzyl-4-oxopentan-2-yl acetate (**2a**)*

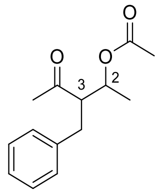

2a

Conditions A: Following the general procedure D (using 40 mg/mmol CALB and the reaction time was 20 h), β-oxoacetate **2a** (42 mg, 90%, *syn:anti* = 35:65, 99% *ee* for both diastereomers) was isolated as a colorless oil.

Conditions B: Following the general procedure D (using 40 mg/mmol CALB and the reaction time was 20 h), β-oxoacetate **2a** (42 mg, 90%, *syn:anti* = 35:65, 99% *ee* for both diastereomers) was isolated as a colorless oil.

Syn-diastereomer: ^{1}H-NMR (400 MHz, $CDCl_3$): δ 7.24-7.30 (m, 2H), 7.17-7.22 (m, 1H), 7.10-7.15 (m, 2H), 5.13 (dq, J = 13.2, 6.4 Hz, 1H), 3.06-3.17 (m, 1H), 2.90 (dt, J = 13.5, 10.6 Hz, 1H), 2.79 (dd, J = 13.5, 4.8 Hz, 1H), 2.05 (s, 3H), 1.97 (s, 3H), 1.30 (d, J = 6.4 Hz, 3H); ^{13}C-NMR (101 MHz, $CDCl_3$): δ 209.4, 170.3, 139.1, 128.9, 128.7, 126.6, 71.1, 58.6, 34.6, 32.6, 21.3, 17.6. *Anti*-diastereomer: ^{1}H-NMR (400 MHz, $CDCl_3$): δ 7.24-7.30 (m, 2H), 7.17-7.22 (m, 1H), 7.10-7.15 (m, 2H), 5.13 (dq, J = 13.2, 6.4 Hz, 1H), 3.06-3.17 (m, 1H), 2.90 (dt, J = 13.5, 10.6 Hz, 1H), 2.79 (dd, J = 13.5, 4.8 Hz, 1H), 2.01 (s, 3H), 1.95 (s, 3H), 1.25 (d, J = 6.4 Hz, 3H); ^{13}C-NMR (101 MHz, $CDCl_3$): δ 209.4, 170.1, 139.1, 128.9, 128.8, 126.6, 71.2, 59.3, 33.9, 31.7, 21.3, 17.7; HRMS (ESI): calc. for $C_{14}H_{18}O_3Na$ $[M+Na]^+$: 257.1148; found: 257.1148.

GC: IVADEX-I, 60-1.0°C/min-90-0.1°C/min-115-20°C/min-200, t_R: 236.3 min for (2*S*,3*R*)-diastereomer, 239.9 min (2*S*,3*S*)-diastereomer, 250.1 min for (2*R*,3*R*)-diastereomer and 256.5 min for (2*R*,3*S*)-diastereomer.

Racemate:

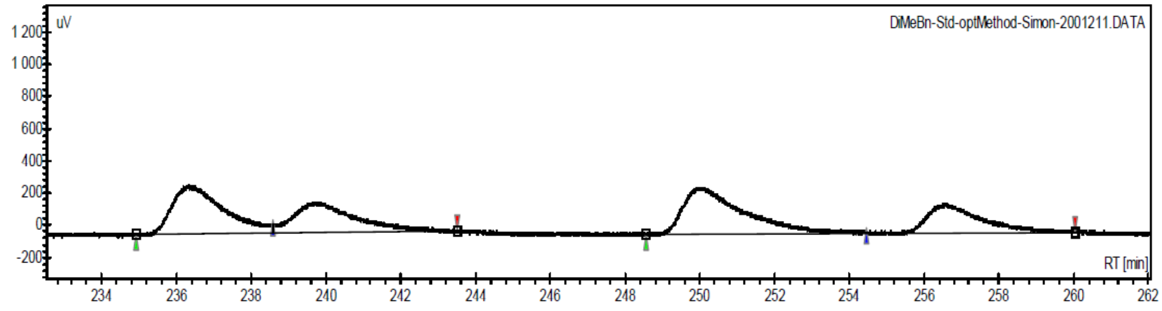

DiMeBn-Std-optMethod-Simon-2001211.DATA [FID]

Index	Time [Min]	Quantity [% Area]	Height [uV]	Area [uV.Min]	Area % [%]
1	236.34	28.24	290.9	472.2	28.237
3	239.85	21.87	179.6	365.8	21.874
2	250.07	32.42	287.5	542.2	32.419
4	256.53	17.47	175.7	292.2	17.470
Total		100.00	933.7	1672.4	100.000

Enantiomer (Reaction conditions A):

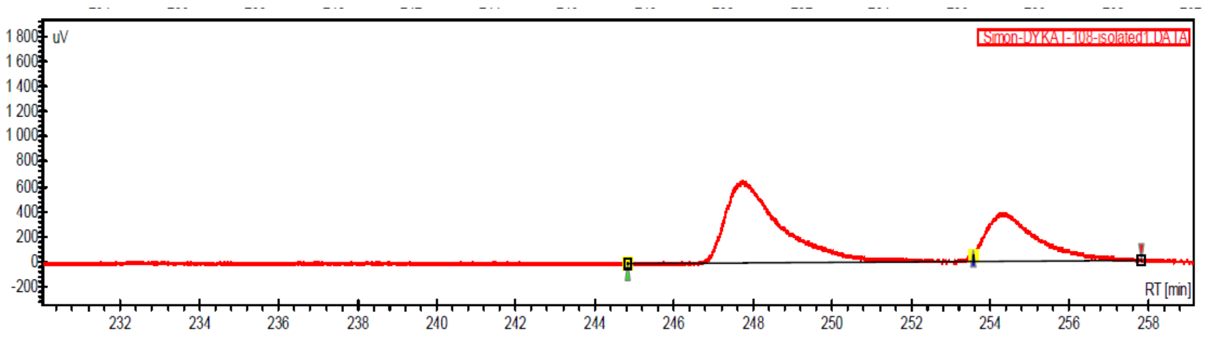

Simon-DYKAT-108-isolated1.DATA [FID]

Index	Time [Min]	Quantity [% Area]	Height [uV]	Area [uV.Min]	Area % [%]
1	247.73	65.02	648.0	1096.8	65.017
2	254.31	34.98	380.9	590.1	34.983
Total		100.00	1028.9	1686.9	100.000

Enantiomer (Reaction conditions B):

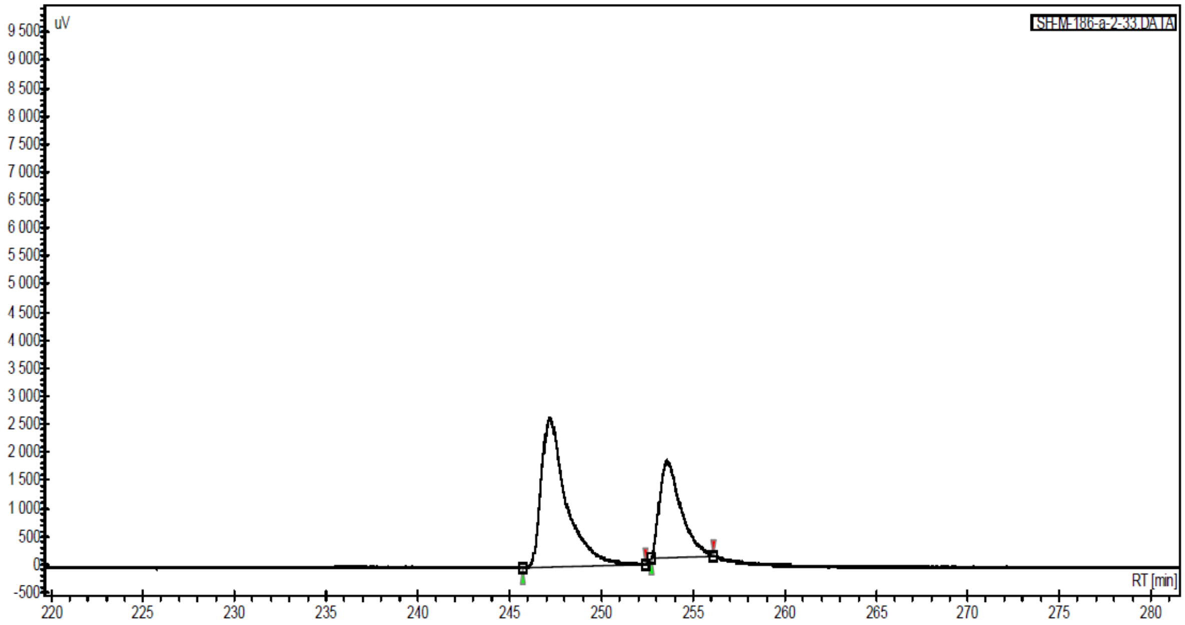

SH-M-186-a-2-33.DATA [FID]

Index	Time [Min]	Quantity [% Area]	Height [uV]	Area [uV.Min]	Area % [%]
1	247.17	64.85	2680.2	4237.1	64.855
2	253.58	35.15	1710.8	2296.2	35.145
Total		100.00	4391.0	6533.3	100.000

*3-Ethyl-4-oxopentan-2-yl acetate (**2b**)*

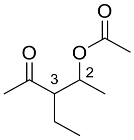

2b

Conditions A: Following the general procedure D (using 40 mg/mmol CALB and the reaction time was 20 h), β-oxoacetate **2b** (32 mg, 93%, *syn:anti* = 50:50, 98% *ee* (*syn*), 98% *ee* (*anti*)) was isolated as a colorless oil.

Conditions B**:** Following the general procedure D (using 40 mg/mmol CALB and the reaction time was 20 h), β-oxoacetate **2b** (33 mg, 97%, *syn:anti* = 45:55, 99% *ee* for both diastereomers) was isolated as a colorless oil.

Mixture of *syn*- and *anti*-diastereomers: ^{1}H-NMR (400 MHz, $CDCl_3$): δ 5.16-5.01 (m, 1H), 2.68 (ddd, J = 9.5, 6.7, 4.2 Hz, 1H), 2.61 (ddd, J = 9.8, 7.8, 4.4 Hz, 1H), 2.17 (s, 3H), 2.15 (s, 3H), 2.04 (s, 3H), 2.00 (s, 3H), 1.76-1.43 (m, 2H), 1.21 (d, J = 6.3 Hz, 3H), 0.91-0.81 (m, 3H); ^{13}C-NMR (101 MHz, $CDCl_3$): δ 210.0, 209.9, 170.4, 170.2, 71.2, 70.7, 59.5, 58.7, 31.5, 30.2, 21.3, 21.0, 17.8, 17.6, 11.9, 11.8; HRMS (ESI): calc. for $C_9H_{16}O_3Na$ $[M+Na]^+$: 195.0992; found: 195.0993.

GC: IVADEX-I, 60-0.2°C/min-80-20.0°C/min-200, t_R: 47.7 min for and 49.1 min for (2*S*)-diastereomers, 58.6 min for (2*R*,3*R*)-diastereomer and 65.3 min for (2*R*,3*S*)-diastereomer.

Racemate:

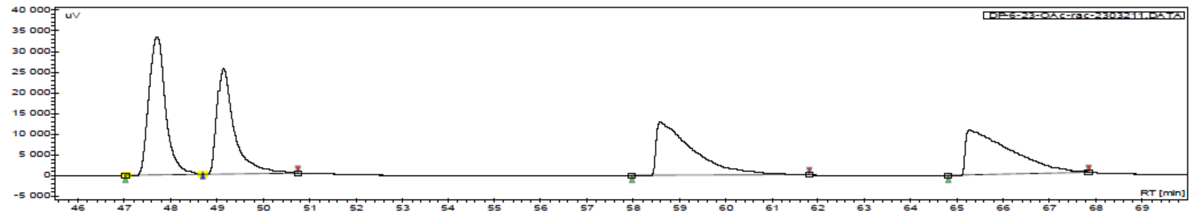

DP-6-23-OAc-rac-2303211.DATA [FID]

Index	Time [Min]	Quantity [% Area]	Height [uV]	Area [uV.Min]	Area % [%]
1	47.72	28.10	33226.2	13634.0	28.097
2	49.14	22.63	25462.2	10980.9	22.630
3	58.60	24.19	12881.4	11739.5	24.193
4	65.27	25.08	10762.5	12169.5	25.079
Total		100.00	82332.4	48523.9	100.000

Enantiomer (Reaction conditions A):

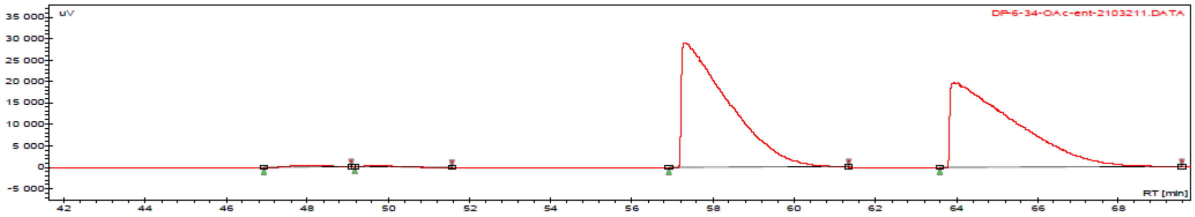

DP-6-34-OAc-ent-2103211.DATA [FID]

Index	Time [Min]	Quantity [% Area]	Height [uV]	Area [uV.Min]	Area % [%]
3	48.26	0.50	322.4	380.7	0.501
4	49.62	0.38	406.5	290.2	0.382
1	57.30	51.21	29237.3	38917.1	51.211
2	63.96	47.91	19817.1	36405.6	47.906
Total		100.00	49783.4	75993.5	100.000

Enantiomer (Reaction conditions B):

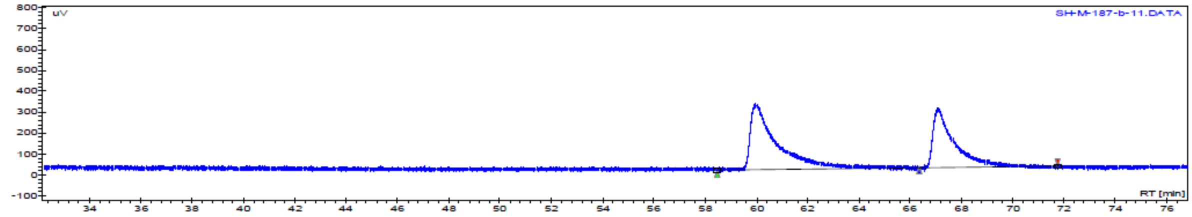

SH-M-187-b-11.DATA [FID]

Index	Time [Min]	Quantity [% Area]	Height [uV]	Area [uV.Min]	Area % [%]
1	59.97	57.13	304.3	367.6	57.134
2	67.09	42.87	278.4	275.8	42.866
Total		100.00	582.7	643.4	100.000

*3-Acetyl-4-methylpentan-2-yl acetate (**2c**)*

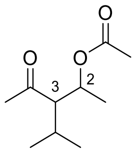

2c

Conditions A: Following the general procedure D (using 40 mg/mmol CALB and the reaction time was 20 h), β-oxoacetate **2c** (24 mg, 65%, *syn:anti* = 37:63, 99% *ee* for both diastereomers) was isolated as a colorless oil.

Conditions B: Following the general procedure D (using 40 mg/mmol CALB and the reaction time was 40 h), β-oxoacetate **2c** (28 mg, 75%, *syn:anti* = 35:65, 99% *ee* for both diastereomers) was isolated as a colorless oil.

Syn-diastereomer: ^{1}H-NMR (400 MHz, $CDCl_3$): δ 5.17 (p, *J* = 6.5 Hz, 1H), 2.41 (dd, *J* = 7.9, 6.6 Hz, 1H), 2.17 (s, 3H), 1.93-2.05 (m, 4H), 1.21 (d, *J* = 6.4 Hz, 3H), 0.98 (d, *J* = 6.8 Hz, 3H), 0.89 (d, *J* = 6.8 Hz, 3H); ^{13}C-NMR (101 MHz, $CDCl_3$): δ 210.4, 170.2, 69.8, 64.1, 31.7, 27.1, 21.3, 21.2, 19.6, 18.4. *Anti*-diastereomer: ^{1}H-NMR (400 MHz, $CDCl_3$): δ 5.17 (p, *J* = 6.5 Hz, 1H), 2.71 (dd, *J* = 8.8, 6.6 Hz, 1H), 2.17 (s, 3H), 1.93-2.05 (m, 4H), 1.18 (d, *J* = 6.4 Hz, 3H), 0.96 (d, *J* = 7.0 Hz, 3H), 0.89 (d, *J* = 6.8 Hz, 3H); ^{13}C-NMR (101 MHz, $CDCl_3$): δ 210.7, 170.2, 70.6, 61.8, 34.5, 28.0, 21.5, 20.8, 20.4, 16.0; HRMS (ESI): calc. for $C_{10}H_{18}O_3Na$ $[M+Na]^+$: 209.1148; found: 209.1143.

GC: Hydrodex β-DM, 50-1.0°C/min-170, t_R: 24.4 min for (2*S*,3*S*)-diastereomer, 24.8 min for (2*R*,3*R*)-diastereomer, 26.2 min for (2*S*,3*R*)- diastereomer and 26.8 min for (2*R*,3*S*)-diastereomer.

Racemate:

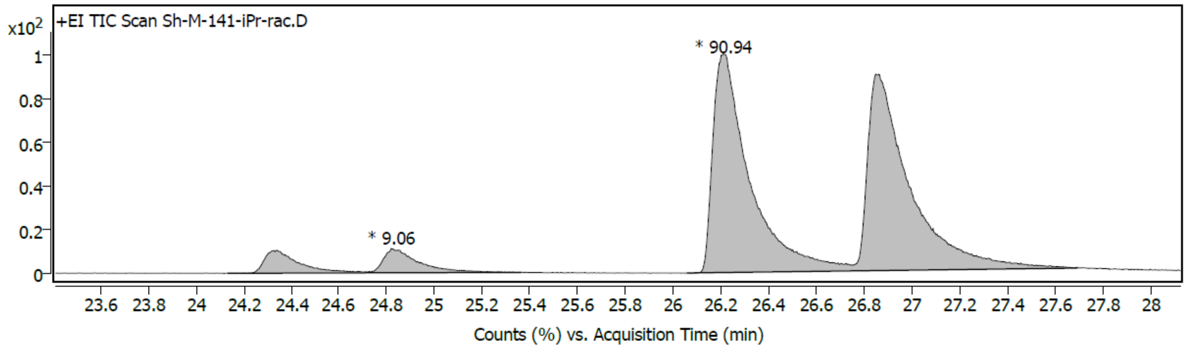

Enantiomer (Reaction conditions A):

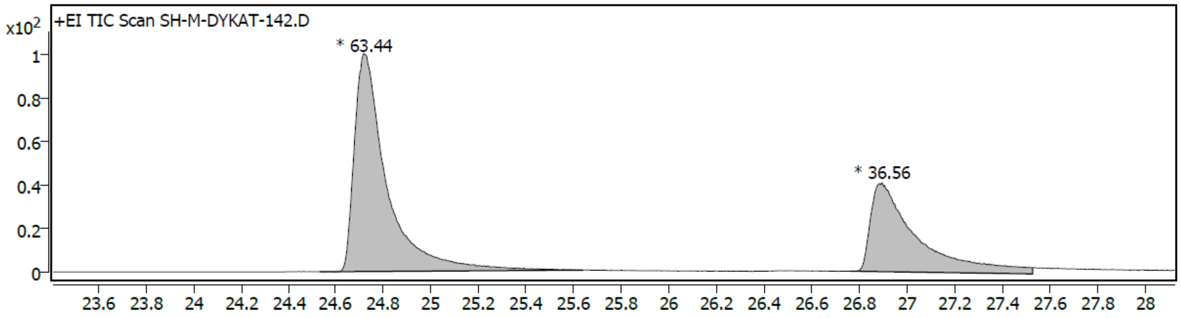

Enantiomer (Reaction conditions B):

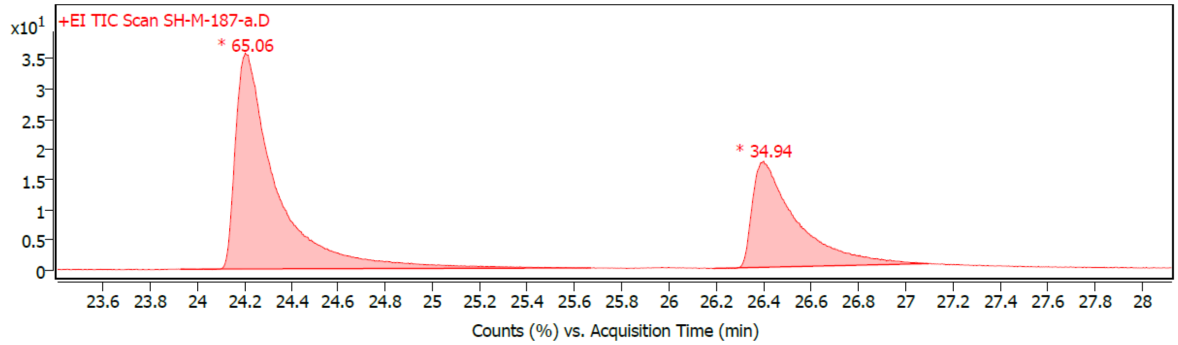

*3-Acetylheptan-2-yl acetate (**2d**)*

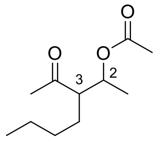

2d

Conditions A: Following the general procedure D (using 40 mg/mmol CALB and the reaction time was 20 h), β-oxoacetate **2d** (38 mg, 95%, *syn:anti* = 45:55, 99% *ee* for both diastereomers) was isolated as a colorless oil.

Conditions B: Following the general procedure D (using 40 mg/mmol CALB and the reaction time was 20 h), β-oxoacetate **2d** (39 mg, 98%, *syn:anti* = 40:60, 99% *ee* for both diastereomers) was isolated as a colorless oil.

Syn-diastereomer: ^{1}H-NMR (400 MHz, $CDCl_3$): δ 5.00-5.10 (m, 1H), 2.73 (ddd, *J* = 9.7, 6.6, 4.1 Hz, 1H), 2.16 (s, 3H), 2.03 (s, 3H), 1.53-1.70 (m, 1H), 1.36-1.47 (m, 1H), 1.12-1.32 (m, 7H), 0.85 (t, *J* = 7.2 Hz, 3H); ^{13}C-NMR (101 MHz, $CDCl_3$): δ 209.9, 170.3, 70.9, 57.1, 31.5, 29.7, 27.8, 22.9, 21.3, 17.5, 13.9. *Anti*-diastereomer: ^{1}H-NMR (400 MHz, $CDCl_3$): δ 5.00-5.10 (m, 1H), 2.66 (ddd, *J* = 10.1, 7.8, 4.2 Hz, 1H), 2.14 (s, 3H), 1.99 (s, 3H), 1.53-1.70 (m, 1H), 1.36-1.47 (m, 1H), 1.12-1.32 (m, 7H), 0.86 (t, *J* = 7.2 Hz, 3H); ^{13}C-NMR (101 MHz, $CDCl_3$): δ 210.2, 170.2, 71.4, 58.0, 30.1, 29.5, 27.6, 22.9, 21.3, 17.8, 14.0; HRMS (ESI): calc. for $C_{11}H_{20}O_3Na$ $[M+Na]^+$: 223.1305; found: 223.1306.

GC: Hydrodex β-DM, 50-1.0°C/min-170, t_R: 37.8 min for (2*R*,3*S*)- diastereomer, 38.3 min for (2*R*,3*R*)-diastereomer, 38.6 min for (2*S*,3*S*)-diastereomer and 39.1 min for (2*S*,3*R*)-diastereomer.

Racemate:

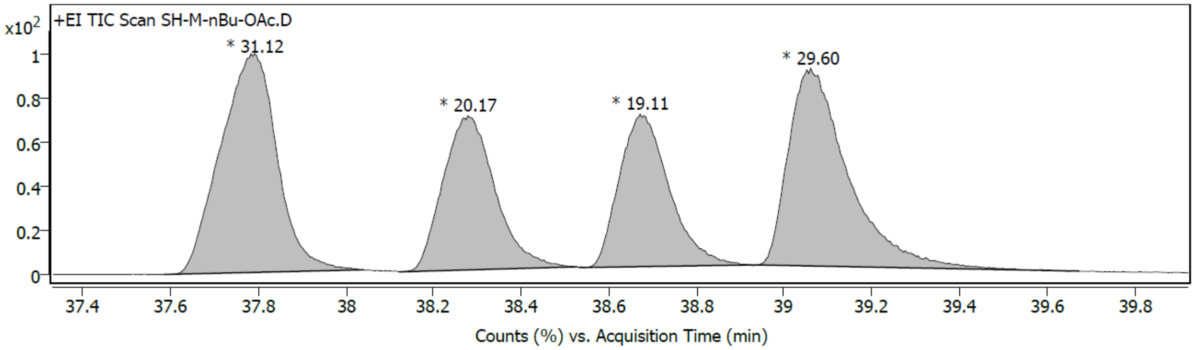

Enantiomer (Reaction conditions A):

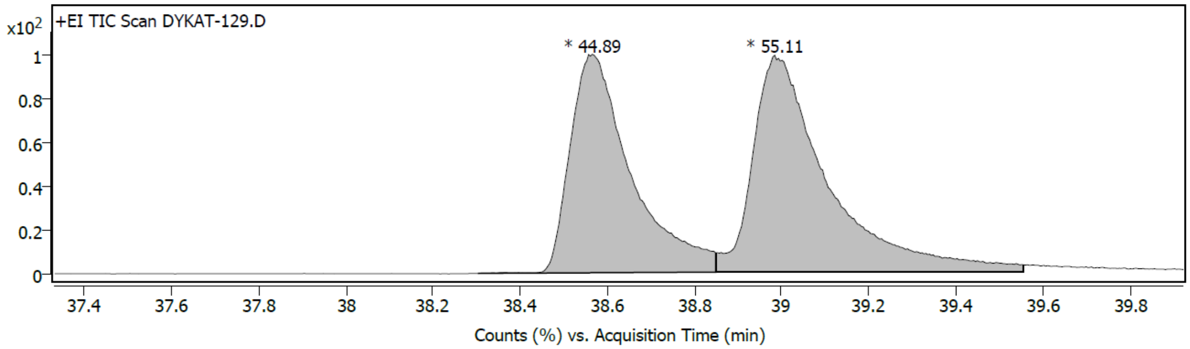

Enantiomer (Reaction conditions B):

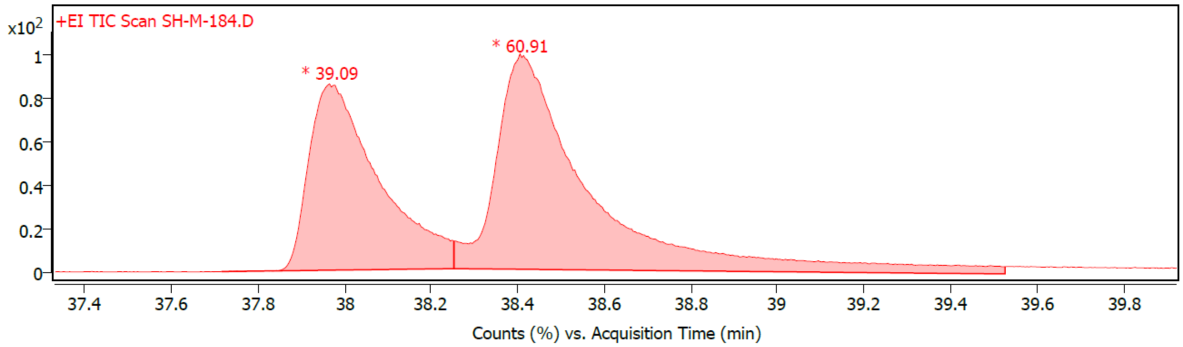

*3-Acetylhex-5-en-2-yl acetate (**2e**)*

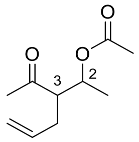

2e

Conditions A: Following the general procedure D (using 40 mg/mmol CALB and the reaction time was 20 h), β-oxoacetate **2e** (30 mg, 80%, *syn:anti* = 50:50, 99% *ee* for both diastereomers) was isolated as a colorless oil.

Conditions B: Following the general procedure D (using 40 mg/mmol CALB and the reaction time was 20 h), β-oxoacetate **2e** (35 mg, 95%, *syn:anti* = 37:63, 99% *ee* for both diastereomers) was isolated as a colorless oil.

Mixture of *syn-* and *anti-*diastereomers: ^{1}H-NMR (400 MHz, $CDCl_3$): δ 5.62-5.75 (m, 1H), 4.98-5.15 (m, 3H), 2.76-2.88 (m, 1H), 2.20-2.45 (m, 2H), 2.17 (s, 3H), 2.15 (s, 3H), 2.05 (s, 3H), 2.01 (s, 3H), 1.23 (d, J = 6.4 Hz, 3H), 1.19 (d, J = 6.4 Hz, 3H); ^{13}C-NMR (101 MHz, $CDCl_3$): δ 209.1, 209.0, 170.3, 170.2, 135.0, 134.6, 117.6, 117.3, 70.9, 70.5, 57.4, 56.6, 32.4, 32.1, 31.6, 30.6, 21.3, 17.7, 17.6; HRMS (ESI): calc. for $C_{10}H_{16}O_3Na$ $[M+Na]^+$: 207.0992; found: 207.0997.

GC: Hydrodex β-DM, 50-1.0°C/min-170, t_R: 28.3 min and 28.6 min for (2*S*)-diastereomers, 30.2 min for (2*R*,3*S*)-diastereomer and 30.7 min for (2*R*,3*R*)-diastereomer.

Racemate:

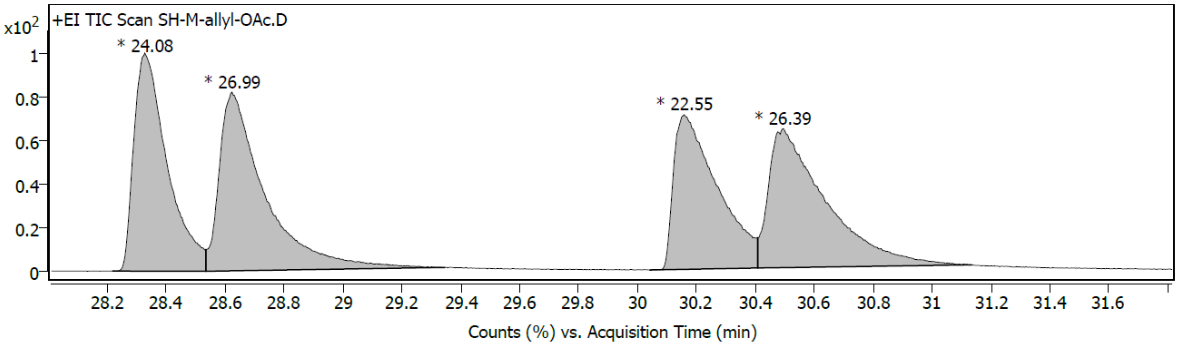

Enantiomer (Reaction conditions A):

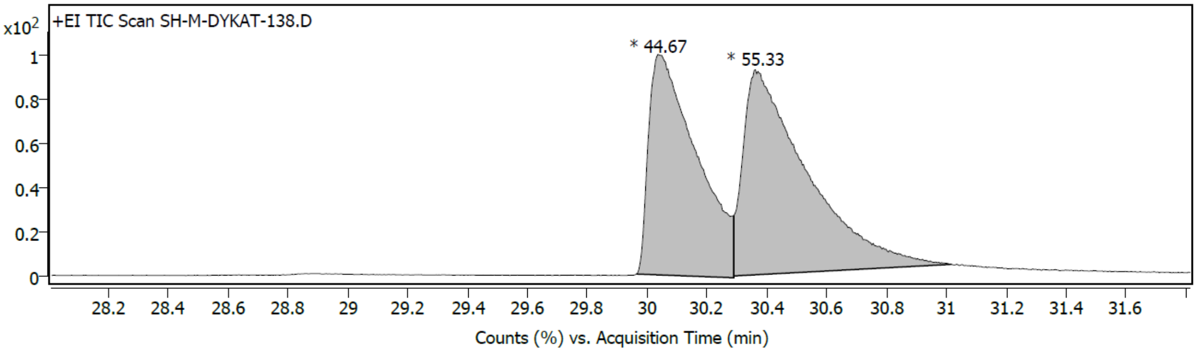

Enantiomer (Reaction conditions B):

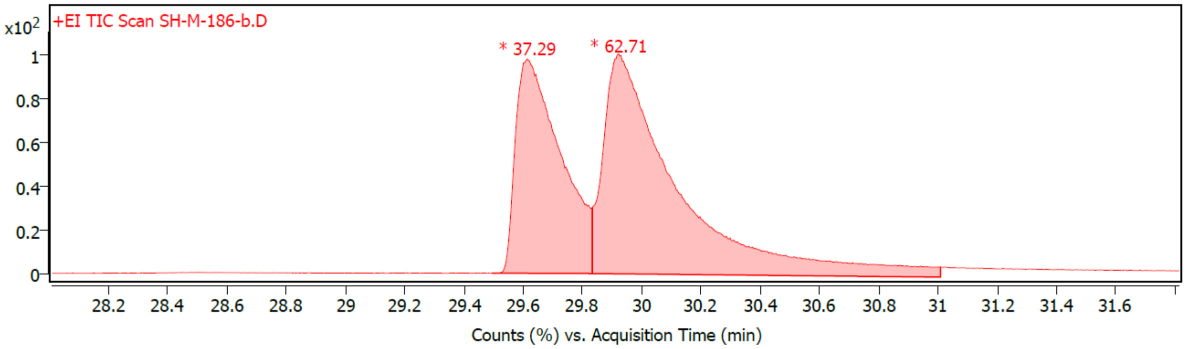

*3-Acetylhex-5-yn-2-yl acetate (**2f**)*

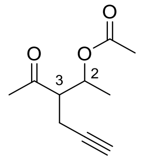

2f

Conditions A: Following the general procedure D (using 40 mg/mmol CALB and the reaction time was 20 h), β-oxoacetate **2f** (13 mg, 45%, *syn:anti* = 37:63, 82% *ee* (*syn*), 48% *ee* (*anti*)) was isolated as a colorless oil.

Syn-diastereomer: ^{1}H-NMR (400 MHz, $CDCl_3$): δ 5.21 (p, *J* = 6.2 Hz, 1H), 2.92-3.00 (m, 1H), 2.46-2.57 (m, 1H), 2.32-2.42 (m, 1H), 2.27 (s, 3H), 2.02 (s, 3H), 1.97-2.01 (m, 1H), 1.22 (d, *J* = 6.4 Hz, 3H); ^{13}C-NMR (101 MHz, $CDCl_3$): δ 207.6, 170.1, 81.2, 70.3, 69.9, 55.8, 31.5, 21.3, 17.6, 17.2. *Anti*-diastereomer: ^{1}H-NMR (400 MHz, $CDCl_3$): δ 5.15 (p, *J* = 6.4 Hz, 1H), 2.92-3.00 (m, 1H), 2.46-2.57 (m, 1H), 2.32-2.42 (m, 1H), 2.27 (s, 3H), 2.02 (s, 3H), 1.97-2.01 (m, 1H), 1.22 (d, *J* = 6.4 Hz, 3H); ^{13}C-NMR (101 MHz, $CDCl_3$): δ 207.5, 170.1, 81.0, 70.4, 70.1, 56.0, 30.8, 21.3, 17.2, 16.9; HRMS (ESI): calc. for $C_{10}H_{14}O_3Na$ $[M+Na]^+$: 205.0835; found: 205.0839.

GC: IVADEX-I, 60-0.4°C/min-90-20°C/min-200, t_R: 70.0 min for (2*S*,3*R*)-diastereomer, 71.3 min for (2*S*,3*S*)-diastereomer, 76.4 min for (2*R*,3*R*)-diastereomer and 77.1 min for (2*R*,3*S*)-diastereomer.

Racemate:

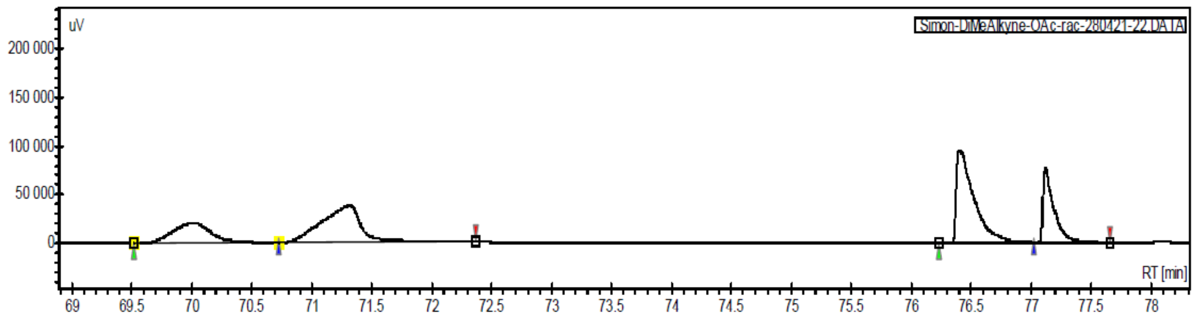

Simon-DiMeAlkyne-OAc-rac-280421-22.DATA [FID]

Index	Time [Min]	Quantity [% Area]	Height [uV]	Area [uV.Min]	Area % [%]
1	70.01	15.91	20256.9	7445.6	15.914
2	71.31	31.37	37715.7	14678.5	31.373
3	76.39	35.05	94165.1	16398.7	35.050
4	77.11	17.66	77205.9	8264.4	17.664
Total		100.00	229343.5	46787.2	100.000

Enantiomer (Reaction conditions A)

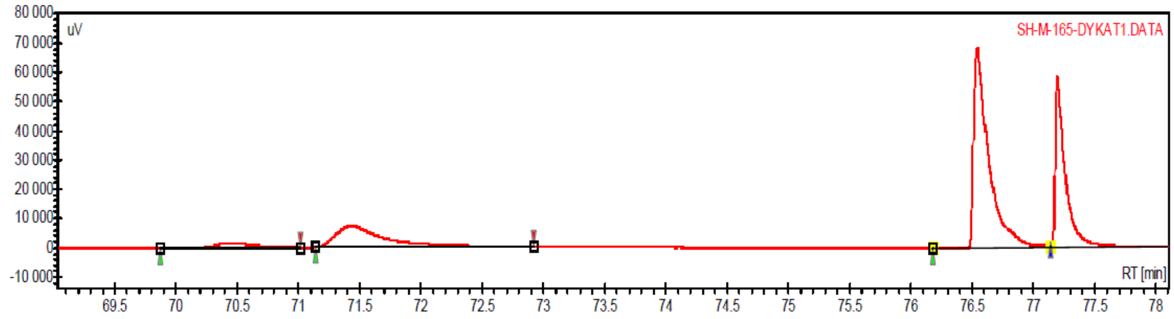

SH-M-165-DYKAT1.DATA [FID]

Index	Time [Min]	Quantity [% Area]	Height [uV]	Area [uV.Min]	Area % [%]
3	70.45	2.73	1566.7	529.8	2.734
4	71.43	17.49	7260.6	3389.2	17.489
1	76.54	50.47	68072.8	9781.4	50.474
2	77.19	29.30	58249.0	5678.8	29.303
Total		100.00	135149.1	19379.2	100.000

*4-Oxo-3-phenylpenta-2-yl acetate (**2h**)*

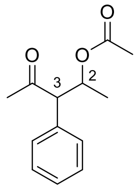

2h

Conditions A: Following the general procedure D (using 40 mg/mmol CALB and the reaction time was 20 h), β-oxoacetate **2h** (12 mg, 27%, *syn:anti* = 15:85, 99% *ee* for both diastereomers) was isolated as a colorless oil.

Syn-diastereomer: ^{1}H-NMR (400 MHz, $CDCl_3$): δ 7.27-7.38 (m, 3H), 7.21-7.25 (m, 2H), 5.50-5.62 (m, 1H), 3.80 (d, J = 8.8 Hz, 1H), 2.09 (s, 3H), 2.01 (s, 3H), 1.01 (d, J = 6.3 Hz, 3H); ^{13}C-NMR (101 MHz, $CDCl_3$): δ 206.1, 170.1, 135.3, 129.2, 128.9, 127.9, 70.4, 64.7, 30.2, 21.0, 19.3. *Anti*-diastereomer: ^{1}H-NMR (400 MHz, $CDCl_3$): δ 7.27-7.38 (m, 3H), 7.21-7.25 (m, 2H), 5.50-5.62 (m, 1H), 3.85 (d, J = 10.0 Hz, 1H), 2.10 (s, 3H), 2.01 (s, 3H), 1.01 (d, J = 6.3 Hz, 3H); ^{13}C-NMR (101 MHz, $CDCl_3$): δ 205.7, 170.2, 134.6, 129.3, 129.0, 128.2, 71.2, 64.4, 29.9, 21.3, 17.9; HRMS (ESI): calc. for $C_{13}H_{16}O_3Na$ $[M+Na]^+$: 243.0992; found: 243.0998.

GC: Hydrodex β-DM, 50-1.0°C/min-170, t_R: 60.0 min for (2*S*,3*R*)-diastereomer, 60.3 min for (2*R*,3*S*)-diastereomer, 65.1 min for (2*R*,3*R*)-diastereomer and 65.9 min for (2*S*,3*S*)-diastereomer.

Racemate:

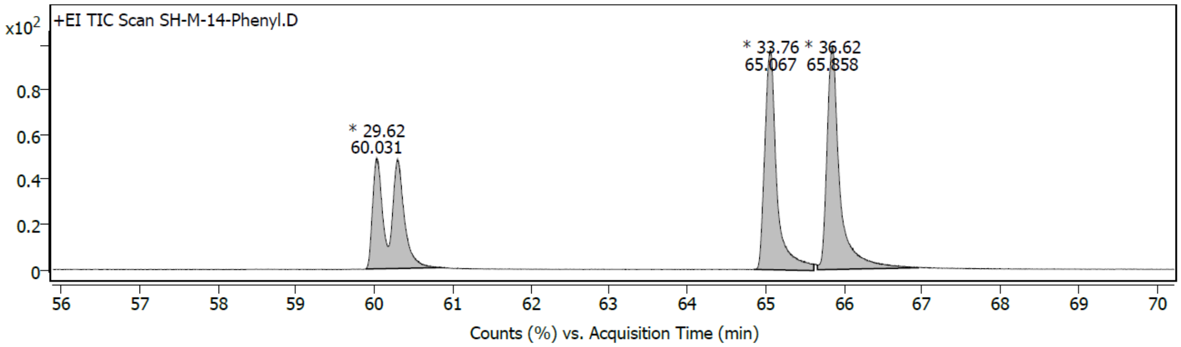

Enantiomer (Reaction conditions A)

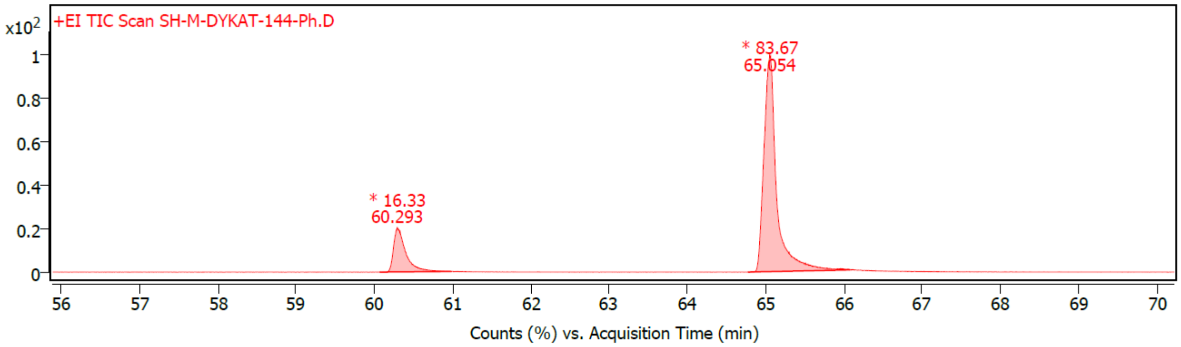

*4-Methyl-5-oxoheptan-3-yl acetate (**2i**)*

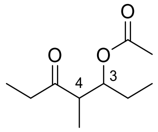

2i

<u>Conditions A</u>: Following the general procedure D (using 80 mg/mmol CALB and the reaction time was 48 h), β-oxoacetate **2i** (28 mg, 75%, *syn:anti* = 60:40, 92% *ee* (*syn*), 98% *ee* (*anti*)) was isolated as a colorless oil.

<u>*Syn*-diastereomer</u>: ^{1}H-NMR (400 MHz, $CDCl_3$): δ 5.07-5.15 (m, 1H), 2.70-2.79 (m, 1H), 2.35-2.60 (m, 2H), 2.03 (s, 3H), 1.46-1.58 (m, 2H), 1.07 (d, J = 6.7 Hz, 3H), 1.03 (t, J = 7.0 Hz, 3H), 0.88 (t, J = 7.4 Hz, 3H); ^{13}C-NMR (101 MHz, $CDCl_3$): δ 212.2, 170.8, 75.4, 49.2, 35.4, 25.3, 21.1, 11.6, 10.2, 7.8. <u>*Anti*-diastereomer</u>: ^{1}H-NMR (400 MHz, $CDCl_3$): δ 5.00-5.07 (m, 1H), 2.81-2.89 (m, 1H), 2.35-2.60 (m, 2H), 2.01 (s, 3H), 1.46-1.58 (m, 2H), 1.07 (d, J = 6.7 Hz, 3H), 1.03 (t, J = 7.0 Hz, 3H), 0.88 (t, J = 7.4 Hz, 3H); ^{13}C-NMR (101 MHz, $CDCl_3$): δ 212.4, 170.5, 75.9, 49.1, 35.1, 23.8, 21.1, 12.4, 9.3, 7.8; HRMS (ESI): calc. for $C_{10}H_{18}O_3Na$ $[M+Na]^+$: 209.1148; found: 209.1143.

GC: IVADEX-I, 60-0.2°C/min-80-20°C/min-200, t_R: 71.9 min for (3*S*,4*R*)-diastereomer, 76.1 min for (3*S*,4*S*)-diastereomer, 82.0 min for (3*R*,4*R*)-diastereomer and 85.9 min for (3*R*,4*S*)-diastereomer.

Racemate:

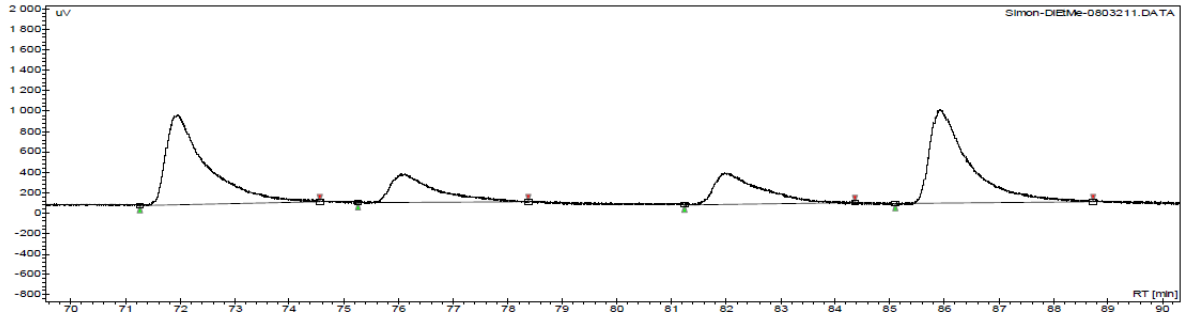

Simon-DiEtMe-0803211.DATA [FID]

Index	Time [Min]	Quantity [% Area]	Height [uV]	Area [uV.Min]	Area % [%]
1	71.93	36.38	872.9	763.3	36.383
2	76.05	12.81	275.8	268.7	12.808
3	82.01	14.17	302.6	297.3	14.168
4	85.93	36.64	912.9	768.7	36.640
Total		100.00	2364.3	2098.1	100.000

Enantiomer (Reaction conditions A):

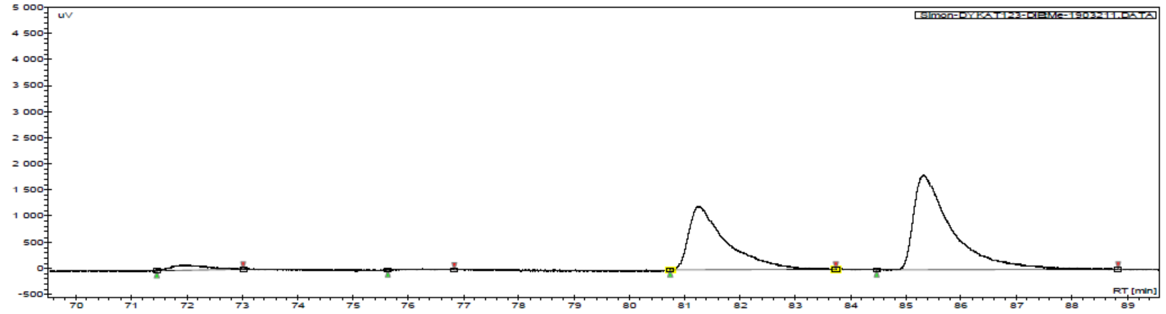

Simon-DYKAT123-DiEtMe-1903211.DATA [FID]

Index	Time [Min]	Quantity [% Area]	Height [uV]	Area [uV.Min]	Area % [%]
4	71.92	2.78	102.7	69.7	2.780
3	76.10	0.30	15.5	7.4	0.295
1	81.26	38.97	1224.2	977.7	38.973
2	85.32	57.95	1810.7	1453.8	57.952
Total		100.00	3153.1	2508.6	100.000

*4-Ethyl-5-oxo-heptan-3-yl acetate (**2j**)*

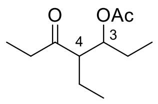

2j

Conditions A: Following the general procedure D (using 120 mg/mmol CALB and the reaction time was 48 h), β-oxoacetate **2j** (19 mg, 48%, *syn:anti* = 44:56, 99% *ee* (*syn*), 98% *ee* (*anti*)) was isolated as a colorless oil.

Mixture of *syn-* and *anti*-diastereomers: ^{1}H-NMR (400 MHz, $CDCl_3$): δ 4.96-5.06 (m, 1H), 2.65-2.75 (m, 1H), 2.30-2.60 (m, 2H), 2.05 (d, J = 1.2 Hz, 3H), 2.01 (d, J = 1.2 Hz, 3H), 1.41-1.75 (m, 4H), 0.99-1.06 (m, 3H), 0.79-0.89 (m, 6H); ^{13}C-NMR (101 MHz, $CDCl_3$): δ 212.6, 212.3, 170.7, 170.4, 75.4, 75.3, 56.8, 37.5, 36.7, 25.1, 24.5, 21.1, 21.0, 12.1, 10.2, 9.4, 7.6; HRMS (ESI): calc. for $C_{11}H_{20}O_3Na$ $[M+Na]^+$: 223.1305; found: 223.1298.

GC: IVADEX-I, 60-0.2°C/min-80-20°C/min-200, t_R: 88.8 min for (3*S*,4*R*)-diastereomer and 93.8 for (3*S*,4*S*)-diastereomers, 101.0 min for (3*R*,4*R*)-diastereomer and 101.7 min for (3*R*,4*S*)-diastereomer.

Racemate:

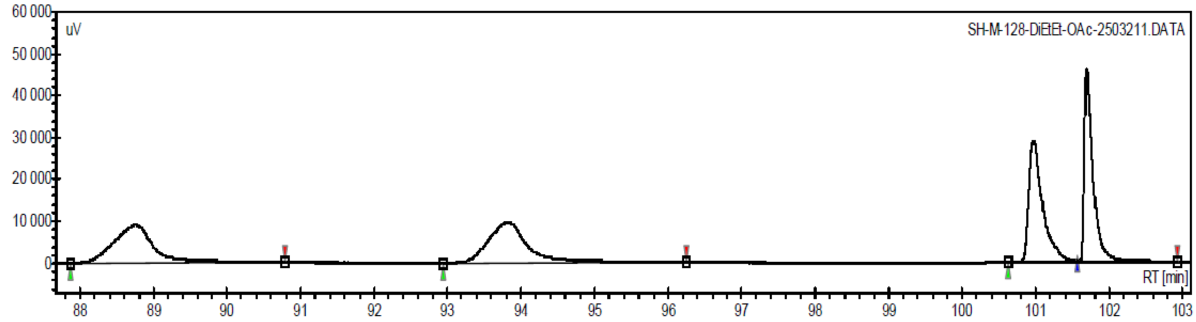

SH-M-128-DiEtEt-OAc-2503211.DATA [FID]

Index	Time [Min]	Quantity [% Area]	Height [uV]	Area [uV.Min]	Area % [%]
1	88.77	23.42	9119.4	5822.7	23.417
2	93.83	24.75	9731.3	6152.9	24.745
3	100.98	25.96	29172.8	6456.1	25.964
4	101.70	25.87	46168.3	6433.5	25.873
Total		100.00	94191.7	24865.1	100.000

Enantiomer (Reaction conditions A):

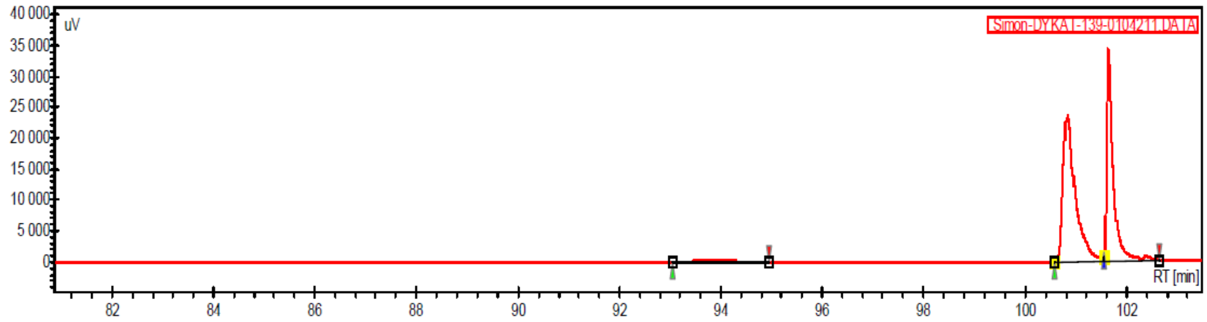

Simon-DYKAT-139-0104211.DATA [FID]

Index	Time [Min]	Quantity [% Area]	Height [uV]	Area [uV.Min]	Area % [%]
3	93.74	0.80	119.5	94.5	0.799
1	100.84	55.53	23557.8	6569.0	55.528
2	101.63	43.67	34064.5	5166.4	43.672
Total		100.00	57741.8	11829.9	100.000

*4-Benzyl-5-oxoheptan-3-yl acetate **(2k)***

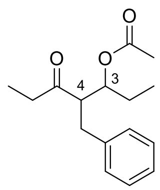

2k

<u>Conditions A</u>: Following the general procedure D (using 120 mg/mmol CALB and the reaction time was 72 h), β-oxoacetate **2k** (26 mg, 50%, *syn:anti* = 20:80, 99% *ee* for both diastereomers) was isolated as a colorless oil.

<u>*Syn*-diastereomer</u>: ^{1}H-NMR (400 MHz, $CDCl_3$): δ 7.23-7.29 (m, 2H), 7.16-7.20 (m, 1H), 7.08-7.14 (m, 2H), 5.04-5.10 (m, 1H), 3.14-3.20 (m, 1H), 2.92 (ddd, *J* = 15.6, 13.3, 10.5 Hz, 2H), 2.29-2.42 (m, 1H), 2.03 (s, 3H), 1.86-2.01 (m, 1H), 1.50-1.82 (m, 2H), 0.84 (dt, *J* = 9.5, 7.5 Hz, 6H); <u>*Anti*-diastereomer</u>: ^{1}H-NMR (400 MHz, $CDCl_3$): δ 7.23-7.29 (m, 2H), 7.16-7.20 (m, 1H), 7.08-7.14 (m, 2H), 5.10-5.16 (m, 1H), 3.08-3.13 (m, 1H), 2.77 (dt, *J* = 13.3, 4.7 Hz, 2H), 2.29-2.42 (m, 1H), 2.08 (s, 3H), 1.86-2.01 (m, 1H), 1.50-1.82 (m, 2H), 0.84 (t, *J* = 7.2 Hz, 6H). <u>Mixture of *syn*- and *anti*-diastereomers</u>: ^{13}C-NMR (101 MHz, $CDCl_3$): δ 211.9, 211.8, 170.7, 170.4, 139.4, 139.1, 128.9, 128.7, 128.6, 126.5, 75.5, 57.1, 56.9, 38.5, 38.1, 34.4, 34.1, 25.2, 24.3, 21.1, 10.2, 9.4, 7.3; HRMS (ESI): calc. for $C_{16}H_{22}O_3Na$ $[M+Na]^+$: 285.1461; found: 285.1459.

GC: IVADEX-I, 60-0.5°C/min-100-0.1°C/min-130-20°C/min-200, t_R: 280.3 min for (3*S*,4*R*)-diastereomer, 283.2 min for (3*S*,4*S*)-diastereomer, 289.5 min for (3*R*,4*S*)-diastereomer and 292.3 min for (3*R*,4*R*)-diastereomer.

Racemate:

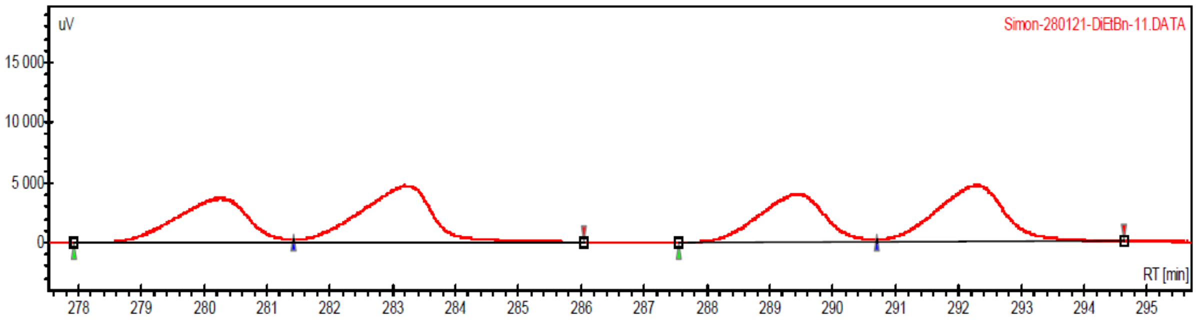

Simon-280121-DiEtBn-11.DATA [FID]

Index	Time [Min]	Quantity [% Area]	Height [uV]	Area [uV.Min]	Area % [%]
1	280.26	22.33	3683.1	4884.7	22.332
2	283.20	28.52	4750.8	6237.4	28.517
3	289.45	22.06	4005.2	4824.3	22.056
4	292.31	27.09	4719.1	5926.4	27.095
Total		100.00	17158.1	21872.9	100.000

Enantiomer (Reaction conditions A):

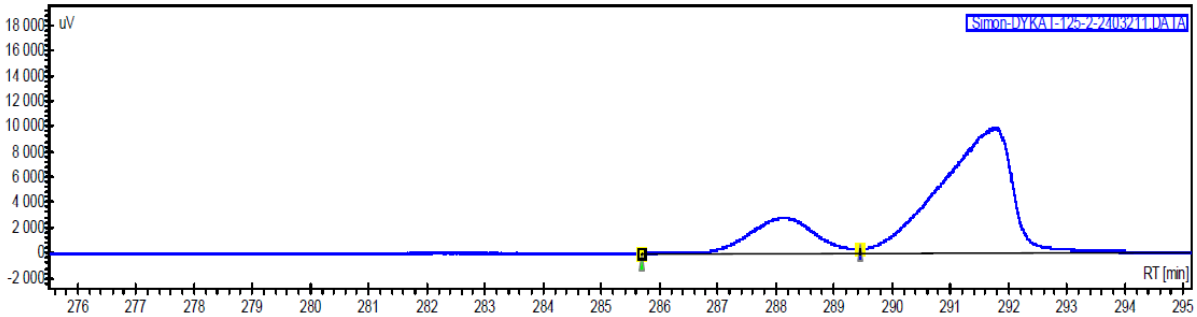

,

Simon-DYKAT-125-2-2403211.DATA [FID]

Index	Time [Min]	Quantity [% Area]	Height [uV]	Area [uV.Min]	Area % [%]
1	288.14	20.19	2816.6	3648.0	20.193
2	291.78	79.81	9971.7	14418.0	79.807
Total		100.00	12788.3	18066.0	100.000

*3-Benzyl-3-methyl-4-oxopentan-2-yl acetate (**2l**)*

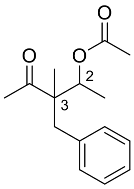

2l

Conditions A: Following the general procedure D (using 80 mg/mmol CALB and the reaction time was 90 h), β-oxoacetate **2l** (15 mg, 30%, *syn:anti* = 30:70, 98% *ee* (*syn*), 98% *ee* (*anti*)) was isolated as a colorless oil.

Mixture of *syn*- and *anti*-diastereomers: ^{1}H-NMR (400 MHz, $CDCl_3$): δ 7.31-7.13 (m, 3H), 7.11-6.95 (m, 2H), 5.22 (dq, *J* = 14.4, 6.4 Hz, 1H), 3.14 (d, *J* = 13.3 Hz, 1H), 3.04 (d, *J* = 13.2 Hz, 1H), 2.58 (d, *J* = 13.3 Hz, 1H), 2.08 (s, 3H), 2.01 (s, 3H), 2.00 (s, 3H), 1.99 (s, 3H), 1.26 (d, *J* = 6.4 Hz, 3H), 1.18 (d, *J* = 4.2 Hz, 3H), 1.16 (s, 3H); ^{13}C-NMR (100 MHz, $CDCl_3$): δ 217.2, 215.7, 137.6, 136.8, 130.4, 130.3, 128.4, 126.8, 126.7, 72.3, 71.7, 56.4, 56.0, 42.6, 40.9, 29.4, 29.3, 18.5, 17.7, 17.1; HRMS (ESI): calc. for $C_{15}H_{20}O_3Na$ $[M+Na]^+$: 271.1305; found: 271.1311.

GC: IVADEX-I, 60-0.5°C/min-100-0.1°C/min-120-20.0°C/min-200, t_R: 262.0 min for (2*S*,3*R*)-diastereomer, 270.1 min for (2*R*,3*S*)-diastereomer, 276.3 min for (2*S*,3*S*)-diastereomer and 281.6 min for (2*R*,3*R*)-diastereomer.

Racemate:

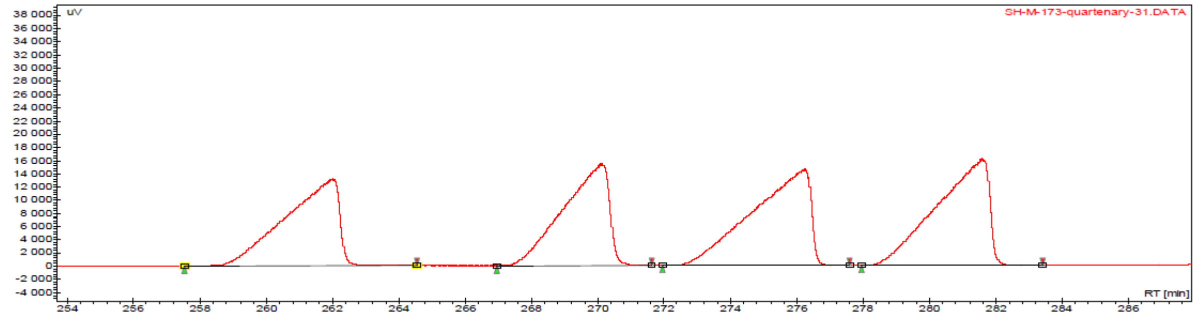

SH-M-173-quartenary-31.DATA [FID]

Index	Time [Min]	Quantity [% Area]	Height [uV]	Area [uV.Min]	Area % [%]
1	261.99	22.76	13248.1	24602.0	22.756
2	270.14	22.74	15481.1	24587.3	22.743
3	276.27	27.25	14633.5	29457.8	27.248
4	281.60	27.25	16178.3	29464.2	27.254
Total		100.00	59541.0	108111.2	100.000

Enantiomer (Reaction conditions A):

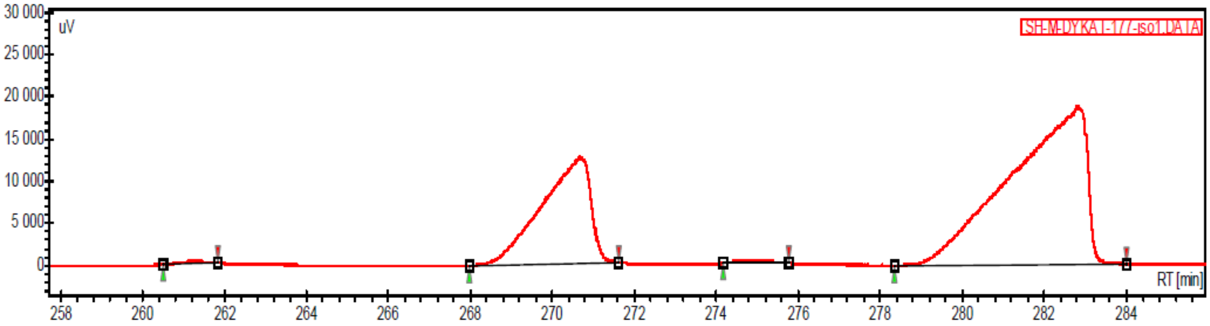

SH-M-DYKAT-177-iso1.DATA [FID]

Index	Time [Min]	Quantity [% Area]	Height [uV]	Area [uV.Min]	Area % [%]
4	261.20	0.36	264.1	207.1	0.359
2	270.69	30.09	12629.4	17339.8	30.092
3	274.83	0.54	340.6	313.0	0.543
1	282.84	69.01	18692.5	39762.6	69.005
Total		100.00	31926.5	57622.5	100.000

*2-Butyl-3-oxocyclohexyl acetate (**2m**)*

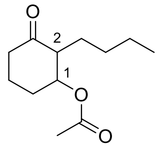

2m

Conditions A: Following the general procedure D (using 80 mg/mmol CALB and the reaction time was 90 h), β-oxoacetate **2m** (33 mg, 75%, *syn:anti* = 40:60, 99% *ee* (*syn*), 98% *ee* (*anti*)) was isolated as a colorless oil.

Syn-diastereomer: ^{1}H-NMR (400 MHz, $CDCl_3$): δ 5.41-5.46 (m, 1H), 2.38-2.50 (m, 2H), 2.24-2.34 (m, 1H), 2.02 (s, 3H), 1.90-2.15 (m, 2H), 1.69-1.86 (m, 2H), 1.42-1.69 (m, 2H), 1.18-1.35 (m, 4H), 0.88 (t, J = 6.9 Hz, 3H). ^{13}C-NMR (101 MHz, $CDCl_3$): δ 210.8, 170.3, 74.9, 53.8, 41.5, 31.1, 29.5, 29.3, 24.8, 22.9, 21.9, 14.1. *Anti*-diastereomer: ^{1}H-NMR (400 MHz, $CDCl_3$): δ 4.97 (td, J = 6.9, 3.5 Hz, 1H), 2.38-2.50 (m, 2H), 2.24-2.34 (m, 1H), 2.04 (s, 3H), 1.90-2.15 (m, 2H), 1.69-1.86 (m, 2H), 1.42-1.69 (m, 2H), 1.18-1.35 (m, 4H), 0.88 (t, J = 6.9 Hz, 3H). ^{13}C-NMR (101 MHz, $CDCl_3$): δ 210.8, 170.3, 75.5, 55.7, 39.5, 29.6, 27.8, 27.7, 22.8, 21.3, 21.0, 14.0; HRMS (ESI): calc. for $C_{12}H_{20}O_3Na$ $[M+Na]^+$: 235.1305; found: 235.1300.

GC: Hydrodex β-DM, 50-1.0°C/min-170, t_R: 63.2 min for (1*S*,2*R*)-diastereomer, 63.7 min (1*R*,2*S*)-diastereomer, 64.4 min for (1*S*,2*S*)-diastereomer and 68.8 min for (1*R*,2*R*)-diastereomer

Racemate:

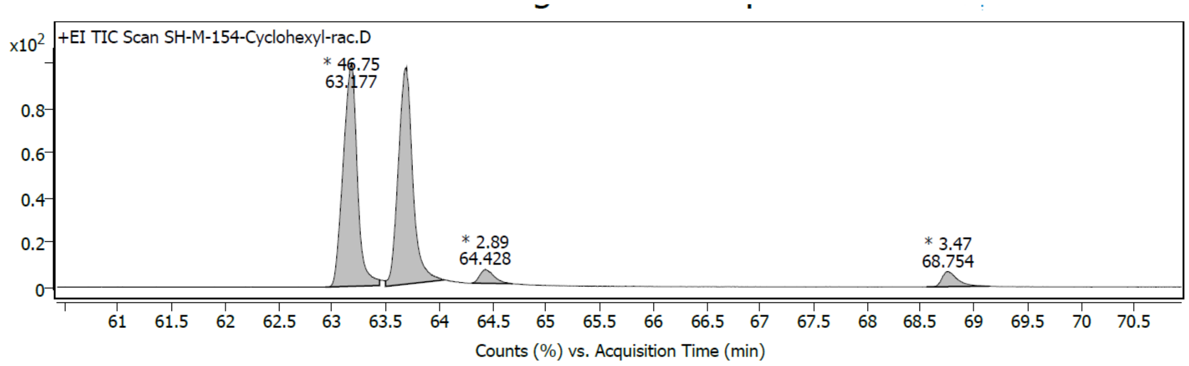

Enantiomer (Reaction conditions A):

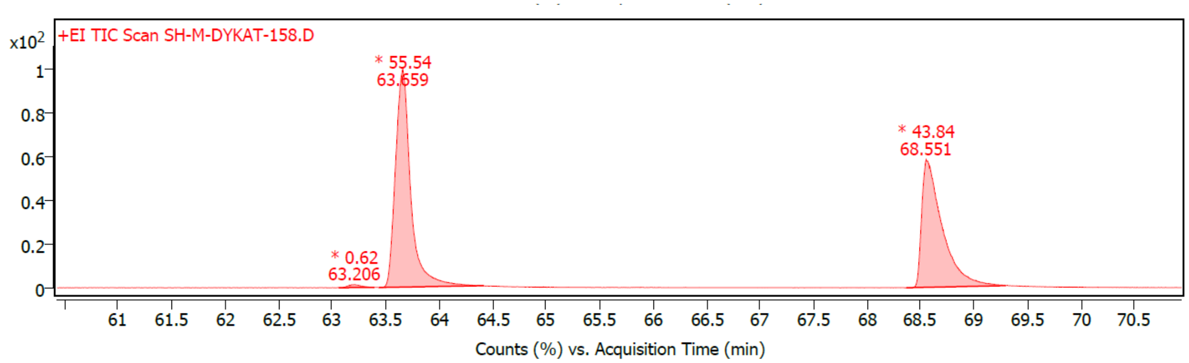

References

[1] a) J. Paetzold, J.-E. Bäckvall, *J. Am. Chem. Soc.* **2005**, *127*, 17620. b) B. Martín-Matute, M. Edin, K. Bogár, F. B. Kaynak, J.-E. Bäckvall, *J. Am. Chem. Soc.* **2005**, *127*, 8817.

[2] a) D. Kalaitzakis, I. Smonou, *J. Org. Chem.* **2008**, *73*, 3919. b) C. H. Heathcock, M. C. Pirrung, J. E. Sohn, J. Org. Chem. **1979**, *44*, 4294.

[3] T. Hayashi, Y. Matsumoto, Y. Ito, *J. Am. Chem. Soc.* **1988**, *110*, 5579.

[4] D. Kalaitzakis, J. D. Rozzell, I. Smonou, S. Kambourakis, *Adv. Synth. Catal.* **2006**, *348*, 1958.

[5] C. Körner, P. Starkov, T. D. Sheppard, *J. Am. Chem. Soc.* **2010**, *132*, 5968.

[6] D. Kalaitzakis, J. D. Rozzell, S. Kambourakis, I. Smonou, *Eur. J. Org. Chem.* **2006**, *2006*, 2309.

[7] D. Tanner, M. Sellen, J.-E. Bäckvall, *J. Org. Chem.* **1989**, *54*, 3374.

^{1}H- and ^{13}C-NMR spectra of compounds

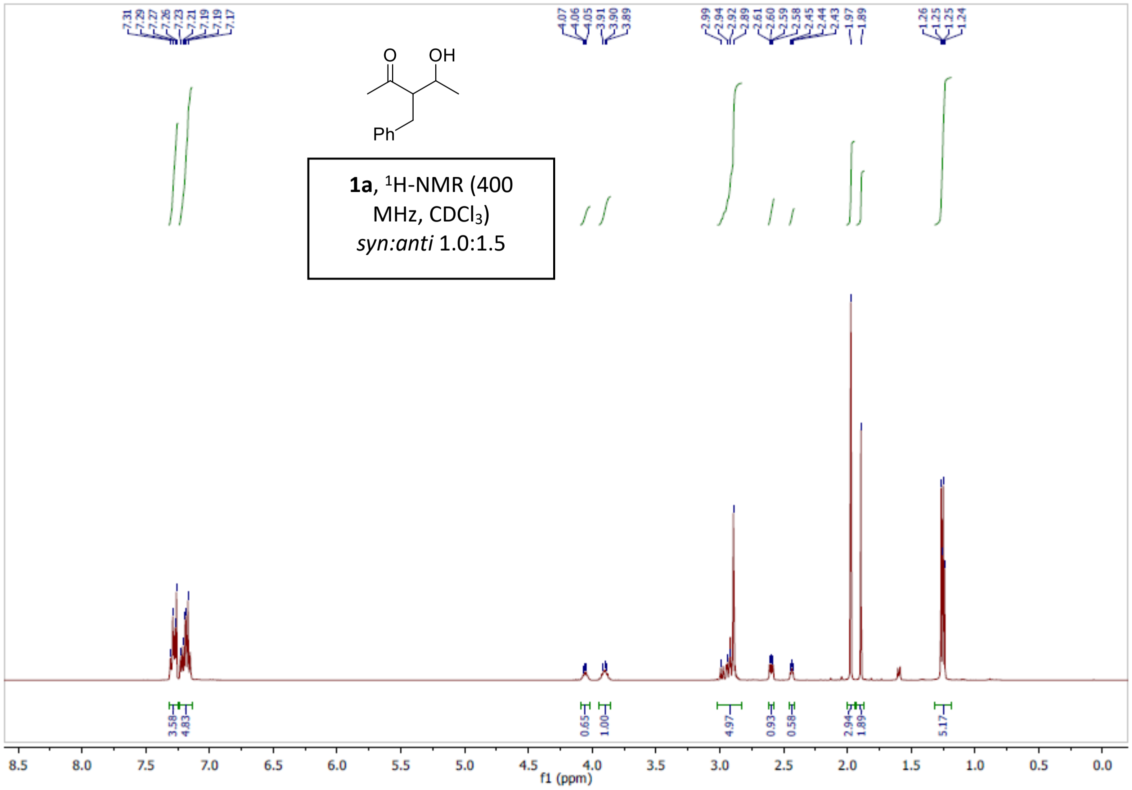

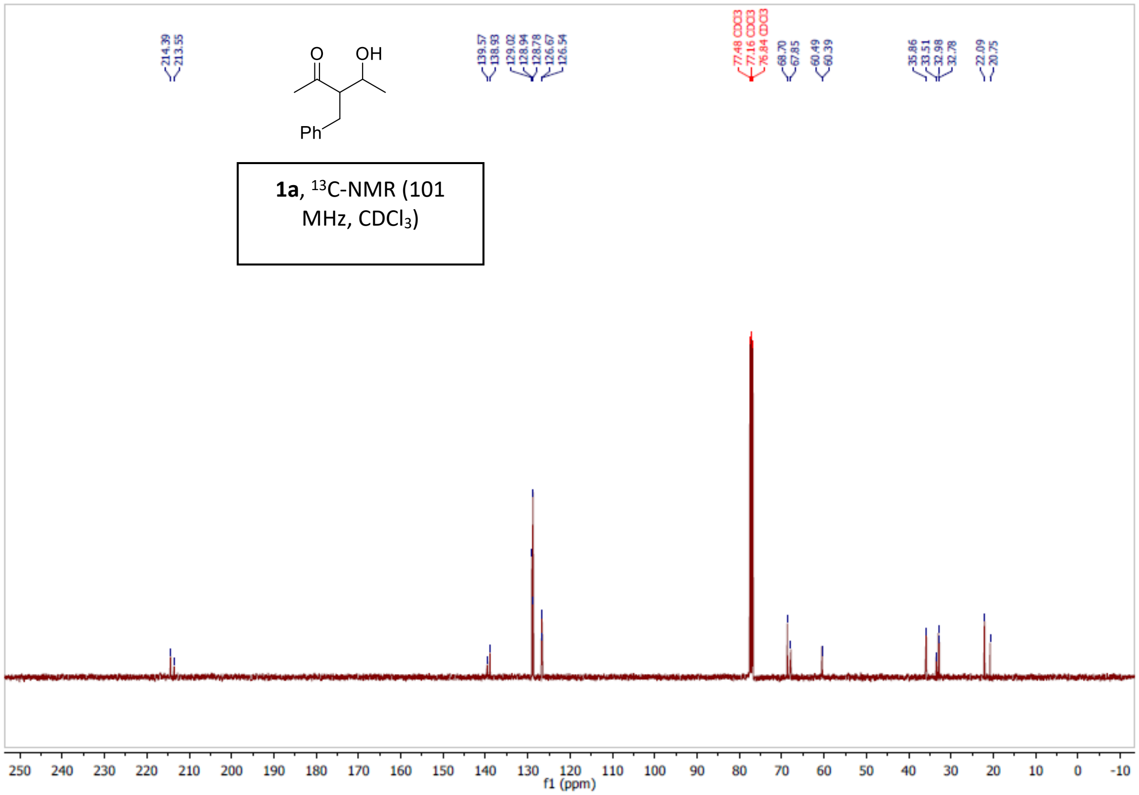

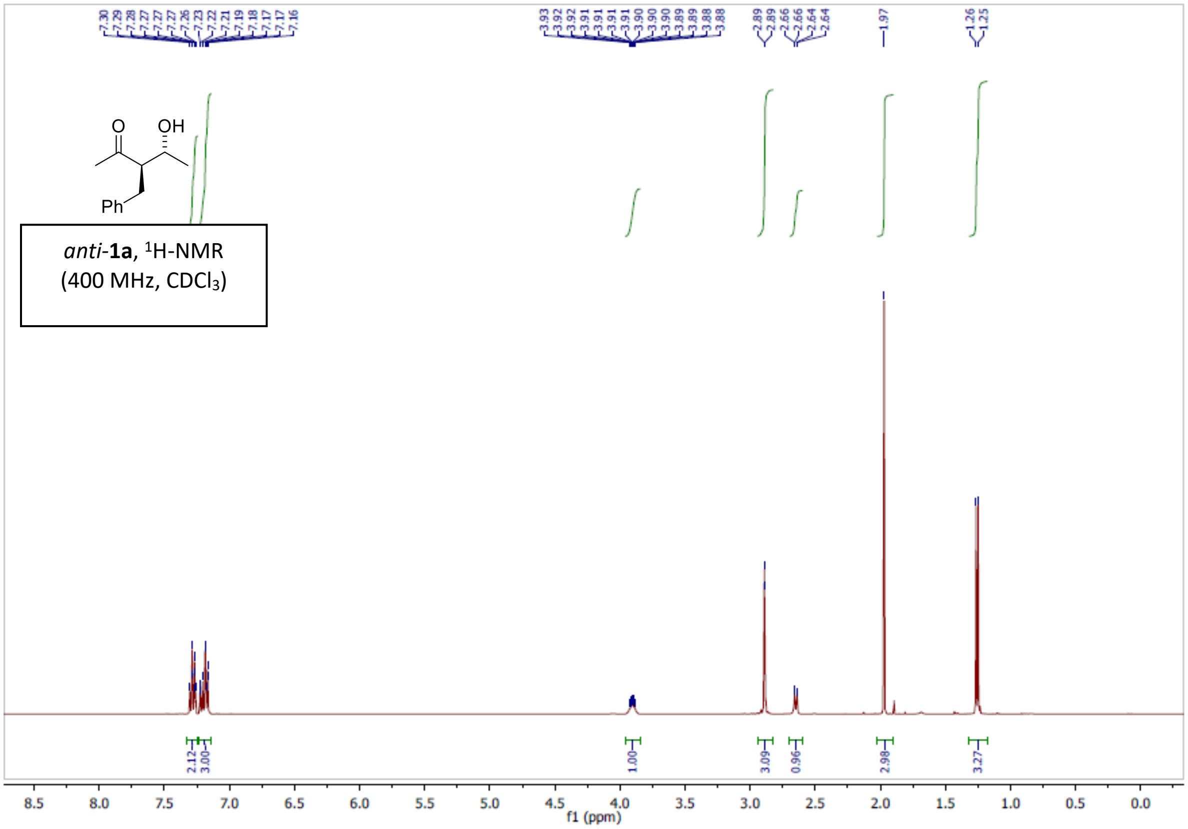
O
OH
Ph
anti-1a, 1H-NMR
(400 MHz, CDCl3)
f1 (ppm)

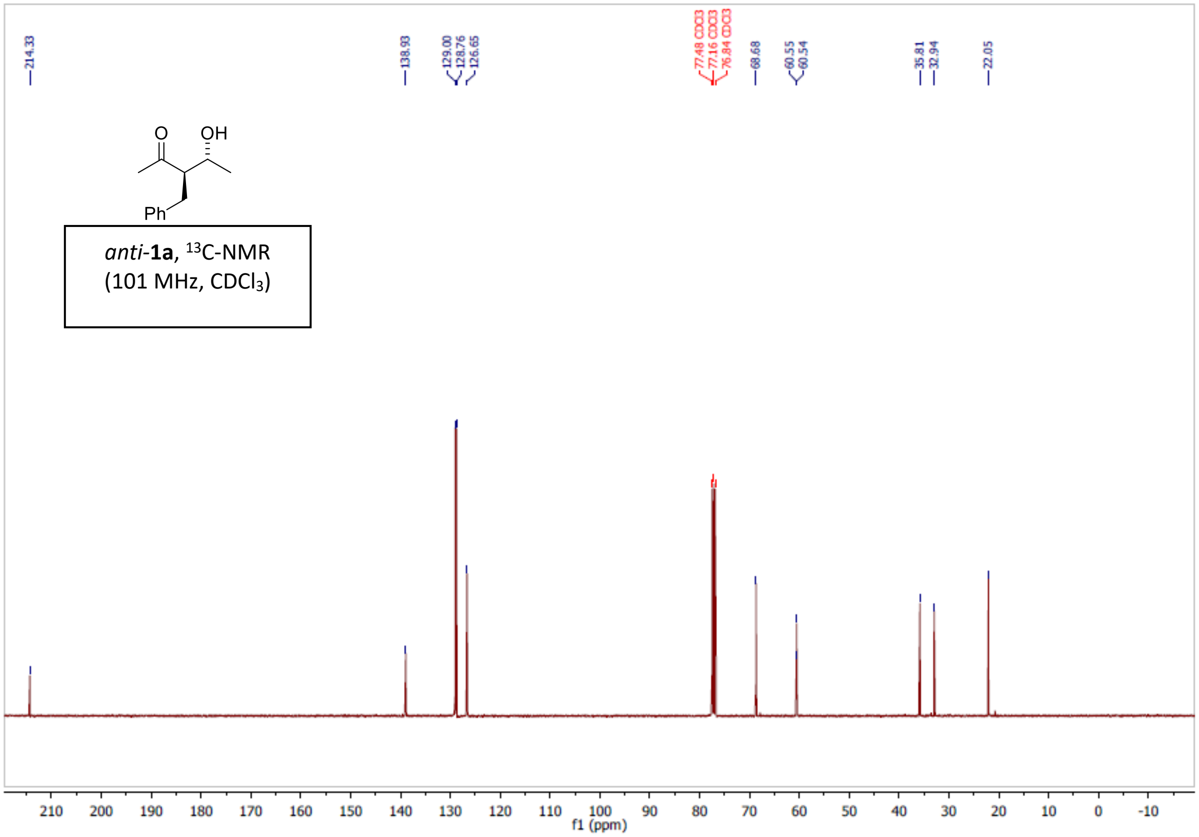
O
OH
Ph
anti-1a, 13C-NMR
(101 MHz, CDCl3)
f1 (ppm)

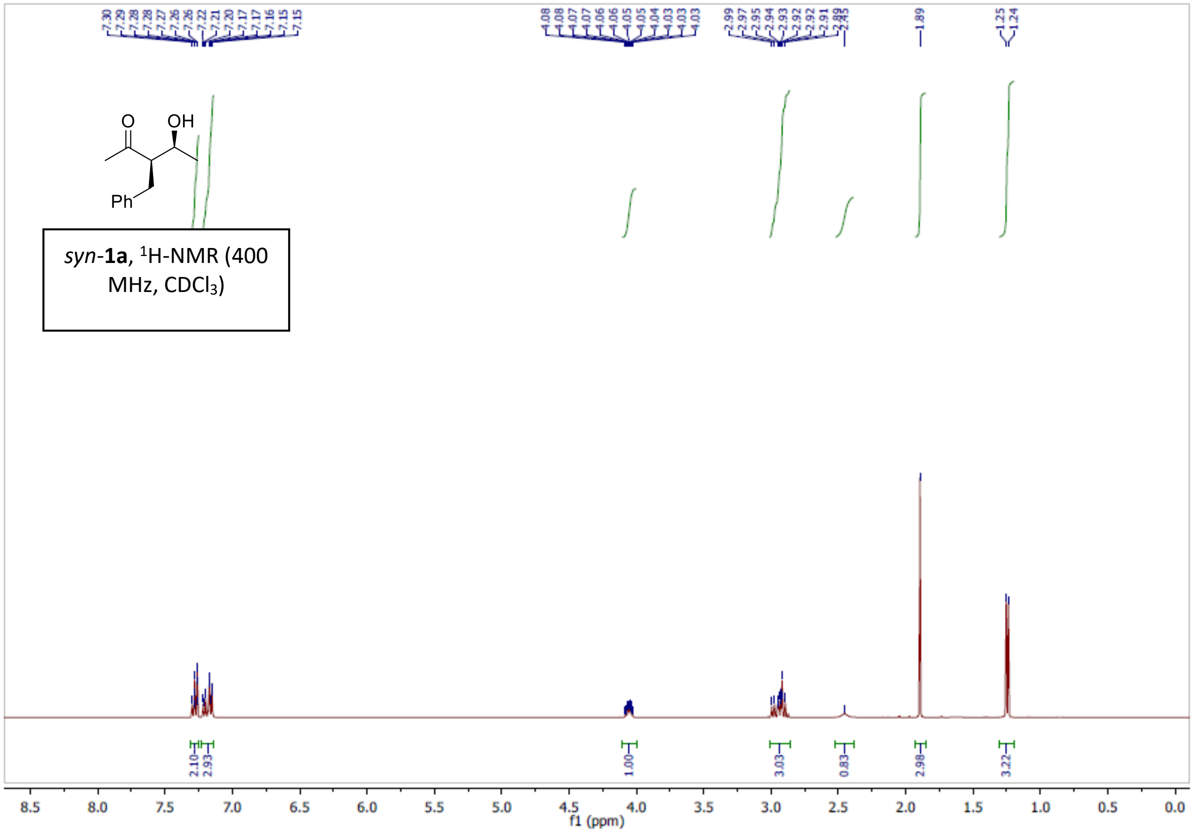

syn-**1a**, ^{1}H-NMR (400 MHz, $CDCl_3$)

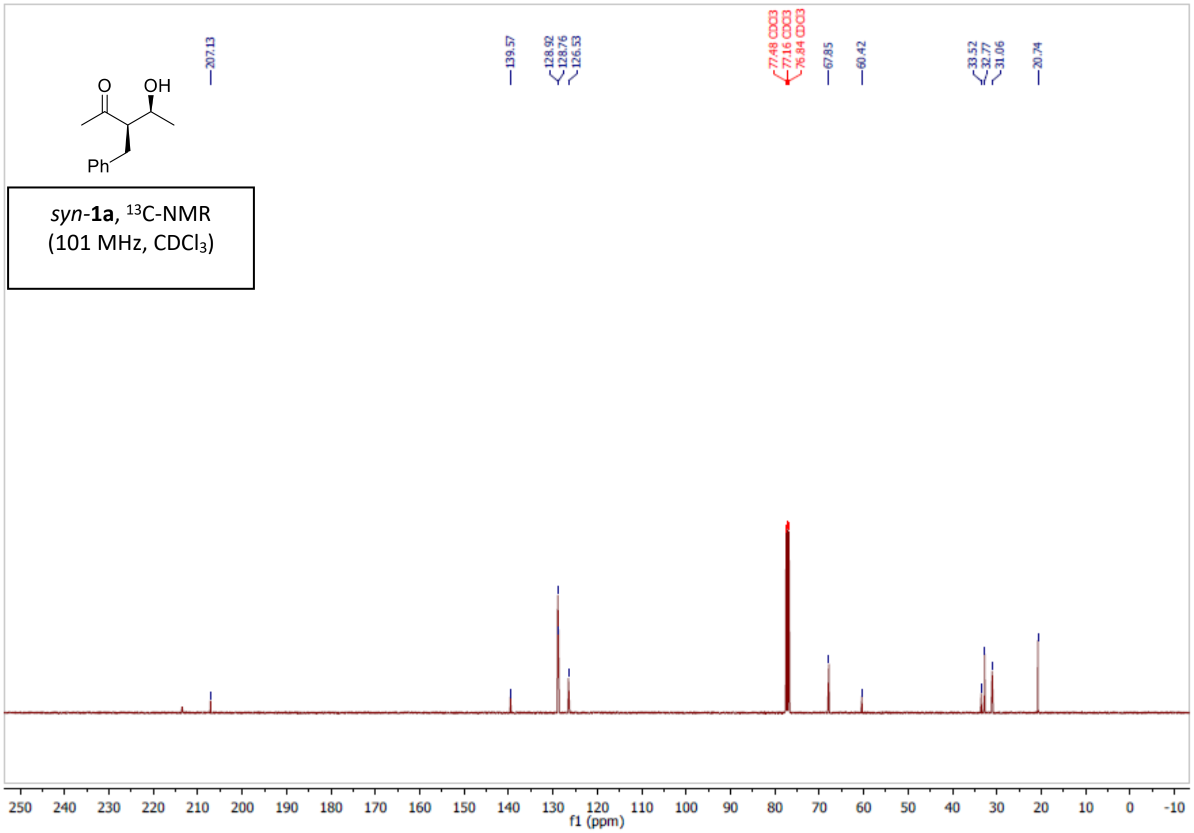

syn-**1a**, ^{13}C-NMR (101 MHz, $CDCl_3$)

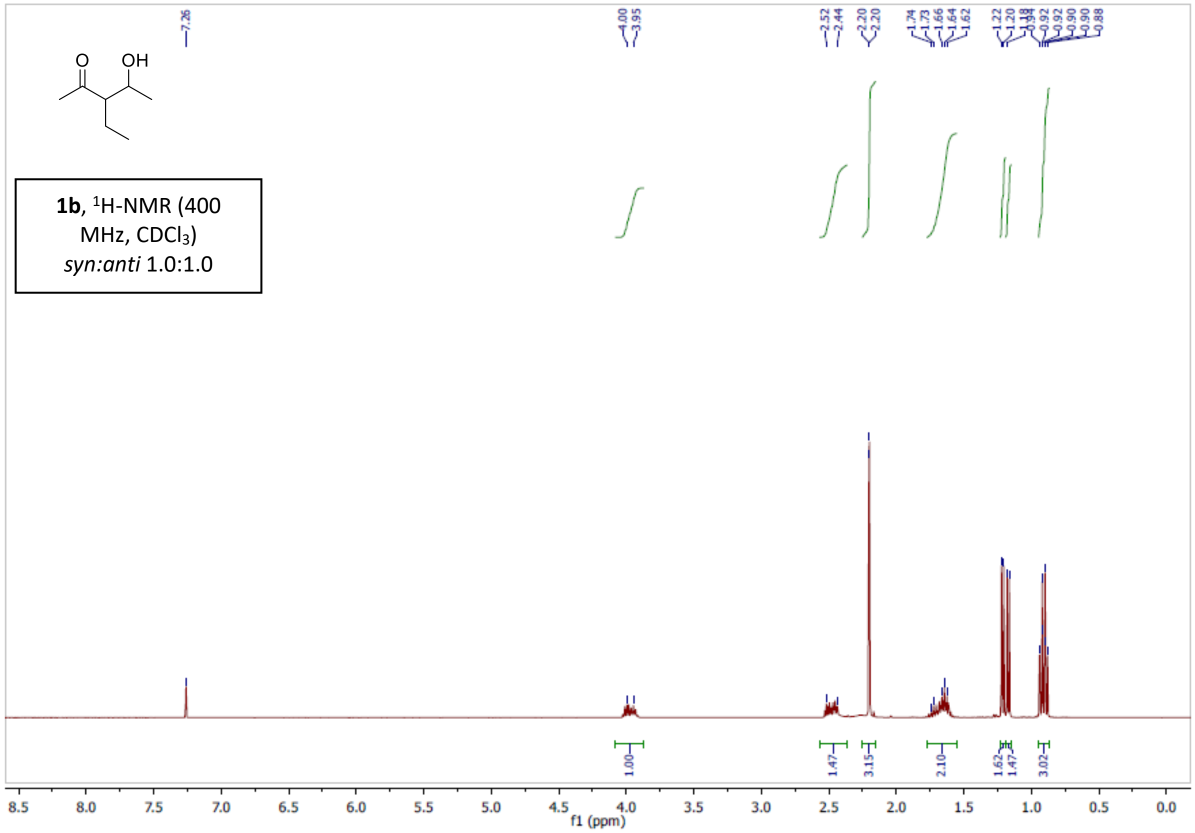

O
OH
1b, ^{1}H-NMR (400 MHz, $CDCl_3$)
syn:anti 1.0:1.0
7.26
4.00
3.95
2.52
2.44
2.20
2.20
1.74
1.73
1.66
1.64
1.62
1.22
1.20
1.18
0.94
0.92
0.92
0.90
0.90
0.88
1.00
1.47
3.15
2.10
1.62
1.47
3.02
8.5 8.0 7.5 7.0 6.5 6.0 5.5 5.0 4.5 4.0 3.5 3.0 2.5 2.0 1.5 1.0 0.5 0.0
f1 (ppm)

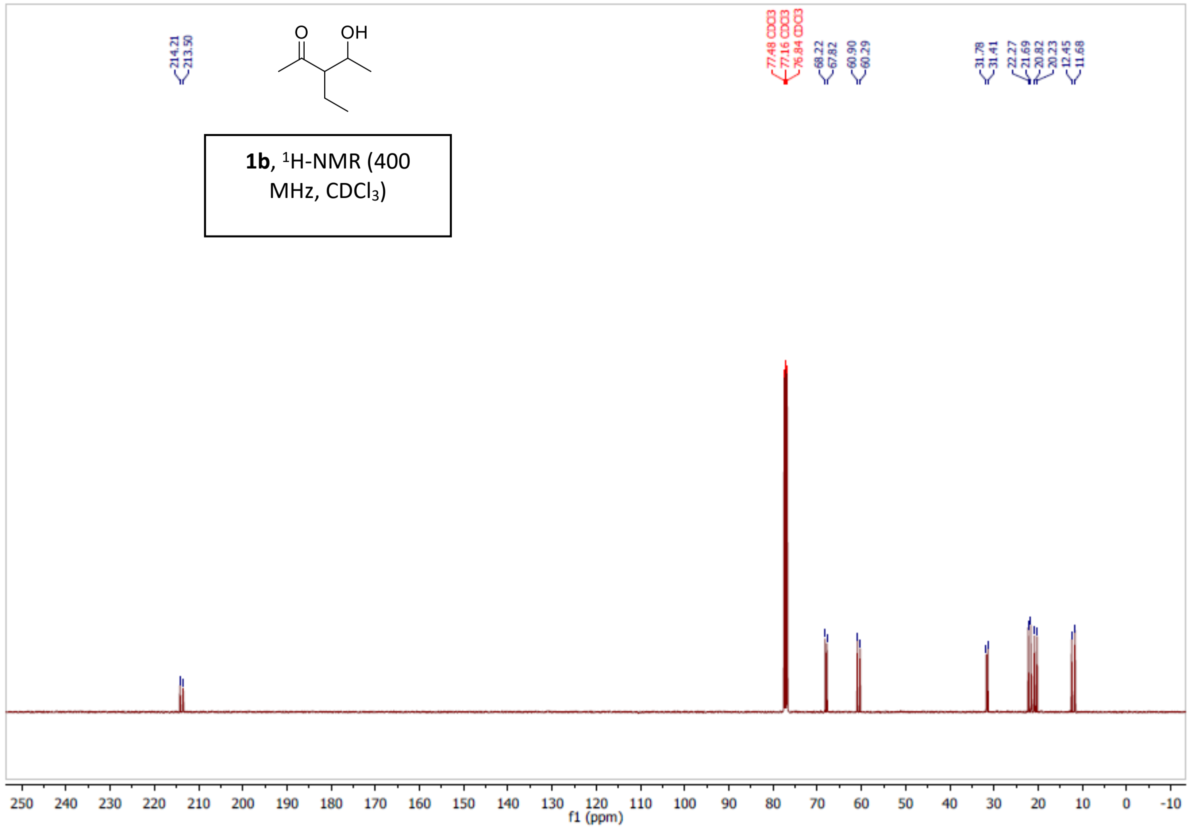

O
OH
1b, ^{1}H-NMR (400 MHz, $CDCl_3$)
214.21
213.50
77.48 CDCl3
77.16 CDCl3
76.84 CDCl3
68.22
67.82
60.90
60.29
31.78
31.41
22.27
21.69
20.82
20.23
12.45
11.68
250 240 230 220 210 200 190 180 170 160 150 140 130 120 110 100 90 80 70 60 50 40 30 20 10 0 -10
f1 (ppm)

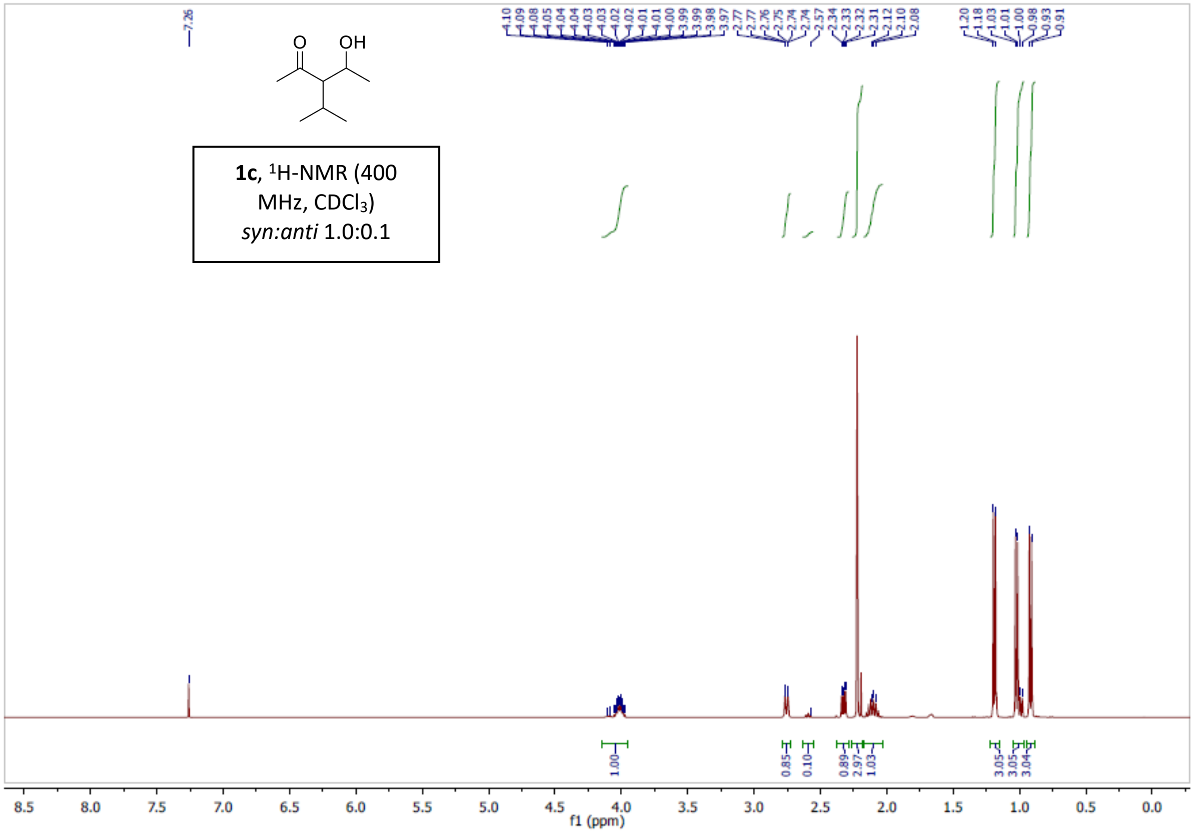
1c, 1H-NMR (400 MHz, CDCl3)
syn:anti 1.0:0.1
f1 (ppm)

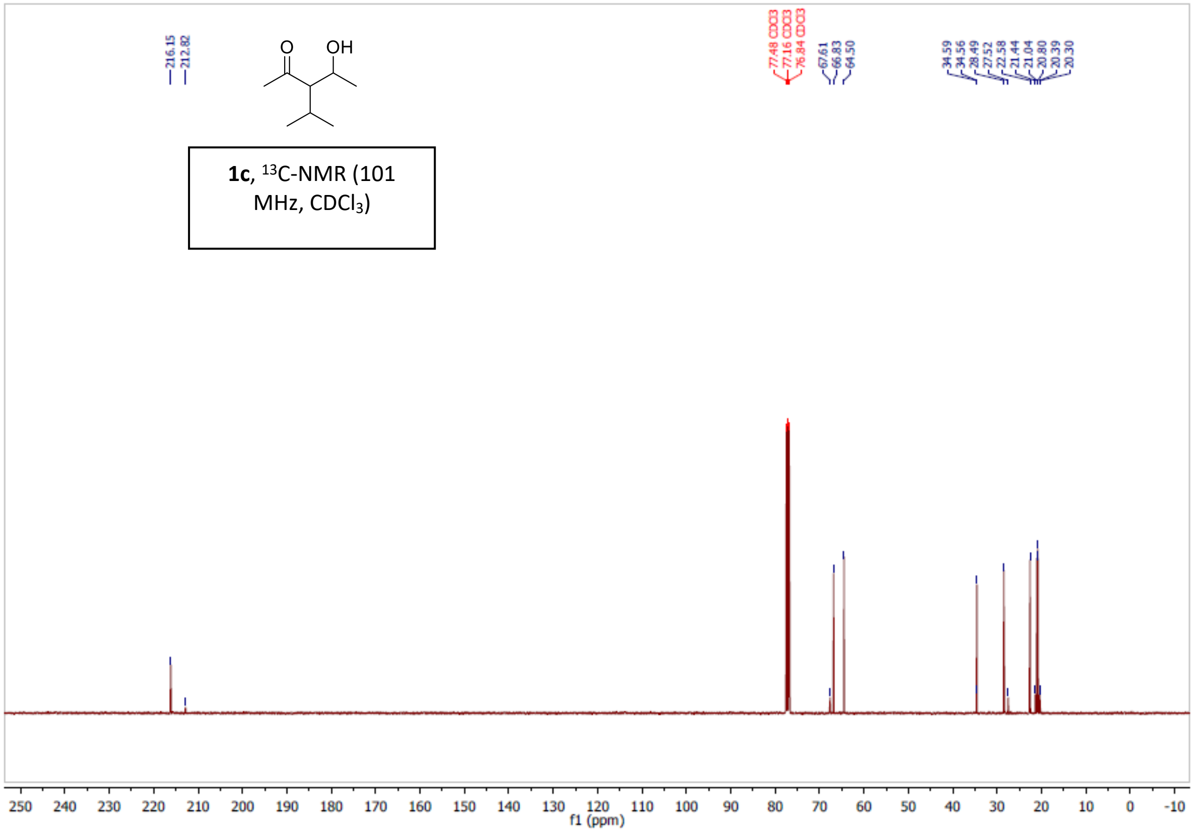
1c, 13C-NMR (101 MHz, CDCl3)
f1 (ppm)

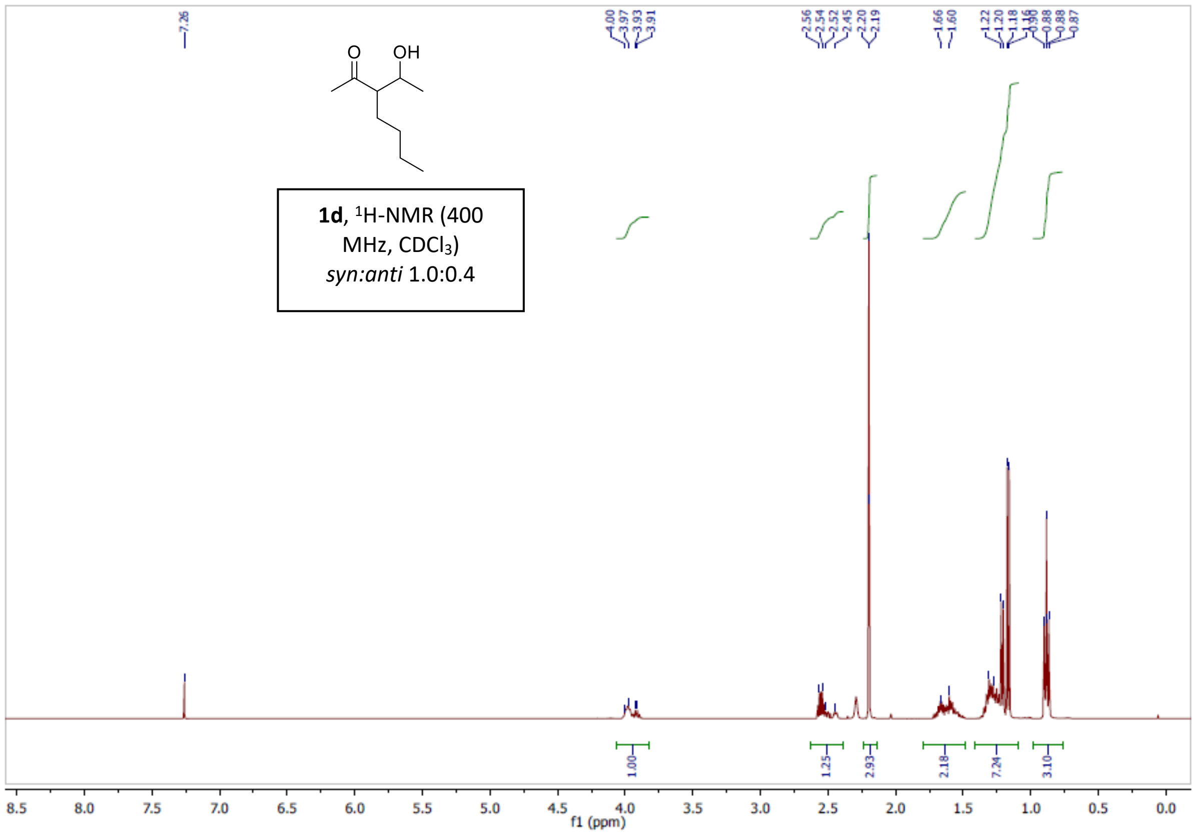
1d, ^{1}H-NMR (400 MHz, CDCl$_3$)
syn:anti 1.0:0.4
OH
O
f1 (ppm)

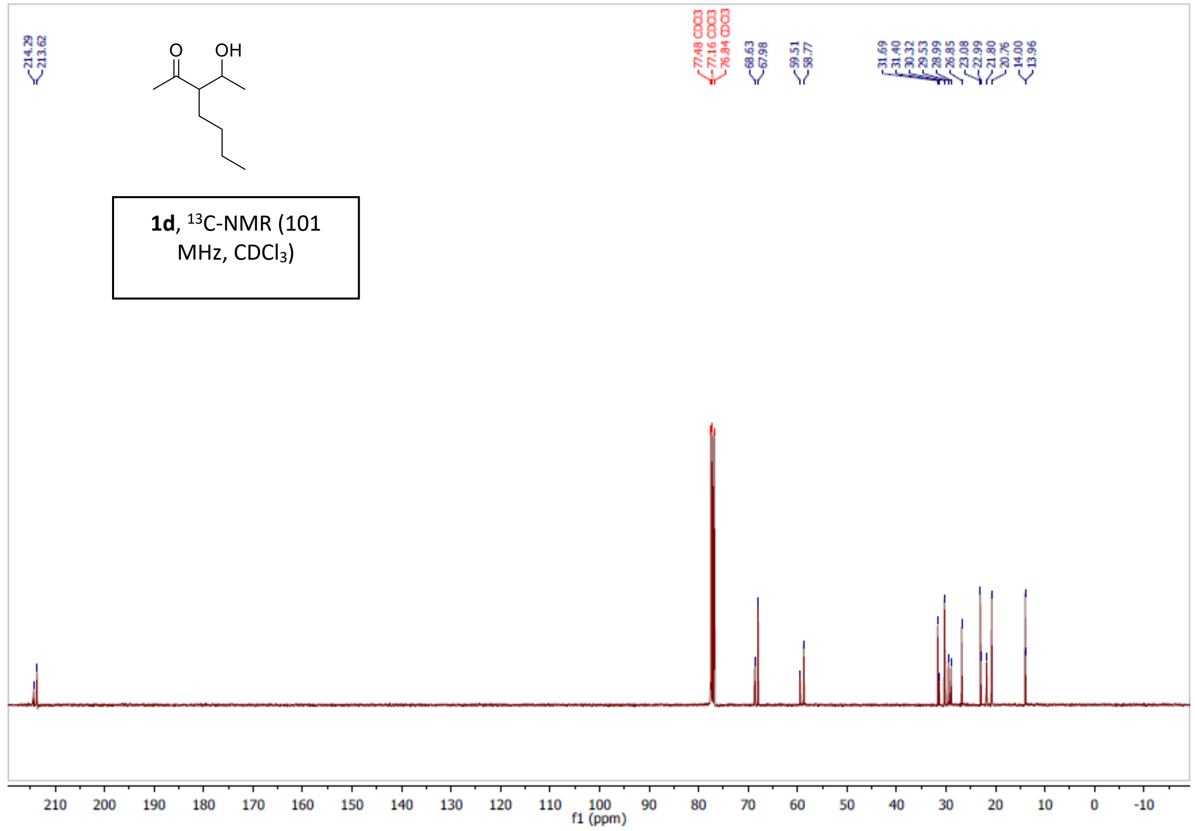
1d, ^{13}C-NMR (101 MHz, CDCl$_3$)
OH
O
f1 (ppm)

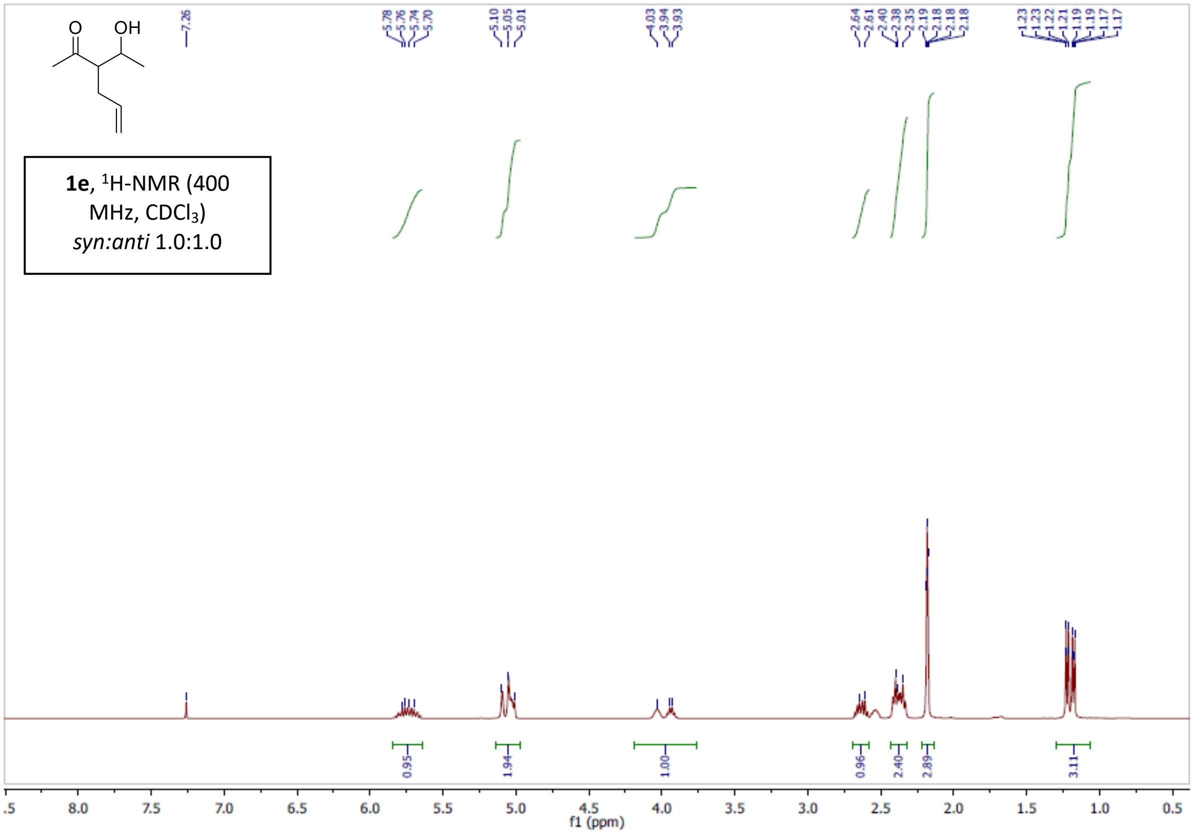
O
OH
1e, ¹H-NMR (400 MHz, CDCl3)
syn:anti 1.0:1.0
7.26
5.78 5.76 5.74 5.70
5.10 5.05 5.01
4.03 3.94 3.93
2.64 2.61 2.40 2.38 2.35 2.19 2.18 2.18 2.18
1.23 1.23 1.22 1.21 1.19 1.19 1.17 1.17
0.95
1.94
1.00
0.96
2.40
2.89
3.11
f1 (ppm)

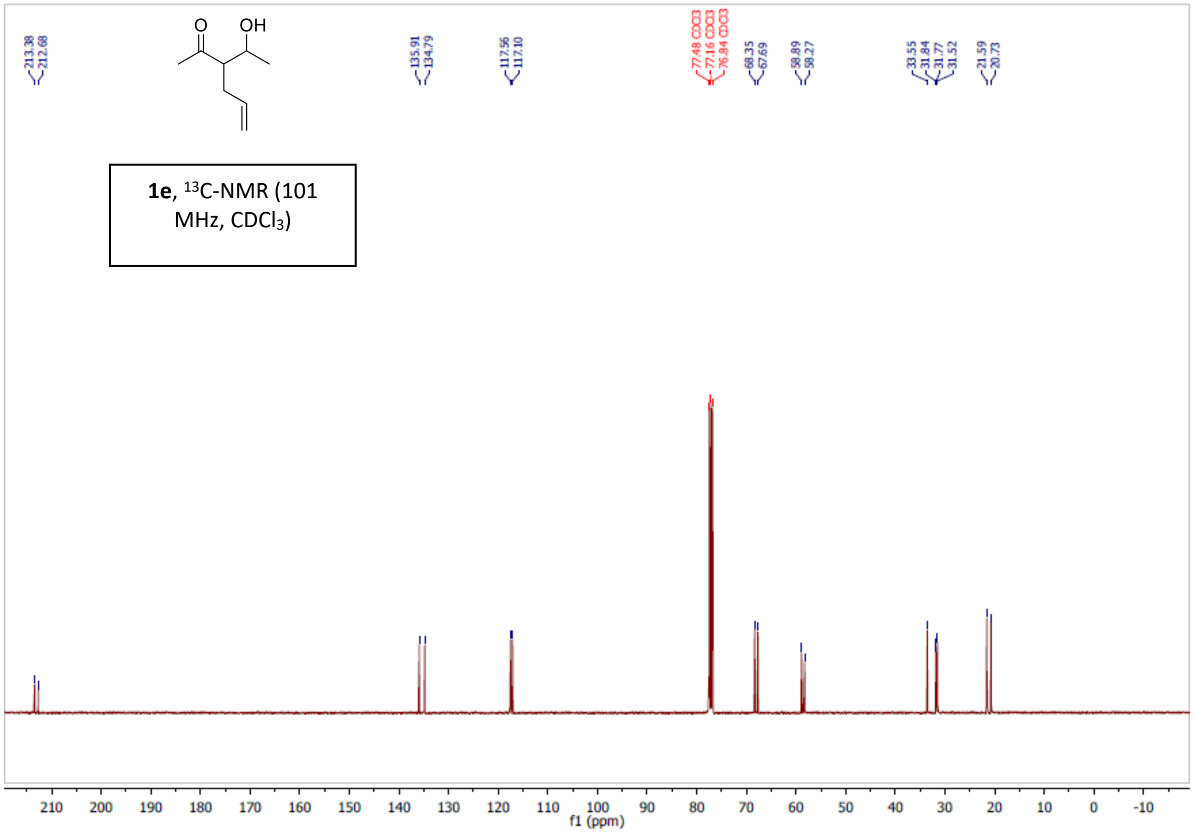
O
OH
1e, ¹³C-NMR (101 MHz, CDCl3)
213.38 212.68
135.91 134.79
117.56 117.10
77.48 CDCl3 77.16 CDCl3 76.84 CDCl3
68.35 67.69
58.89 58.27
33.55 31.84 31.77 31.52
21.59 20.73
f1 (ppm)

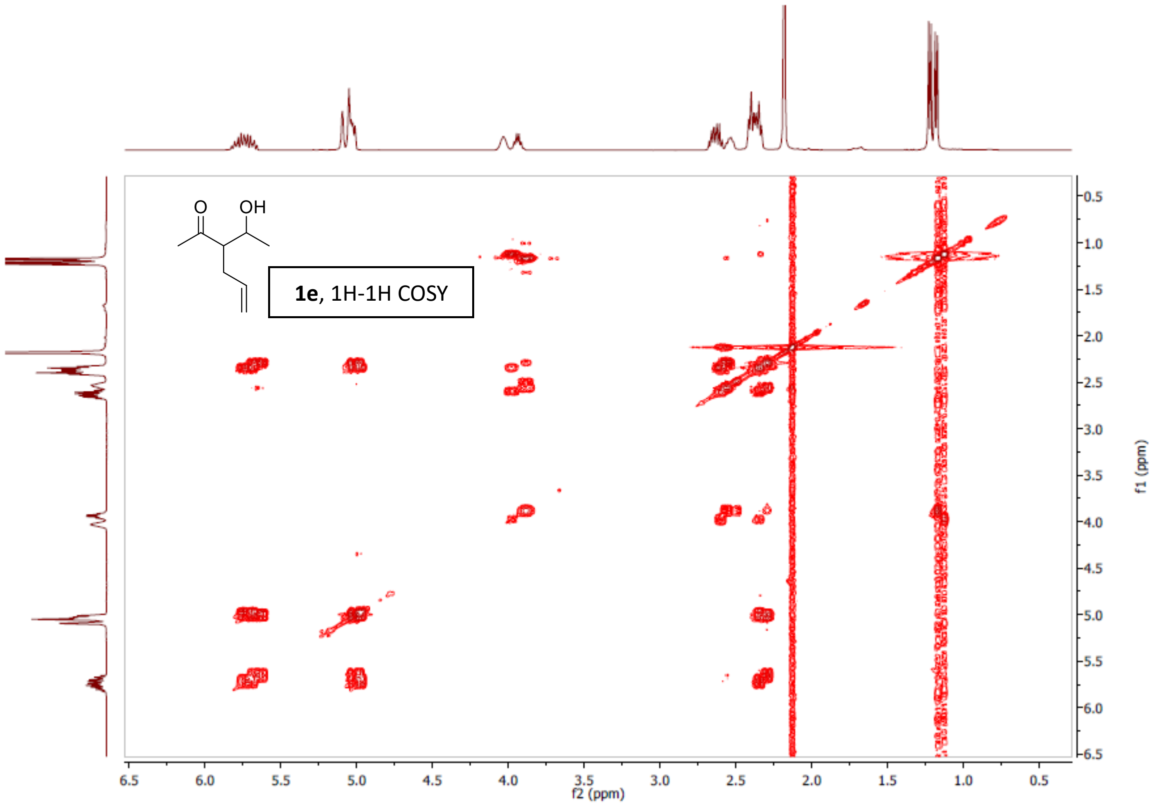

O
OH
1e, 1H-1H COSY
f2 (ppm)
f1 (ppm)

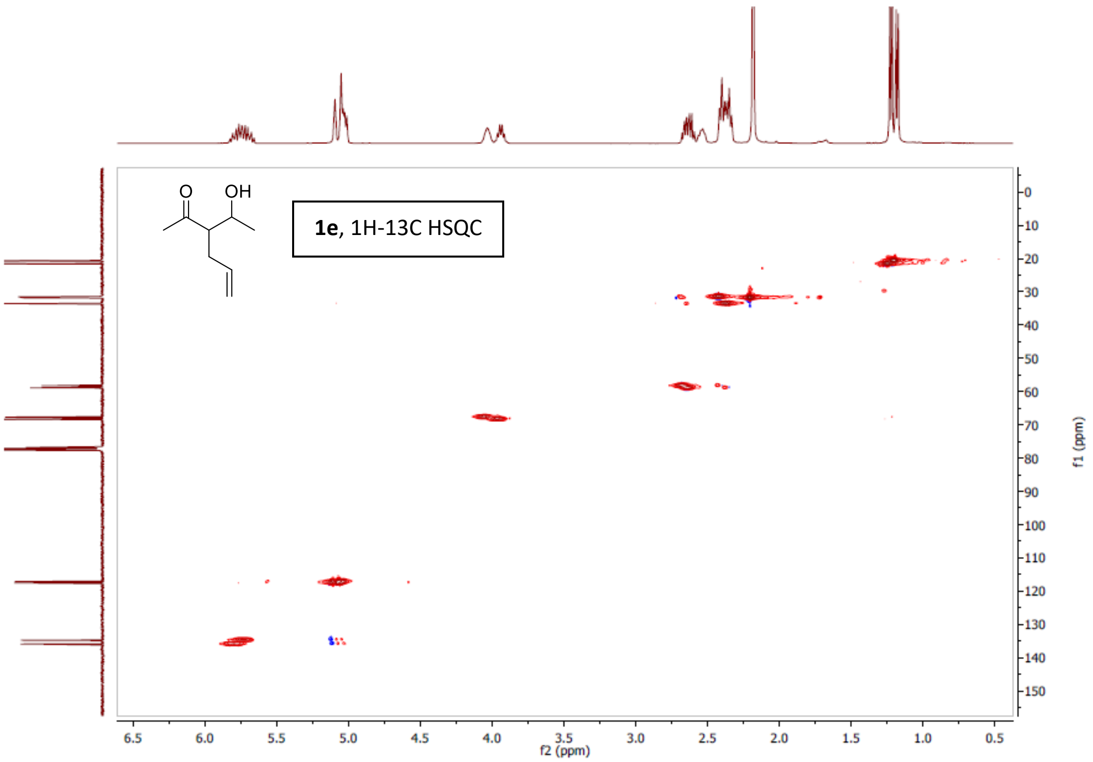

O
OH
1e, 1H-13C HSQC
f2 (ppm)
f1 (ppm)

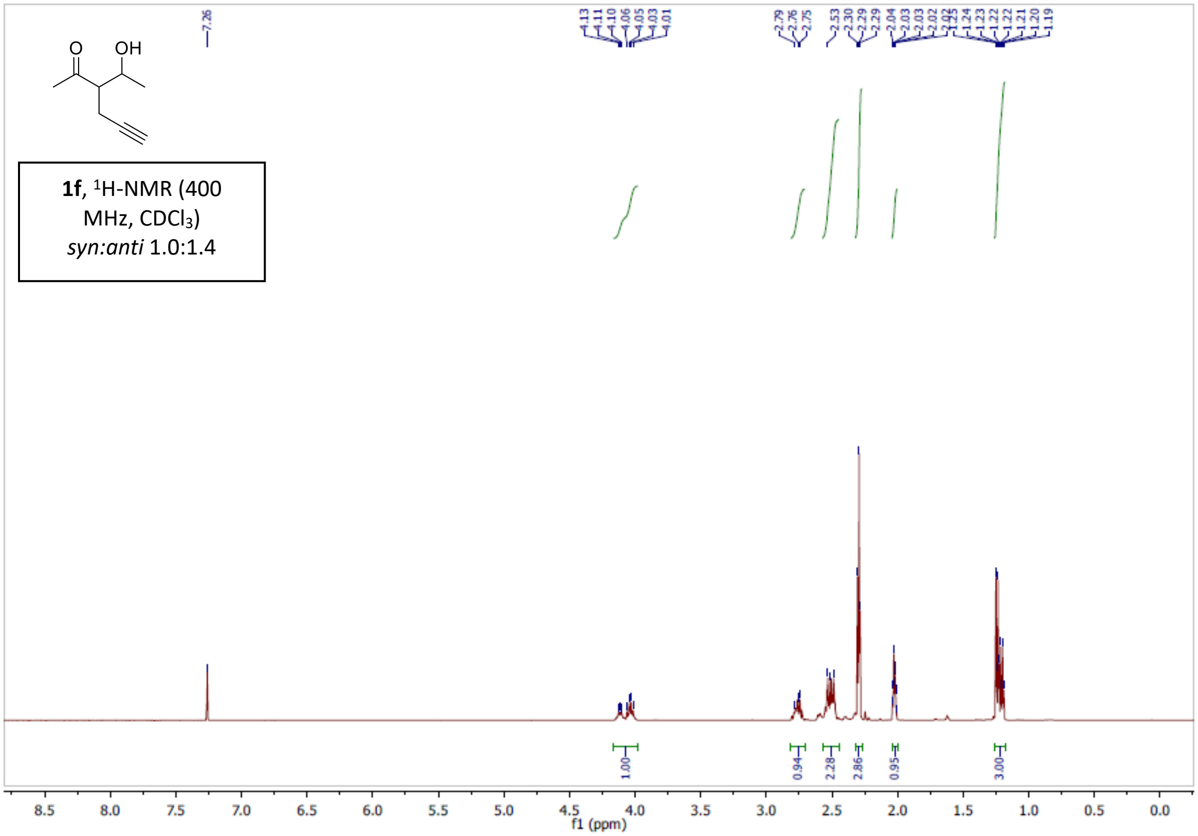
O
OH
1f, ^{1}H-NMR (400 MHz, $CDCl_3$)
syn:anti 1.0:1.4
7.26
4.13 4.11 4.10 4.06 4.05 4.03 4.01
2.79 2.76 2.75 2.53 2.30 2.29 2.29 2.04 2.03 2.03 2.02 2.02 1.25 1.24 1.23 1.22 1.22 1.21 1.20 1.19
1.00 0.94 2.28 2.86 0.95 3.00
f1 (ppm)

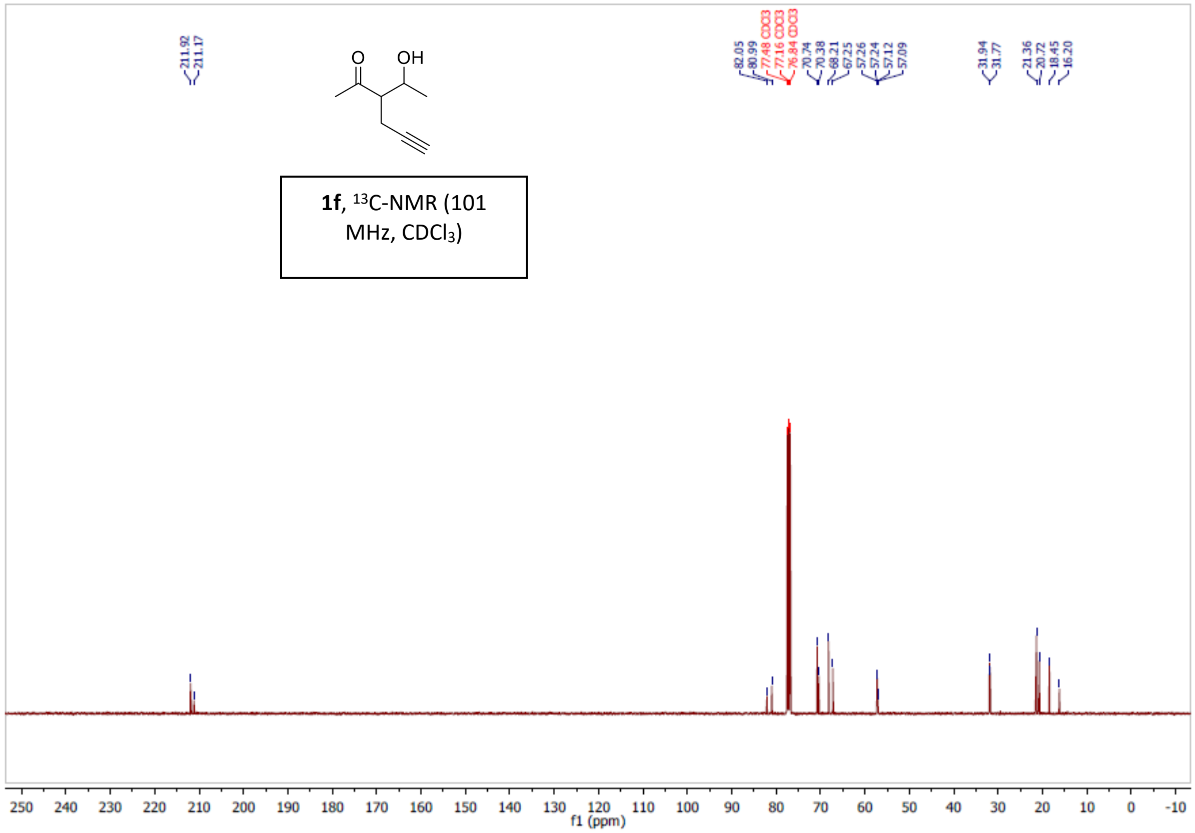
211.92 211.17
82.05 80.99 77.48 CDCl3 77.16 CDCl3 76.84 CDCl3 70.74 70.38 68.21 67.25 57.26 57.24 57.12 57.09
31.94 31.77 21.36 20.72 18.45 16.20
O
OH
1f, ^{13}C-NMR (101 MHz, $CDCl_3$)
f1 (ppm)

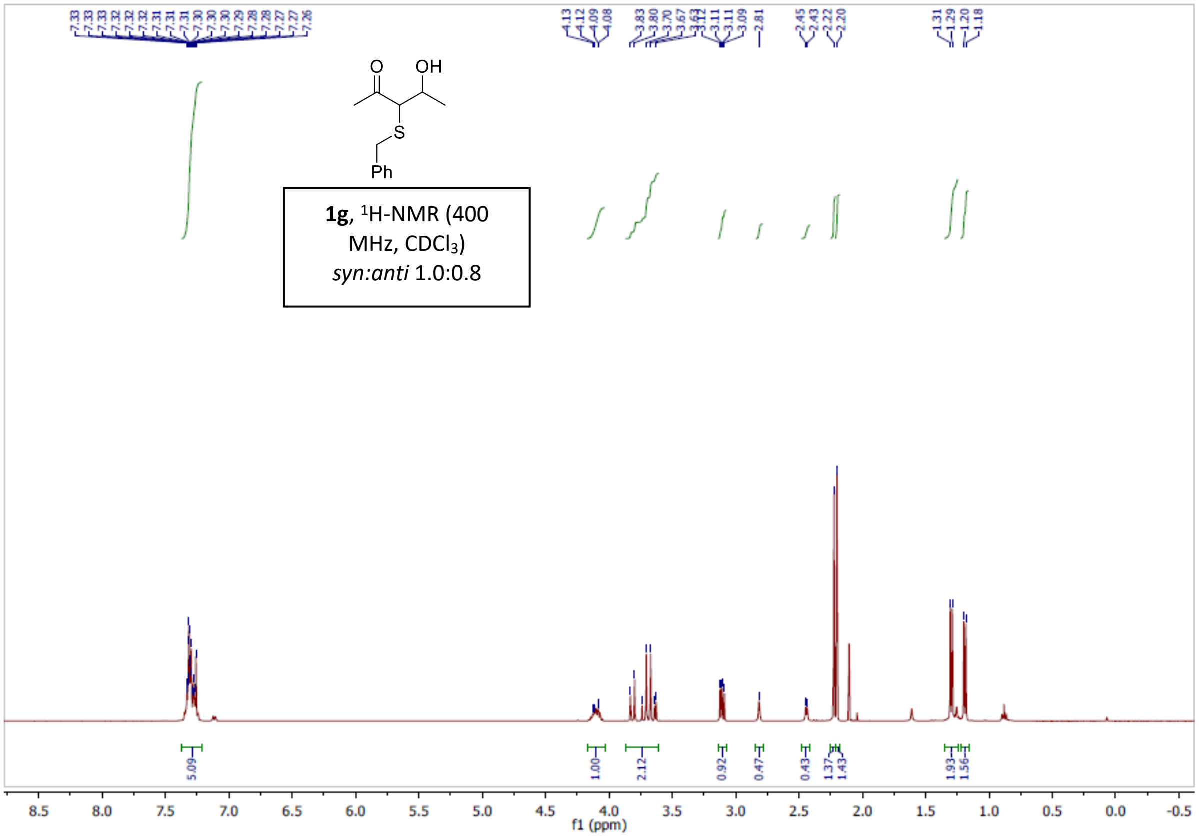
1g, ¹H-NMR (400 MHz, CDCl₃)
syn:anti 1.0:0.8
f1 (ppm)

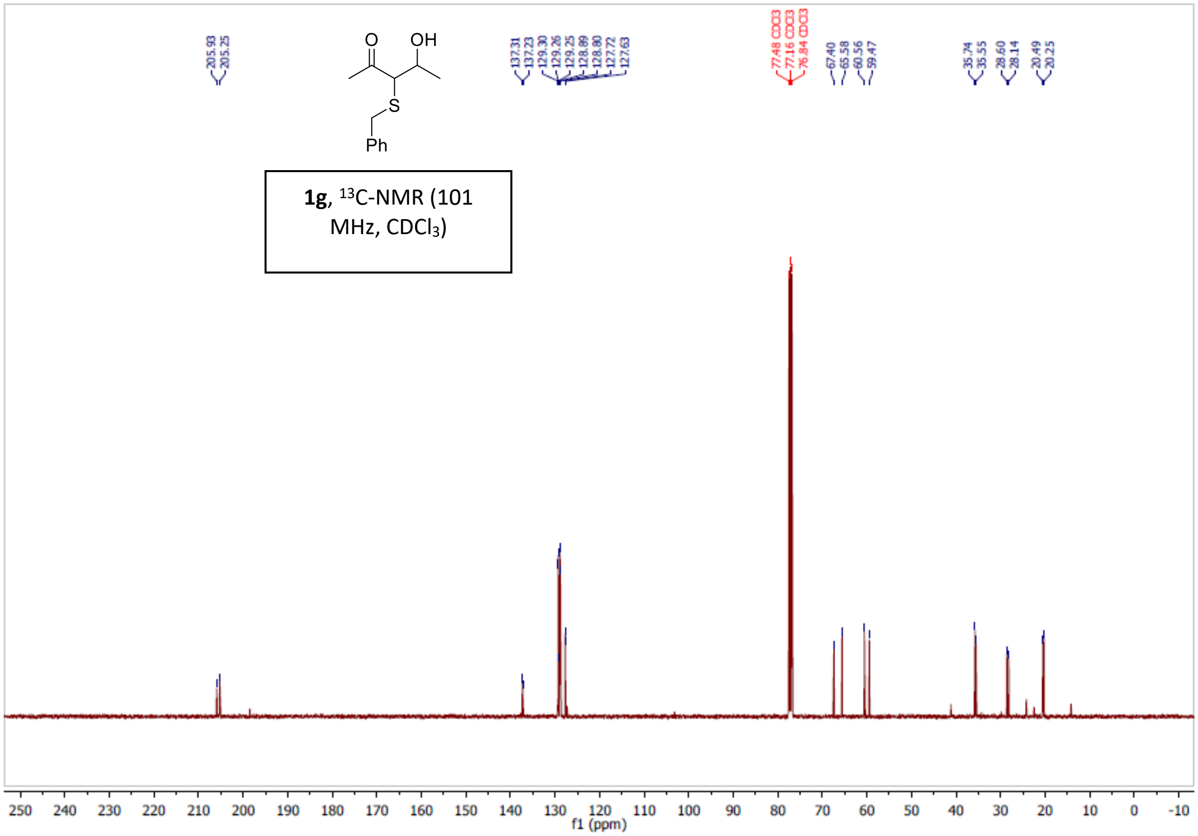
1g, ¹³C-NMR (101 MHz, CDCl₃)
f1 (ppm)

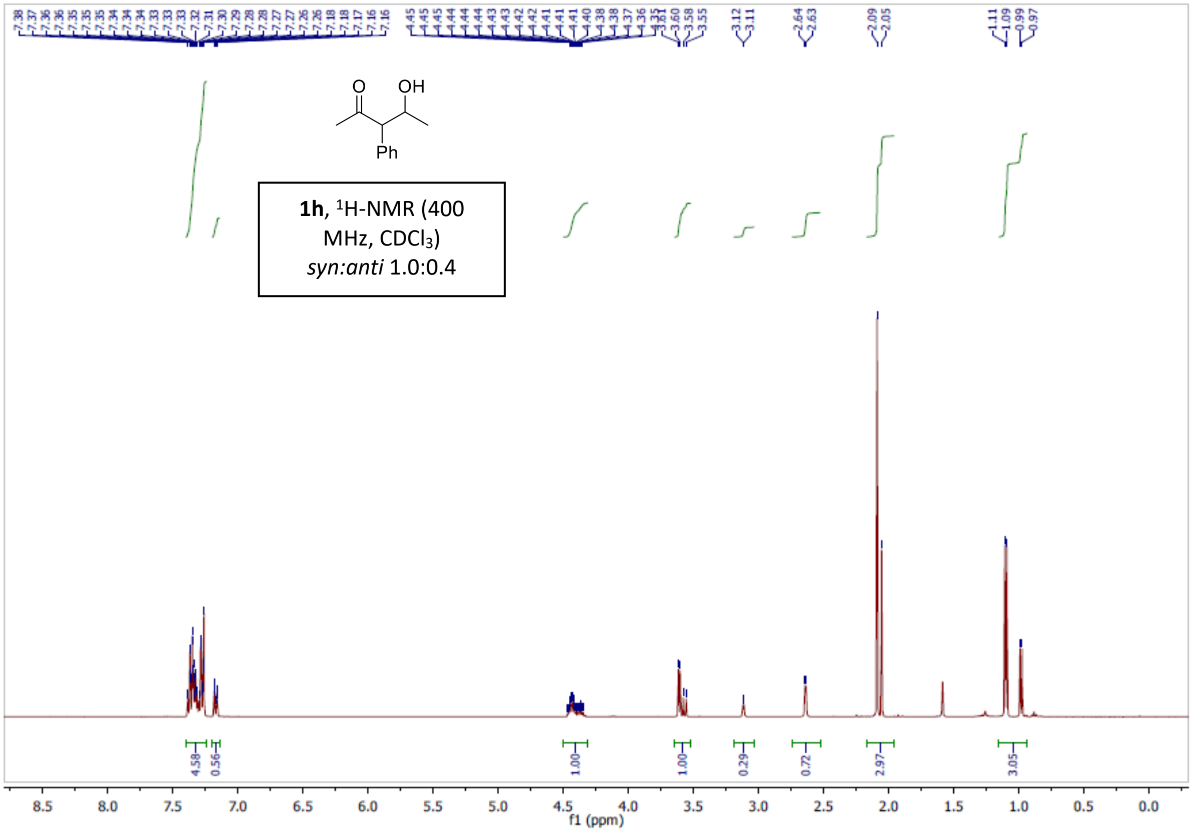
O
OH
Ph
1h, ^{1}H-NMR (400 MHz, $CDCl_3$)
syn:anti 1.0:0.4
4.58
0.56
1.00
1.00
0.29
0.72
2.97
3.05
f1 (ppm)

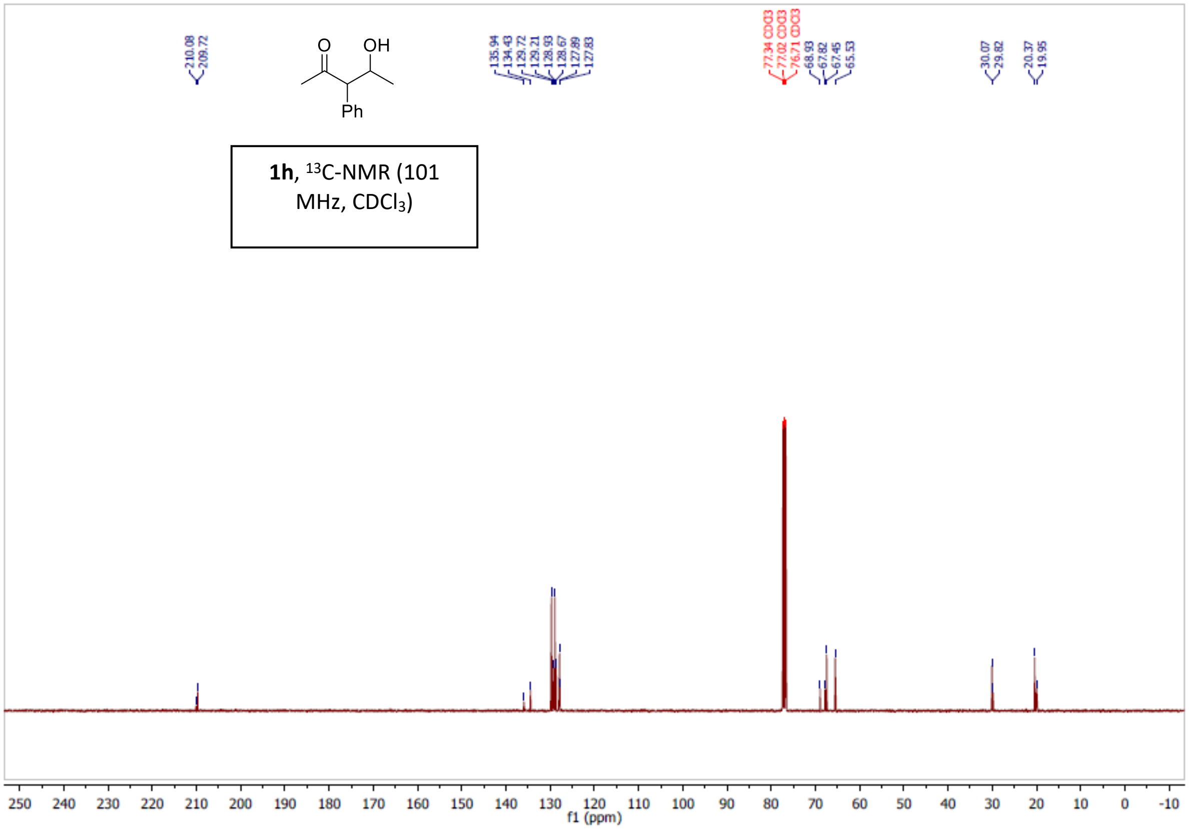
210.08
209.72
O
OH
Ph
135.94
134.43
129.72
129.21
128.93
128.67
127.89
127.83
77.34 CDCl3
77.02 CDCl3
76.71 CDCl3
68.93
67.82
67.45
65.53
30.07
29.82
20.37
19.95
1h, ^{13}C-NMR (101 MHz, $CDCl_3$)
f1 (ppm)

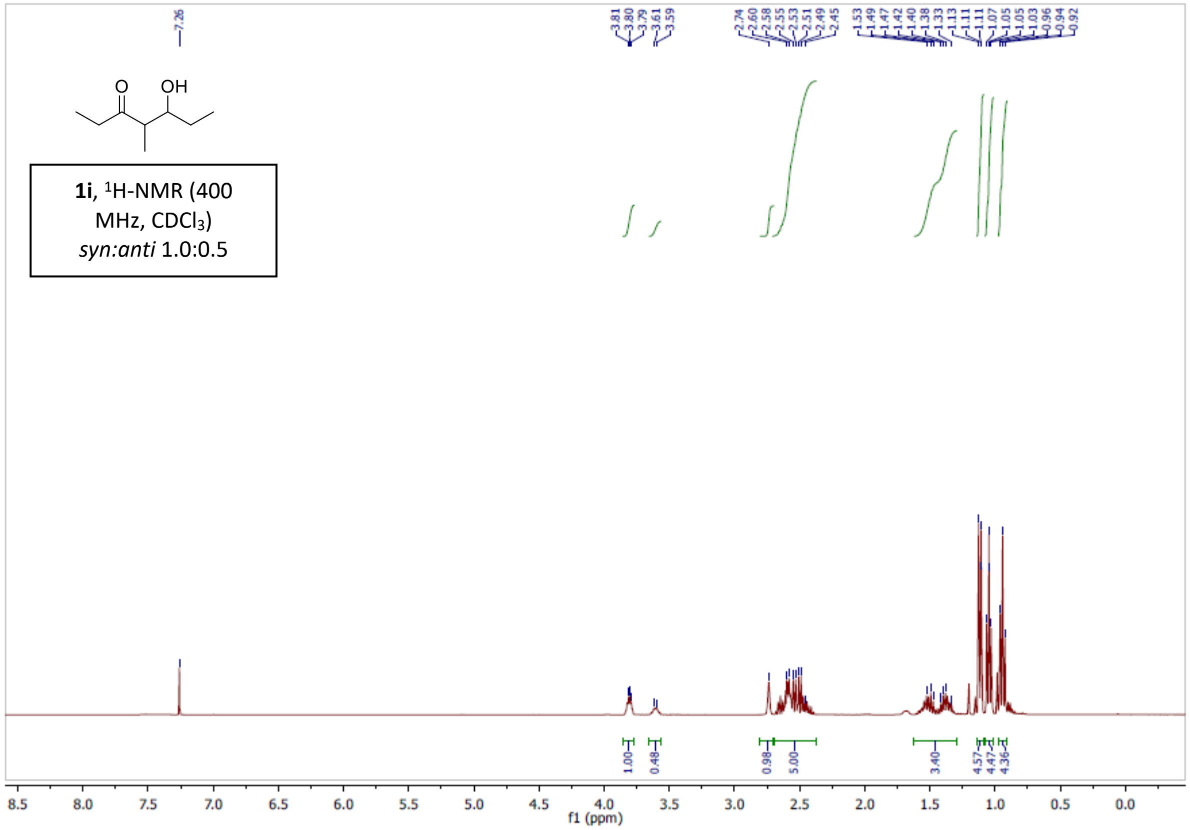
1i, ^{1}H-NMR (400 MHz, CDCl$_3$)
syn:anti 1.0:0.5
f1 (ppm)

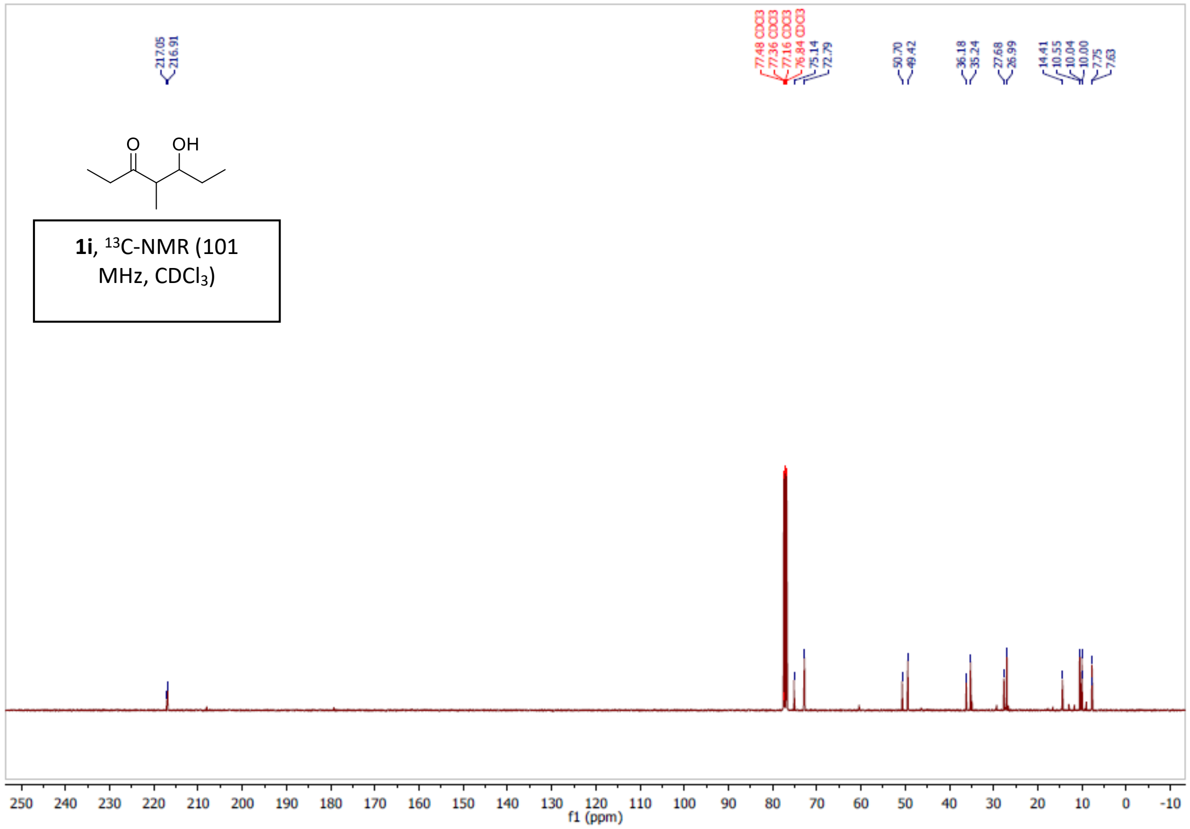
1i, ^{13}C-NMR (101 MHz, CDCl$_3$)
f1 (ppm)

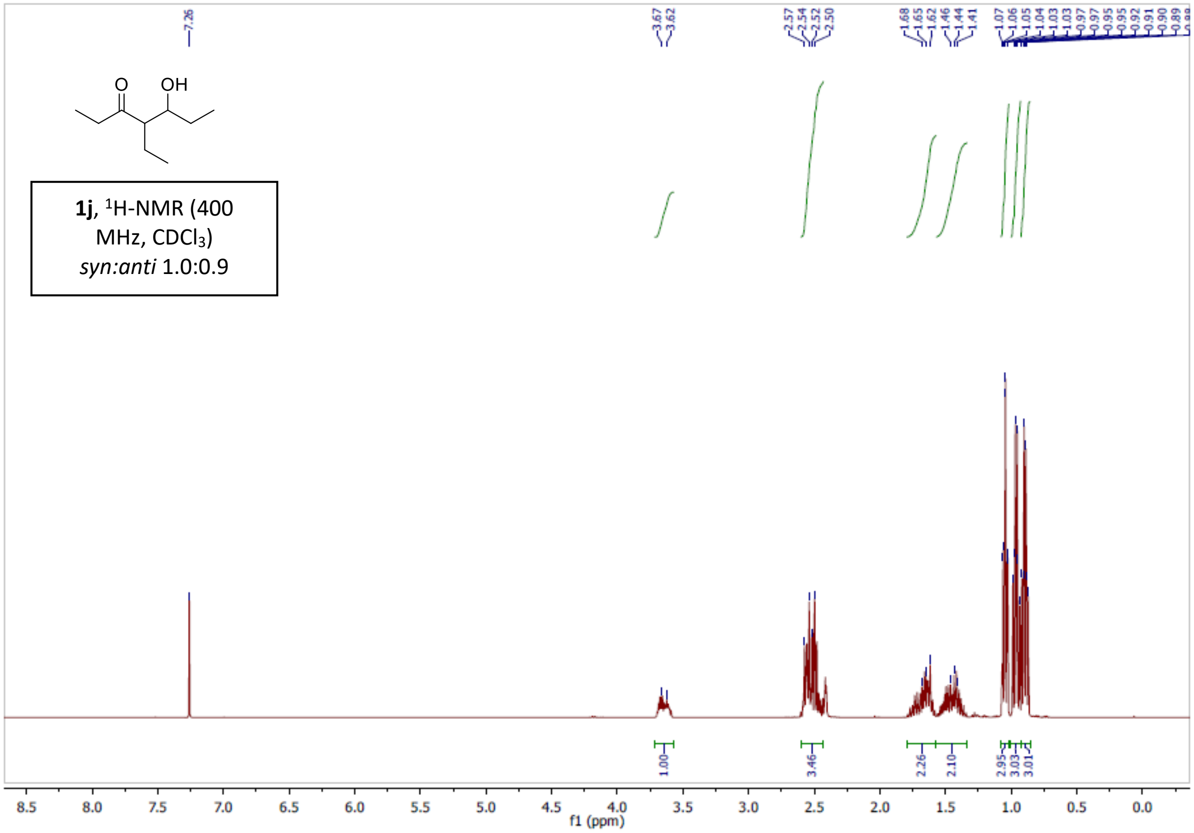
O
OH
1j, 1H-NMR (400 MHz, CDCl3)
syn:anti 1.0:0.9
7.26
3.67
3.62
2.57
2.54
2.52
2.50
1.68
1.65
1.62
1.46
1.44
1.41
1.00
3.46
2.26
2.10
2.95
3.03
3.01
f1 (ppm)

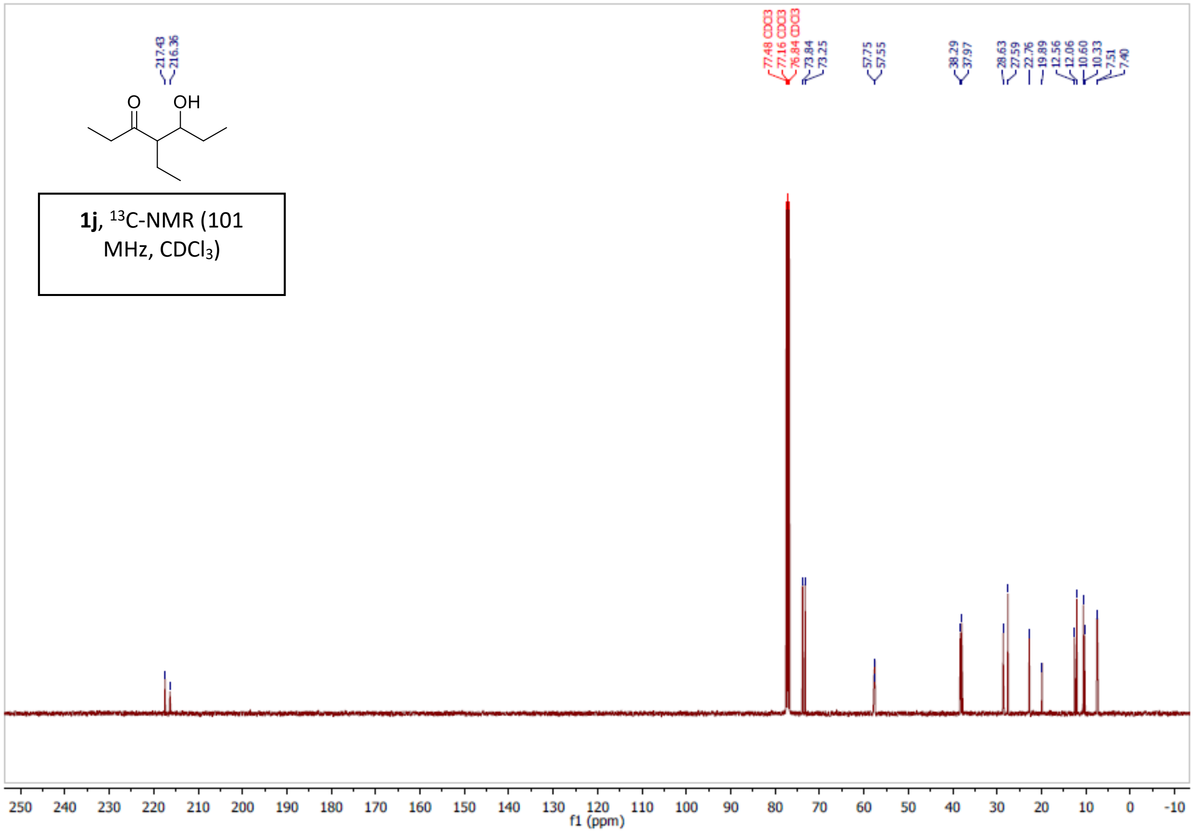
O
OH
1j, 13C-NMR (101 MHz, CDCl3)
217.43
216.36
77.48 CDCl3
77.16 CDCl3
76.84 CDCl3
73.84
73.25
57.75
57.55
38.29
37.97
28.63
27.59
22.76
19.89
12.56
12.06
10.60
10.33
7.51
7.40
f1 (ppm)

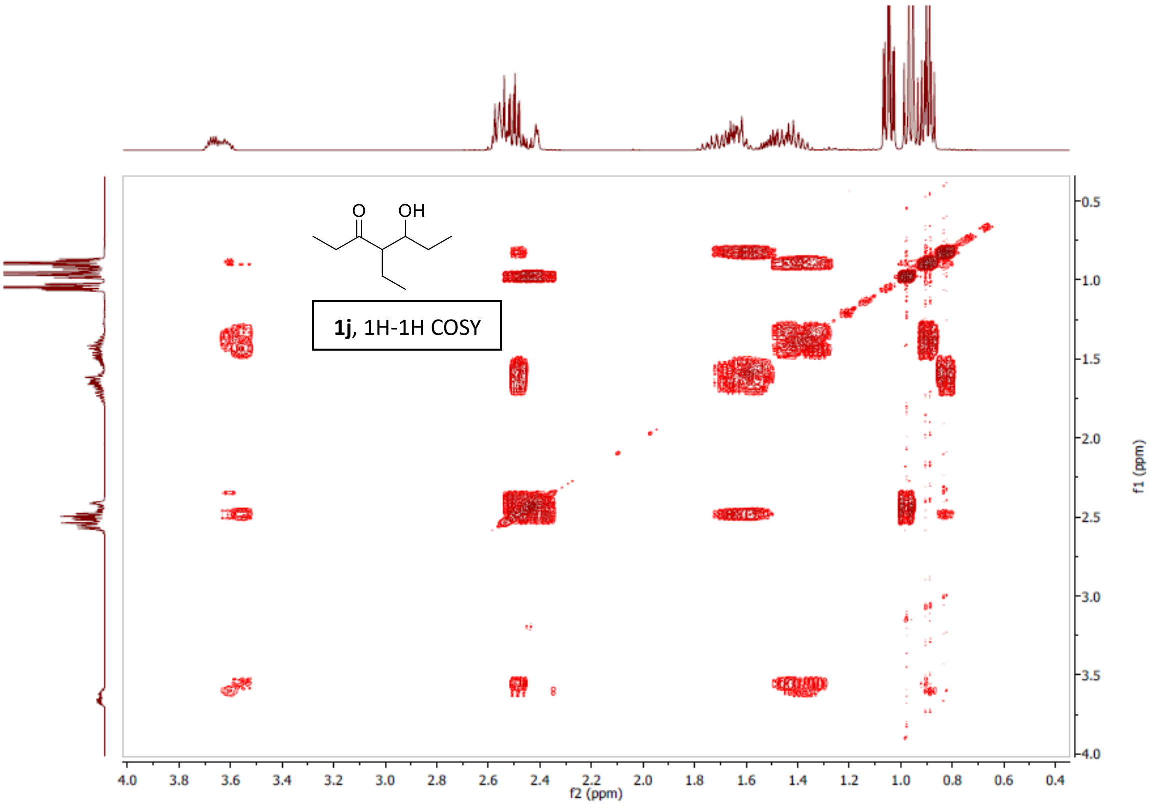
O
OH
1j, 1H-1H COSY
f2 (ppm)
f1 (ppm)
4.0 3.8 3.6 3.4 3.2 3.0 2.8 2.6 2.4 2.2 2.0 1.8 1.6 1.4 1.2 1.0 0.8 0.6 0.4
0.5 1.0 1.5 2.0 2.5 3.0 3.5 4.0

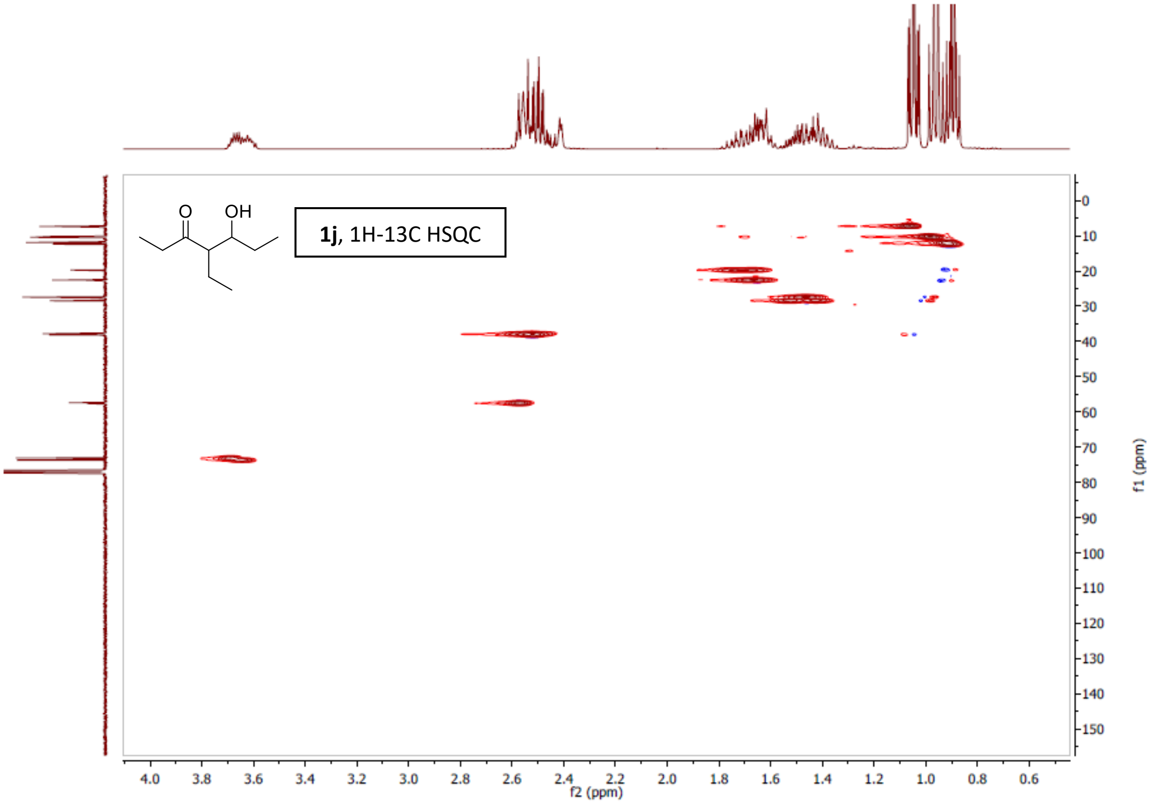
O
OH
1j, 1H-13C HSQC
f2 (ppm)
f1 (ppm)
4.0 3.8 3.6 3.4 3.2 3.0 2.8 2.6 2.4 2.2 2.0 1.8 1.6 1.4 1.2 1.0 0.8 0.6
0 10 20 30 40 50 60 70 80 90 100 110 120 130 140 150

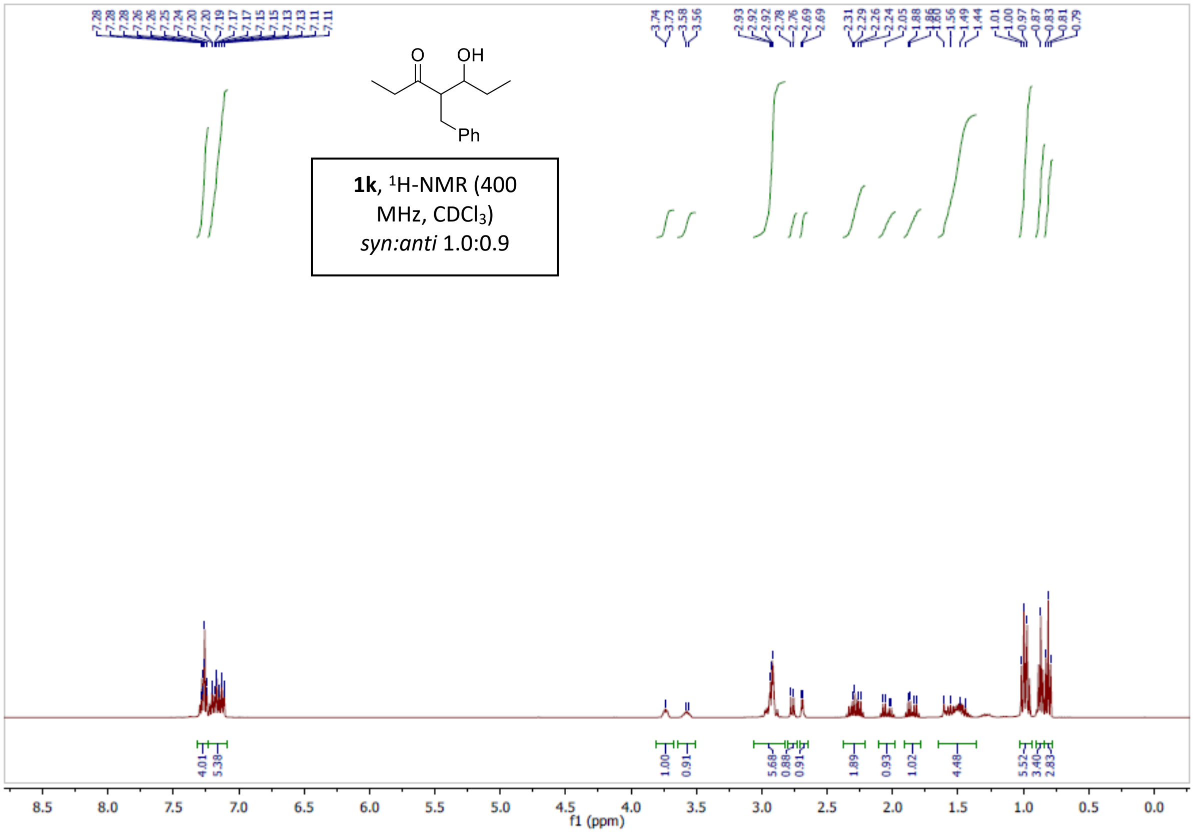
OH
O
Ph
1k, 1H-NMR (400 MHz, CDCl3)
syn:anti 1.0:0.9
f1 (ppm)

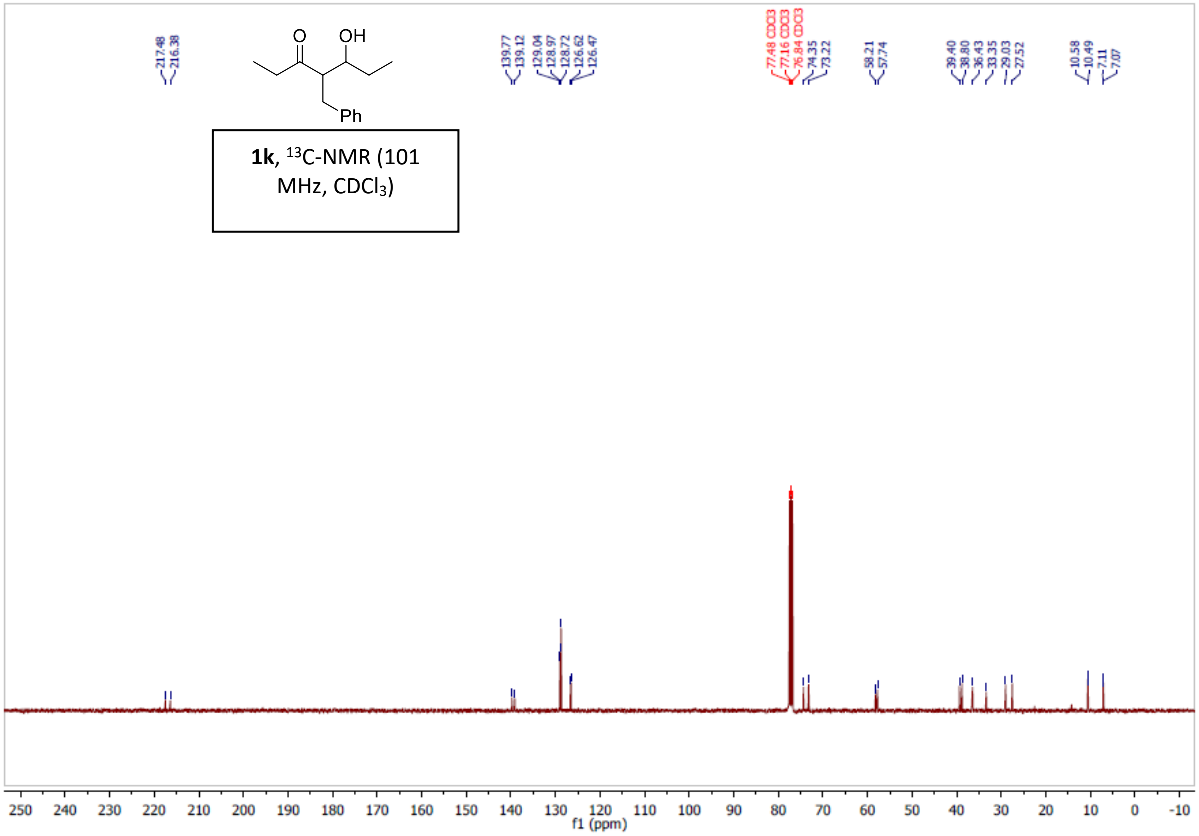
OH
O
Ph
1k, 13C-NMR (101 MHz, CDCl3)
f1 (ppm)

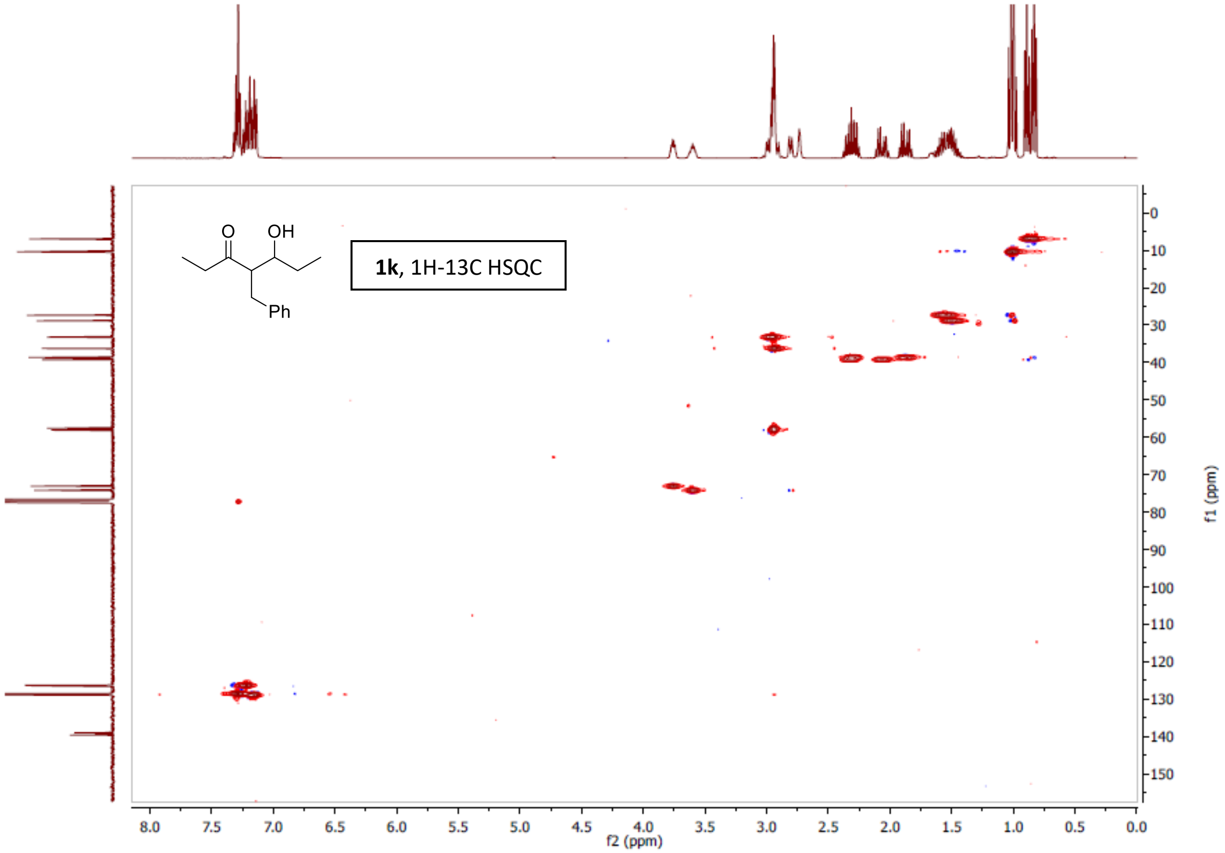
O
OH
Ph
1k, 1H-13C HSQC
0
10
20
30
40
50
60
70
80
90
100
110
120
130
140
150
f1 (ppm)
8.0
7.5
7.0
6.5
6.0
5.5
5.0
4.5
4.0
3.5
3.0
2.5
2.0
1.5
1.0
0.5
0.0
f2 (ppm)

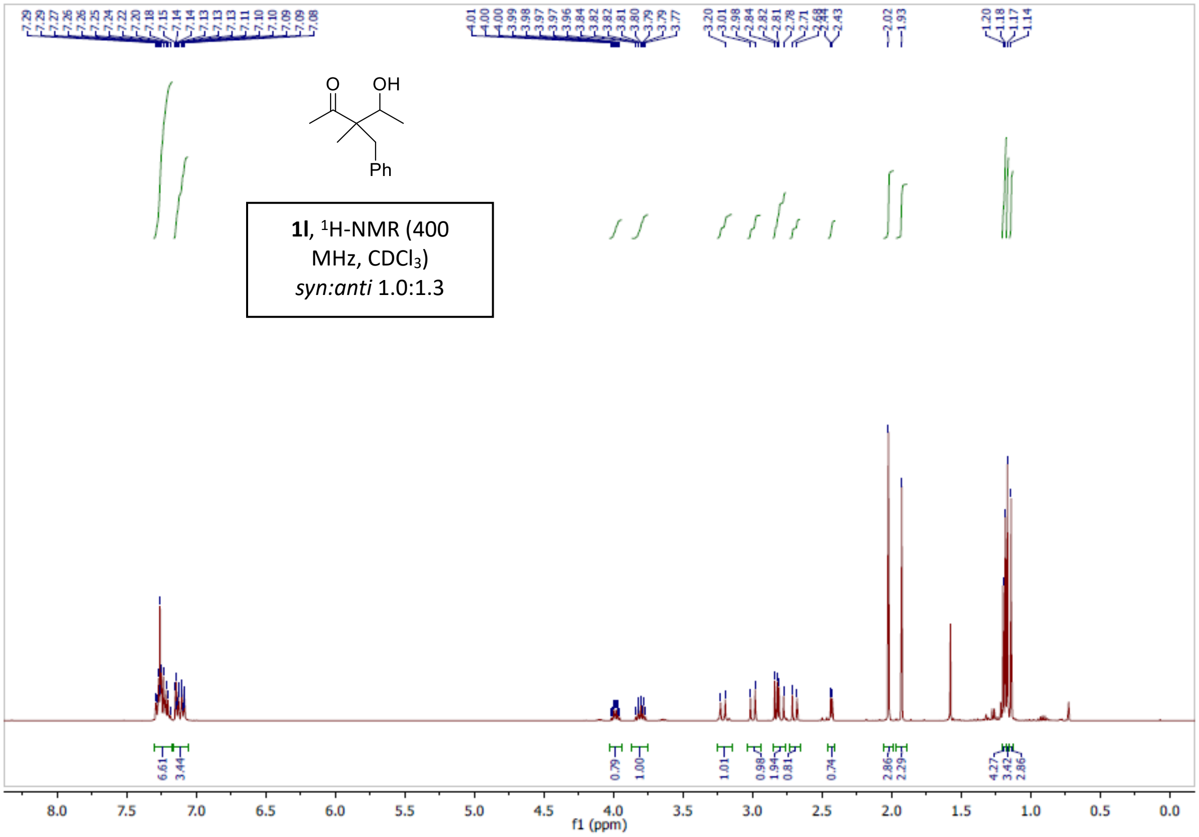
1l, ¹H-NMR (400 MHz, CDCl₃)
syn:anti 1.0:1.3
f1 (ppm)

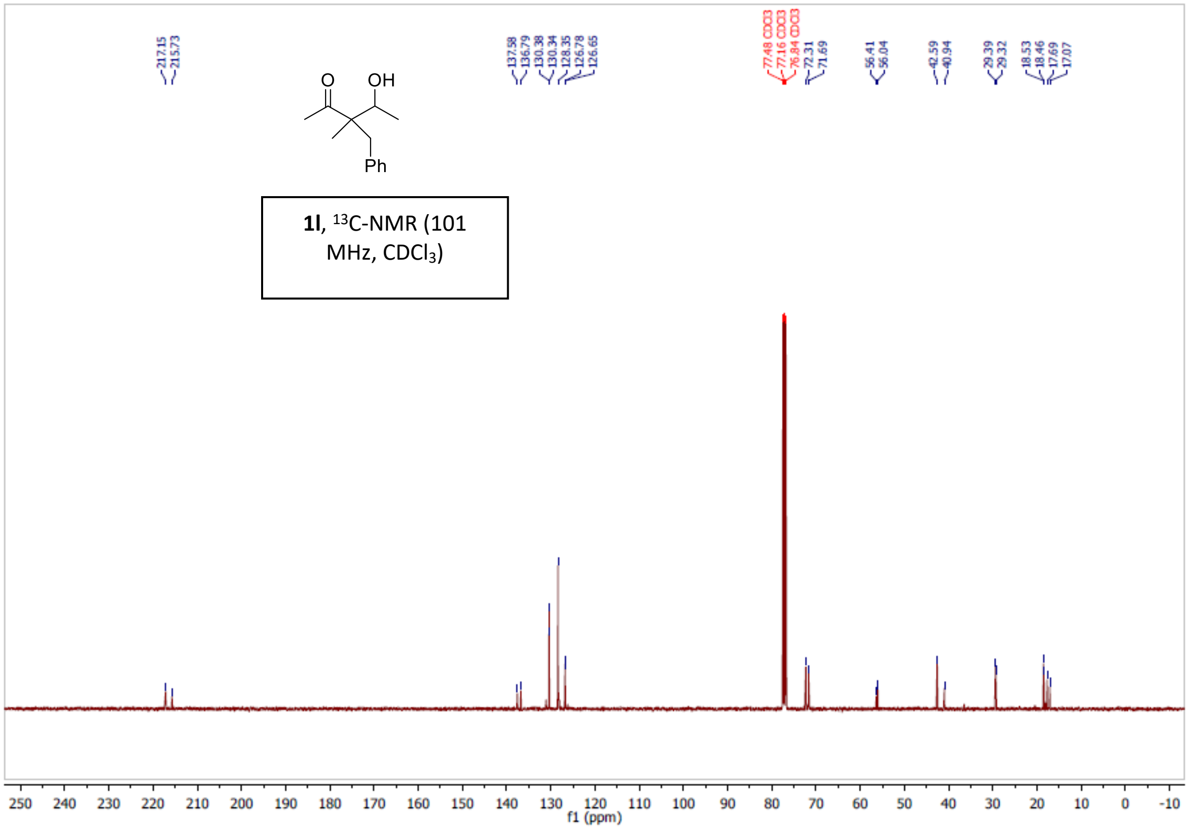
1l, ¹³C-NMR (101 MHz, CDCl₃)
f1 (ppm)

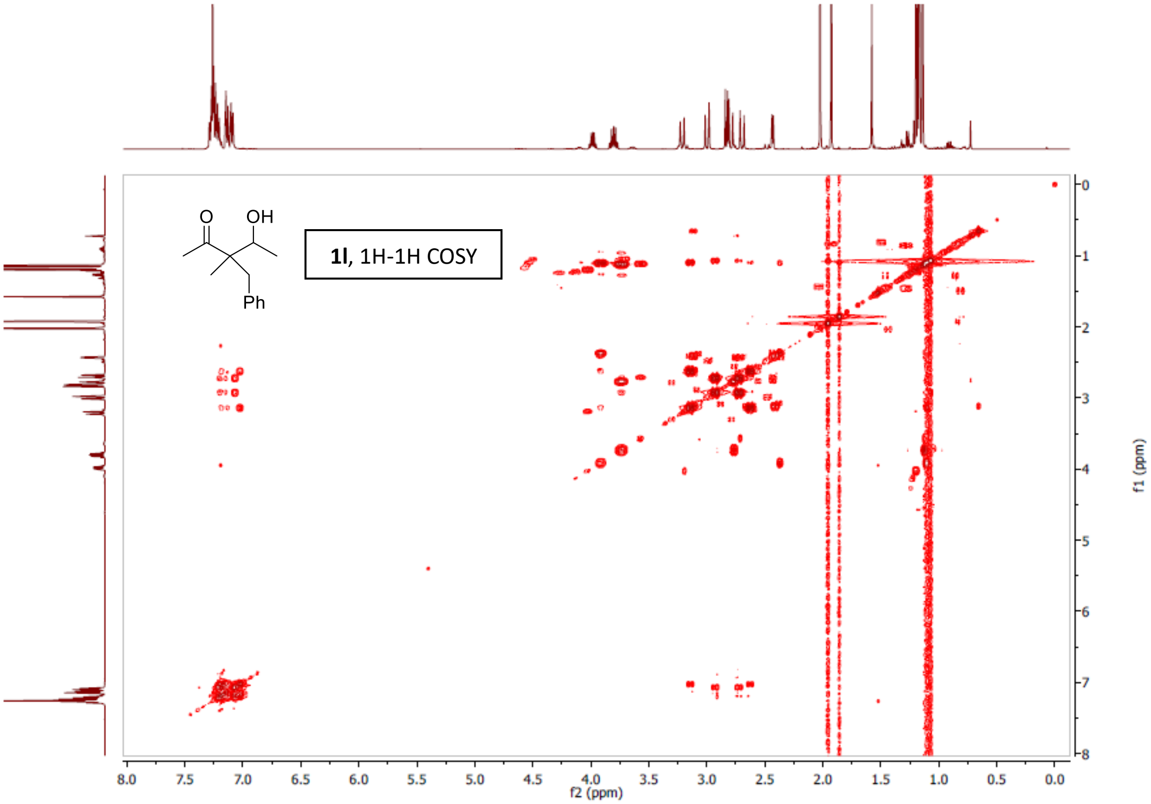
O
OH
Ph
1l, 1H-1H COSY
f1 (ppm)
f2 (ppm)
0
1
2
3
4
5
6
7
8
8.0
7.5
7.0
6.5
6.0
5.5
5.0
4.5
4.0
3.5
3.0
2.5
2.0
1.5
1.0
0.5
0.0

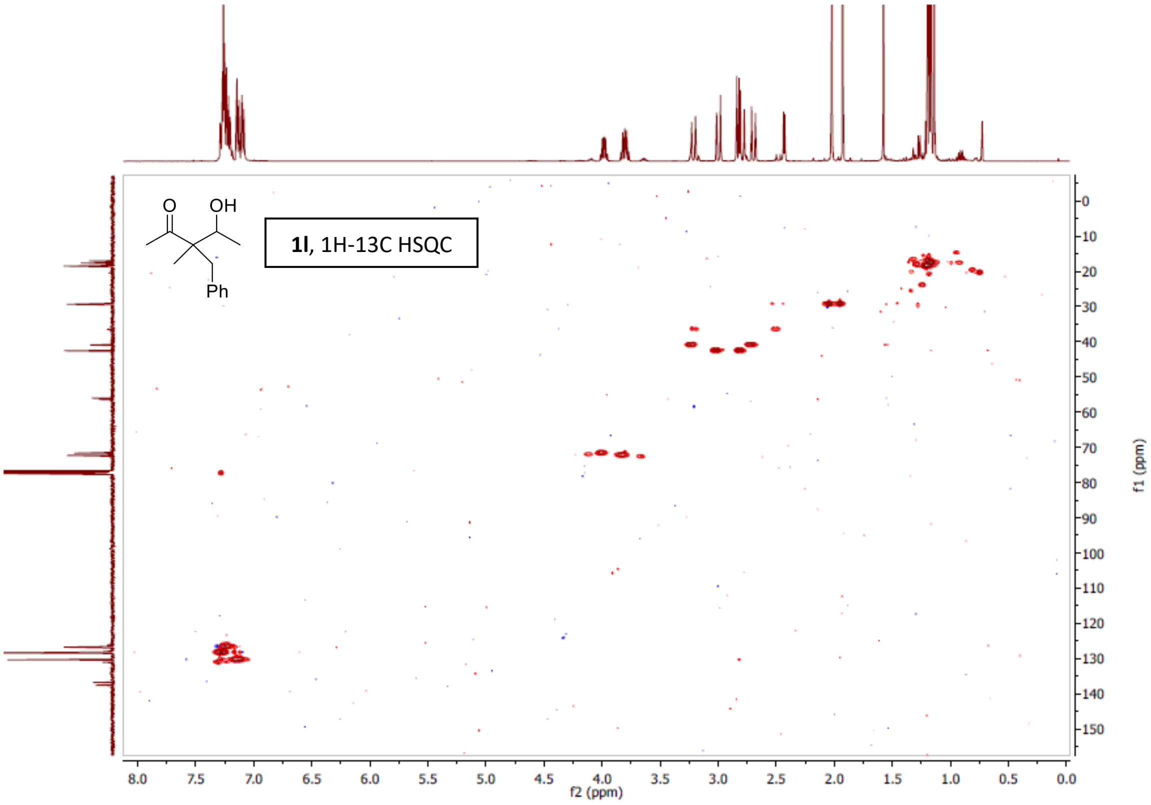
O
OH
Ph
1l, 1H-13C HSQC
f1 (ppm)
f2 (ppm)
0
10
20
30
40
50
60
70
80
90
100
110
120
130
140
150
8.0
7.5
7.0
6.5
6.0
5.5
5.0
4.5
4.0
3.5
3.0
2.5
2.0
1.5
1.0
0.5
0.0

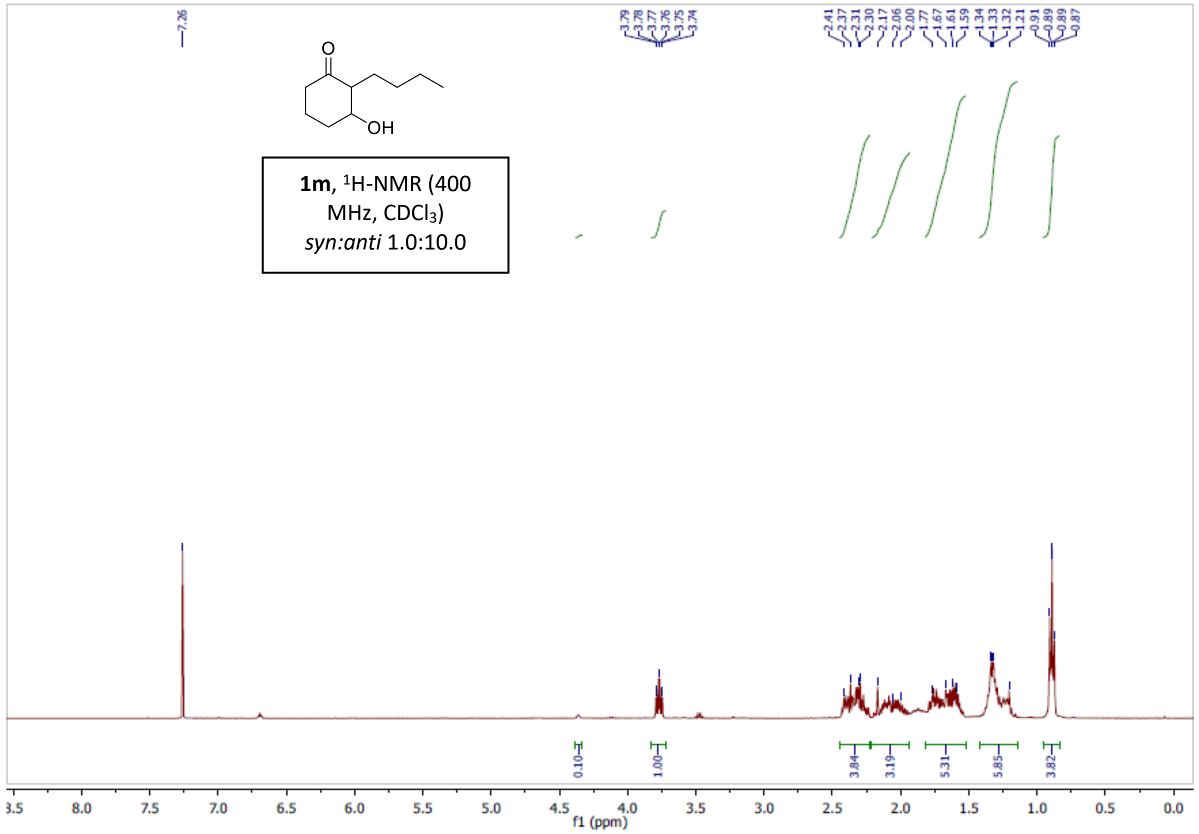

OH
O
1m, ^{1}H-NMR (400 MHz, $CDCl_3$)
syn:anti 1.0:10.0
f1 (ppm)

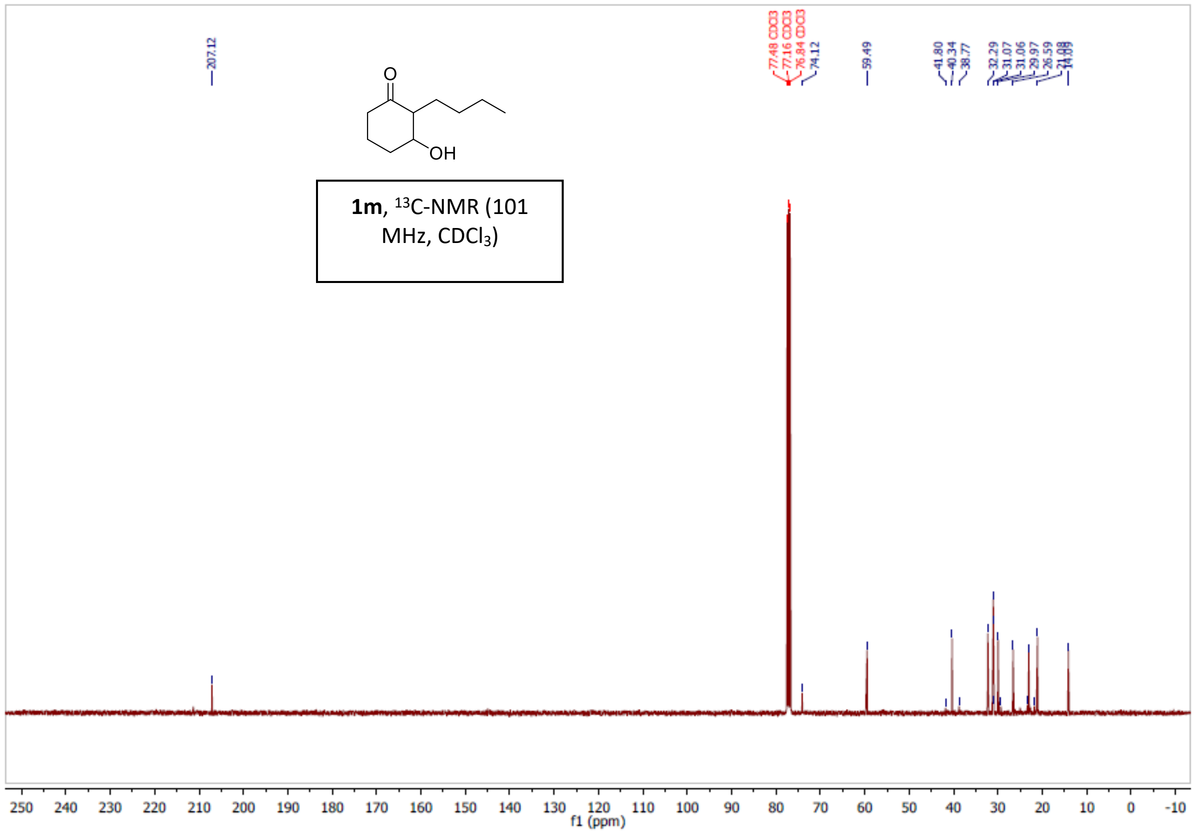

OH
O
1m, ^{13}C-NMR (101 MHz, $CDCl_3$)
f1 (ppm)

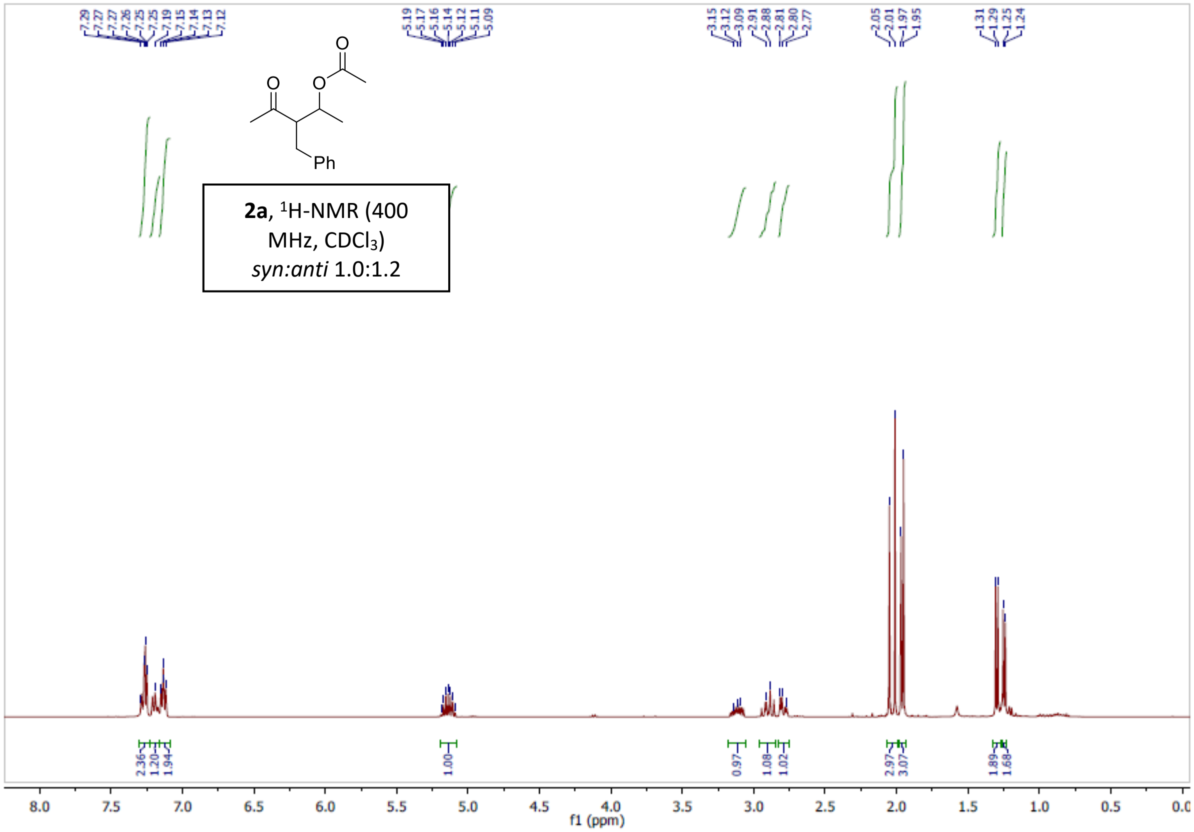

2a, ^{1}H-NMR (400 MHz, $CDCl_3$)
syn:anti 1.0:1.2

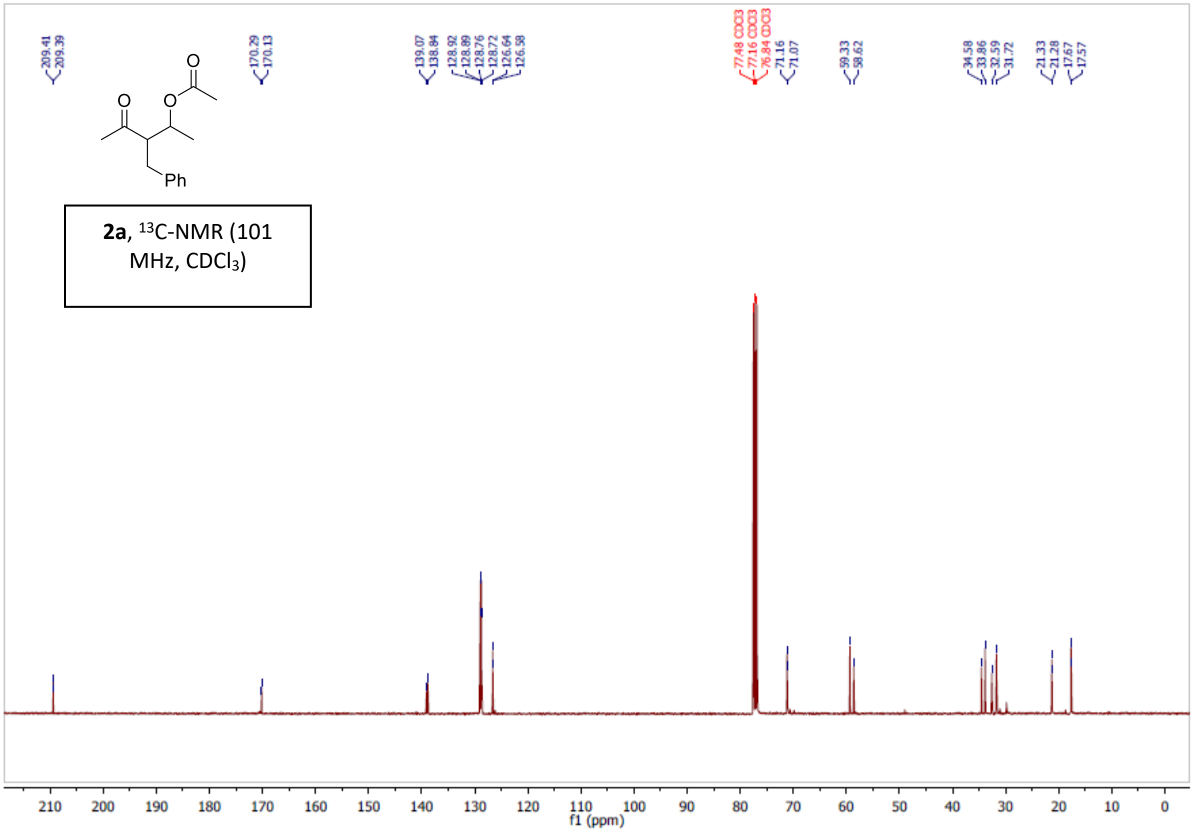

2a, ^{13}C-NMR (101 MHz, $CDCl_3$)

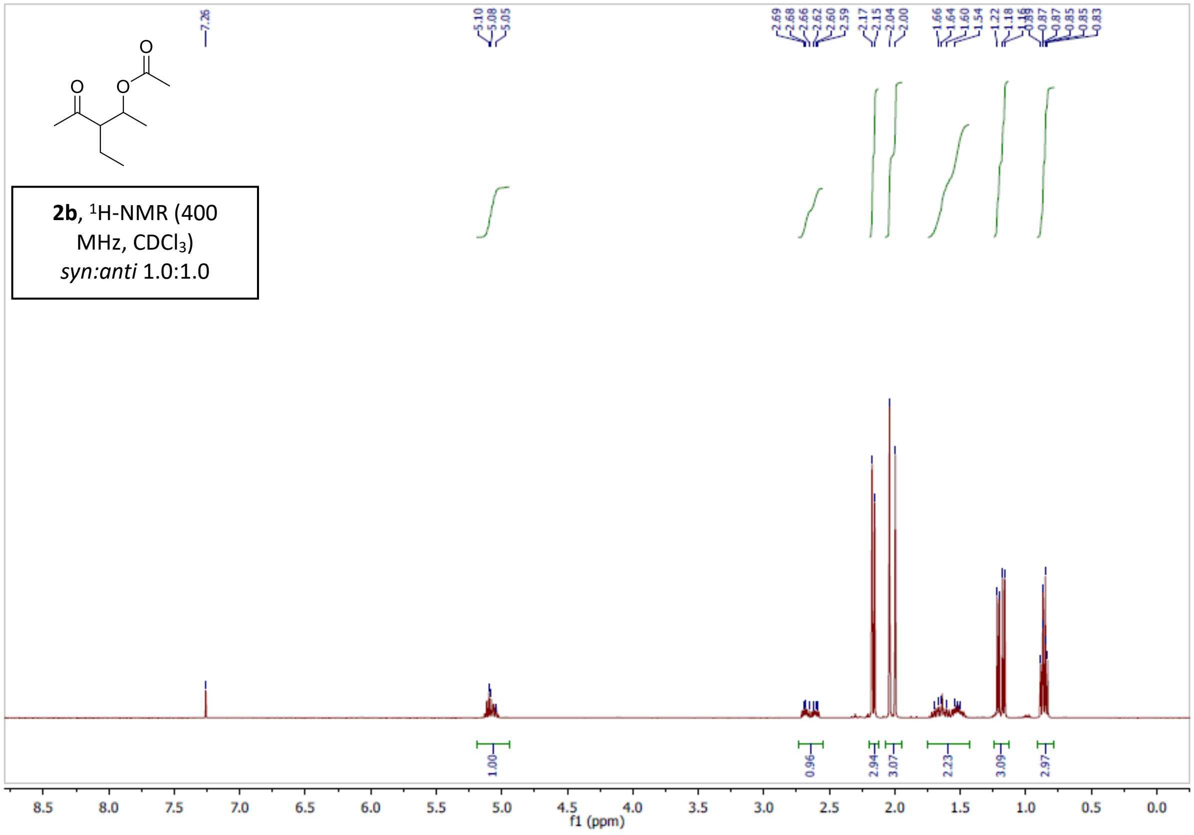
2b, 1H-NMR (400 MHz, CDCl3)
syn:anti 1.0:1.0
f1 (ppm)

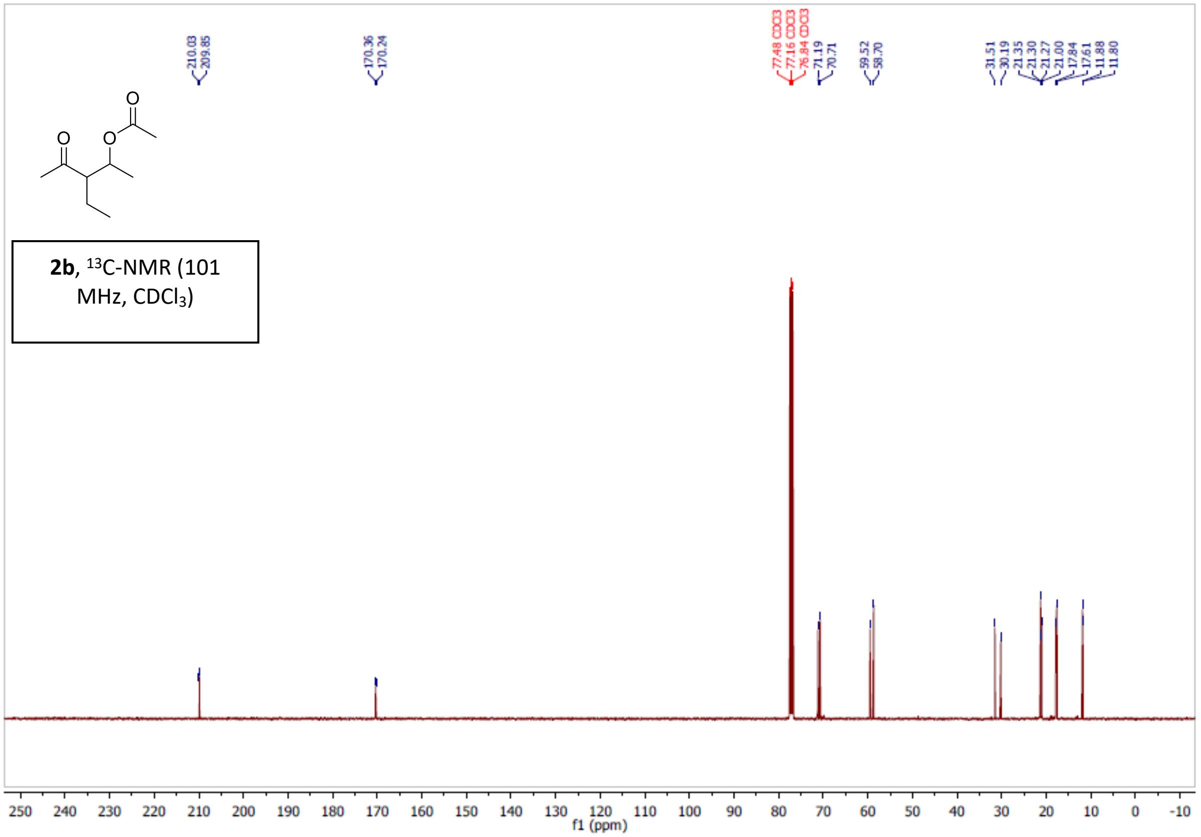
2b, 13C-NMR (101 MHz, CDCl3)
f1 (ppm)

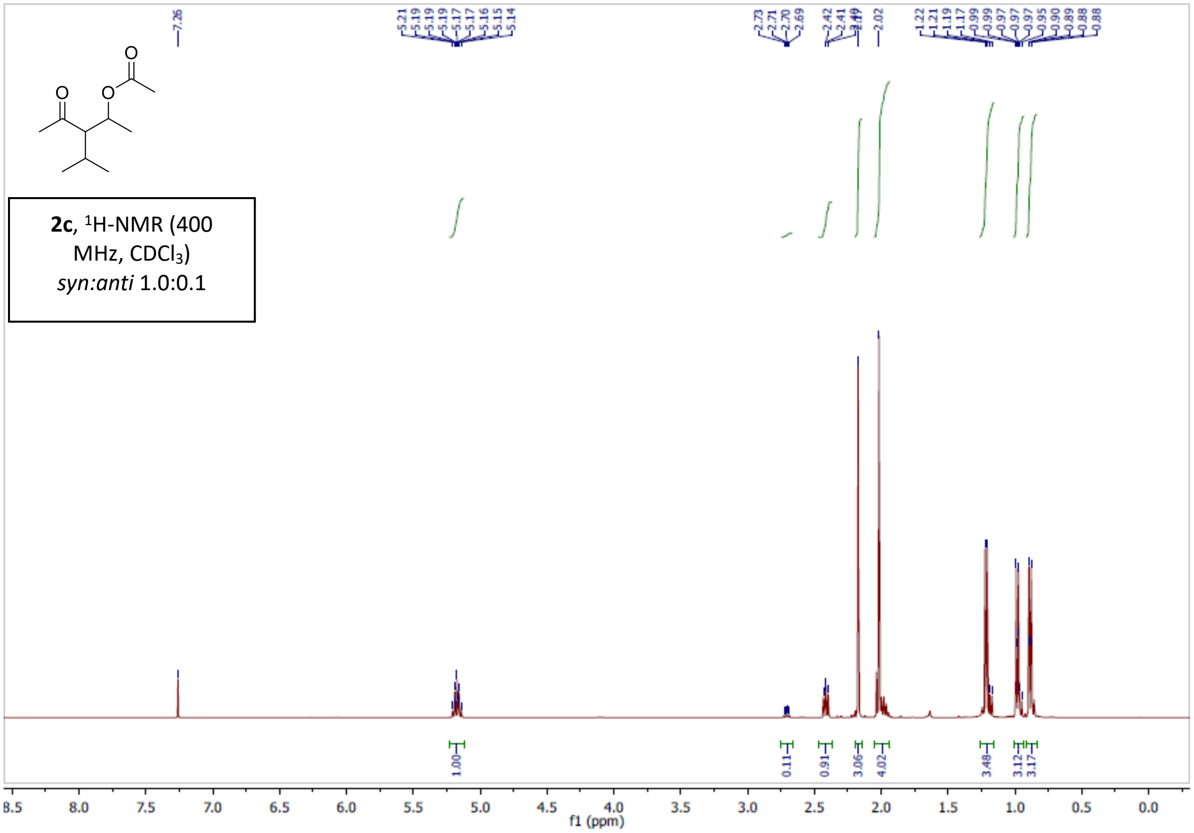
2c, ¹H-NMR (400 MHz, CDCl₃)
syn:anti 1.0:0.1
f1 (ppm)

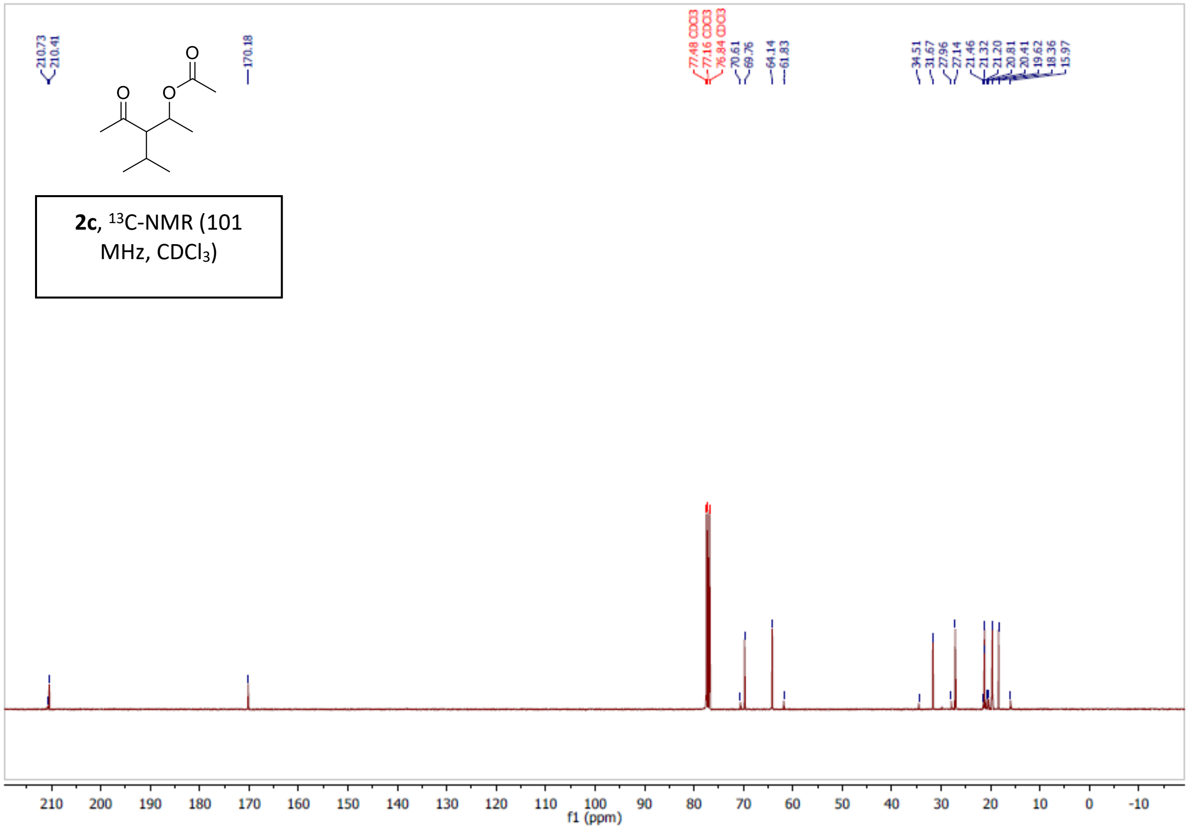
2c, ¹³C-NMR (101 MHz, CDCl₃)
f1 (ppm)

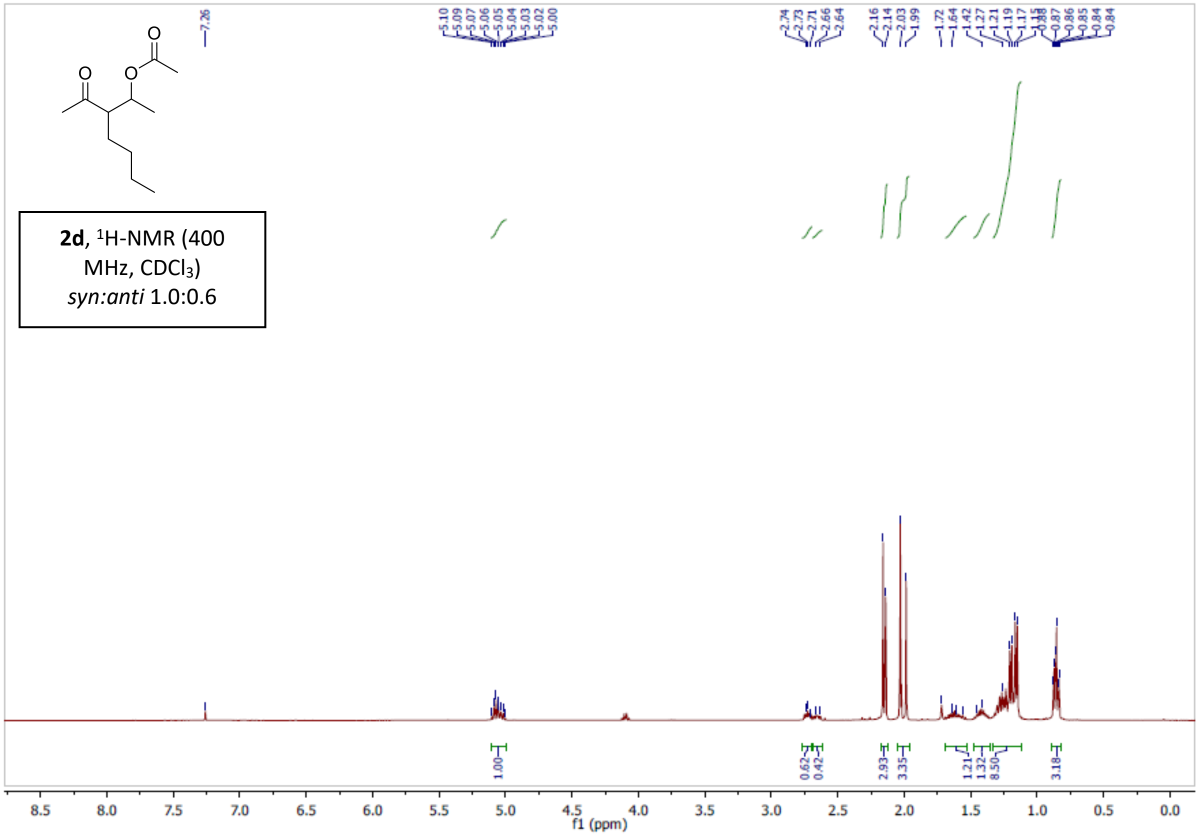
2d, ¹H-NMR (400 MHz, CDCl₃)
syn:anti 1.0:0.6
f1 (ppm)

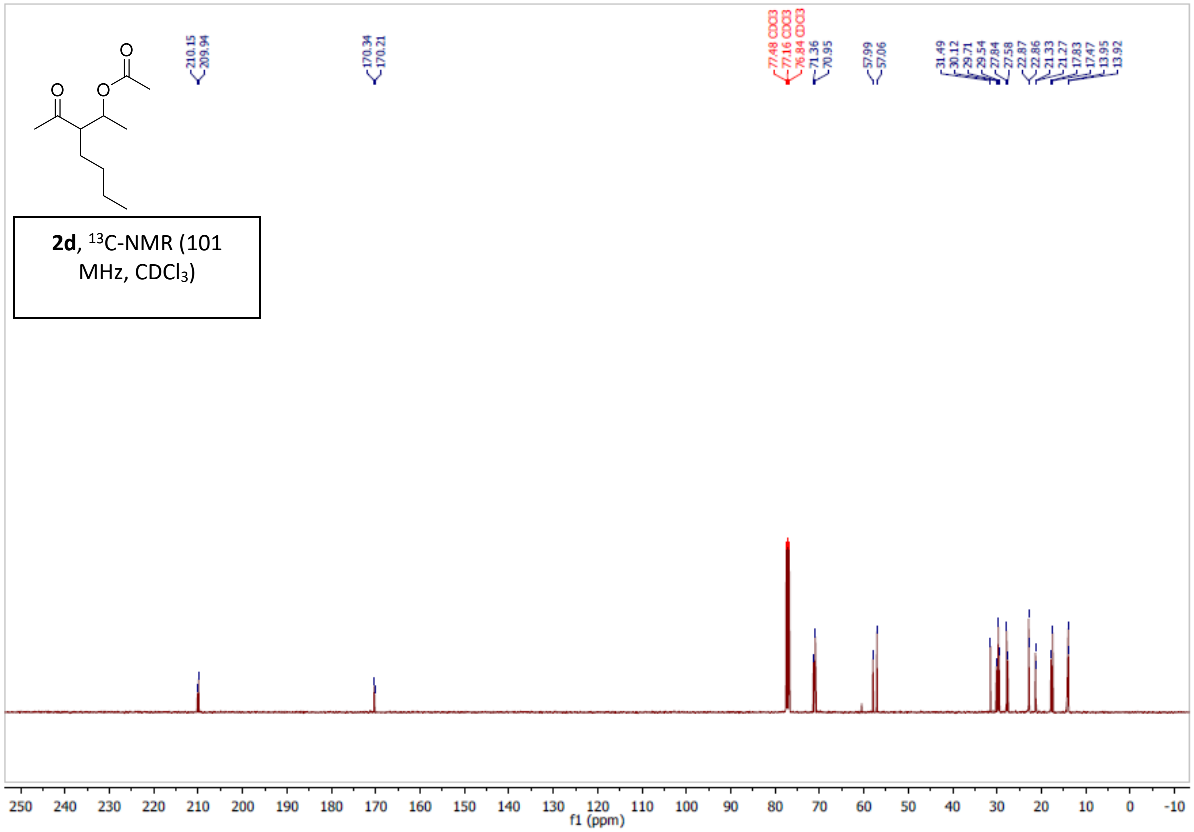
2d, ¹³C-NMR (101 MHz, CDCl₃)
f1 (ppm)

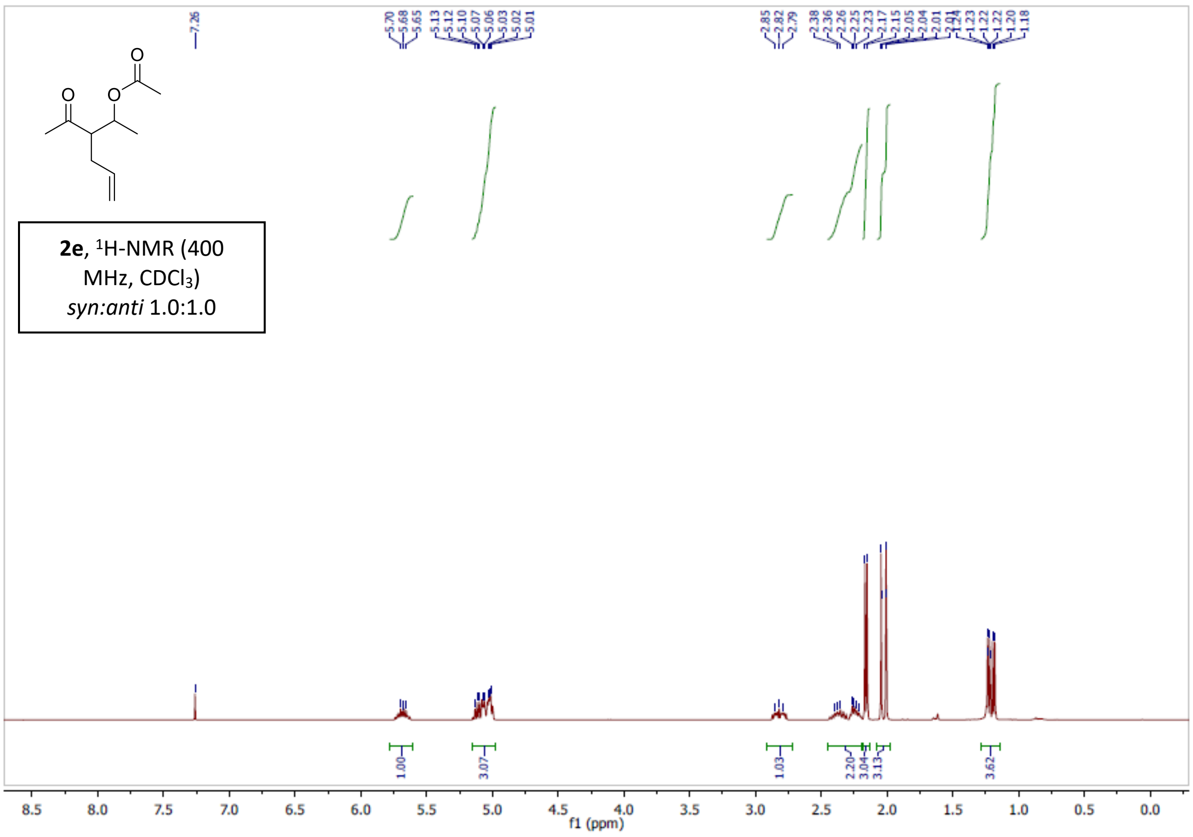
2e, ¹H-NMR (400 MHz, CDCl3) syn:anti 1.0:1.0
f1 (ppm)

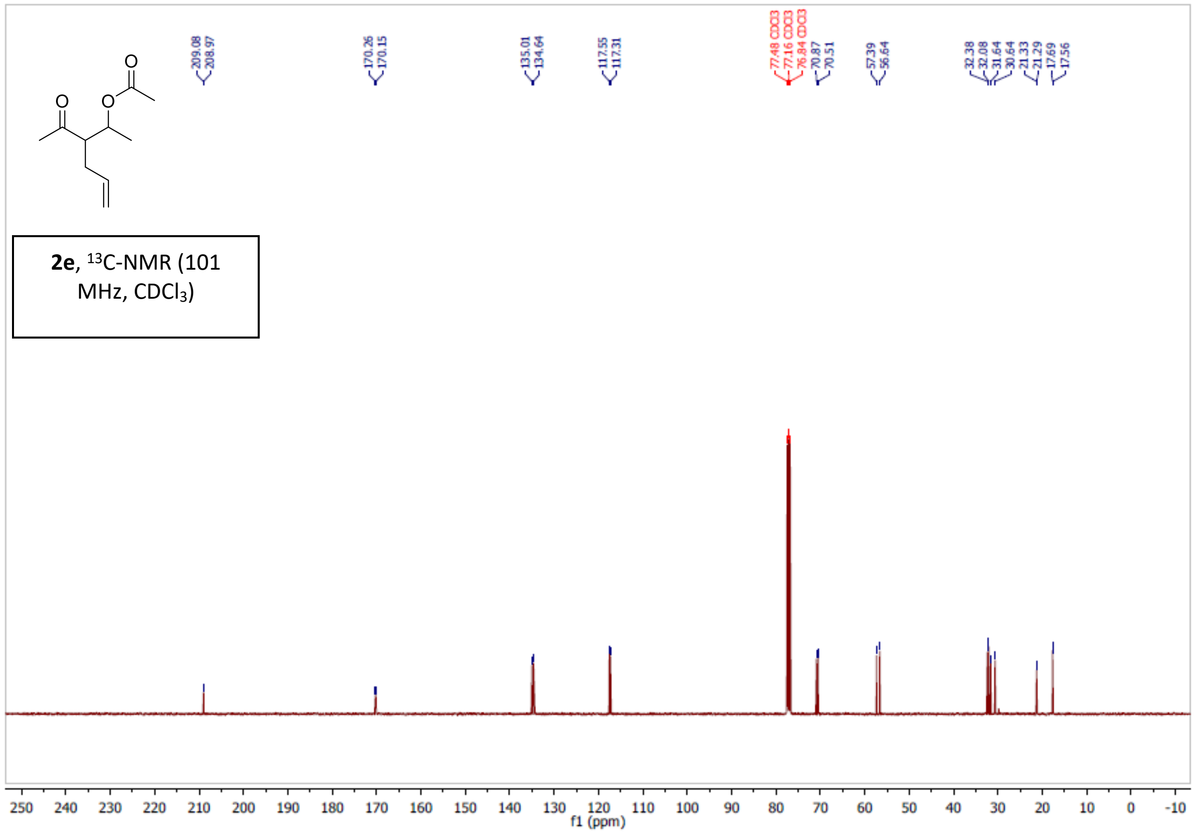
2e, ¹³C-NMR (101 MHz, CDCl3)
f1 (ppm)

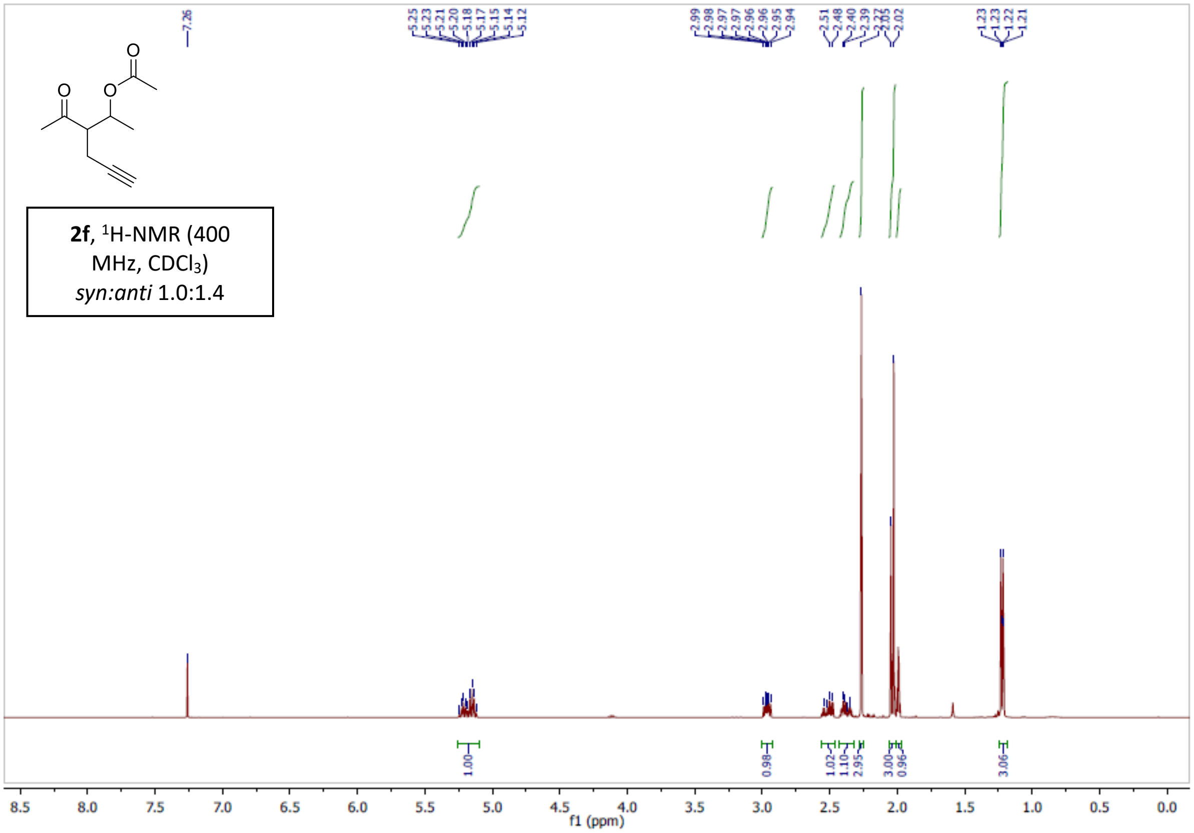
2f, ^{1}H-NMR (400 MHz, $CDCl_3$)
syn:anti 1.0:1.4
f1 (ppm)

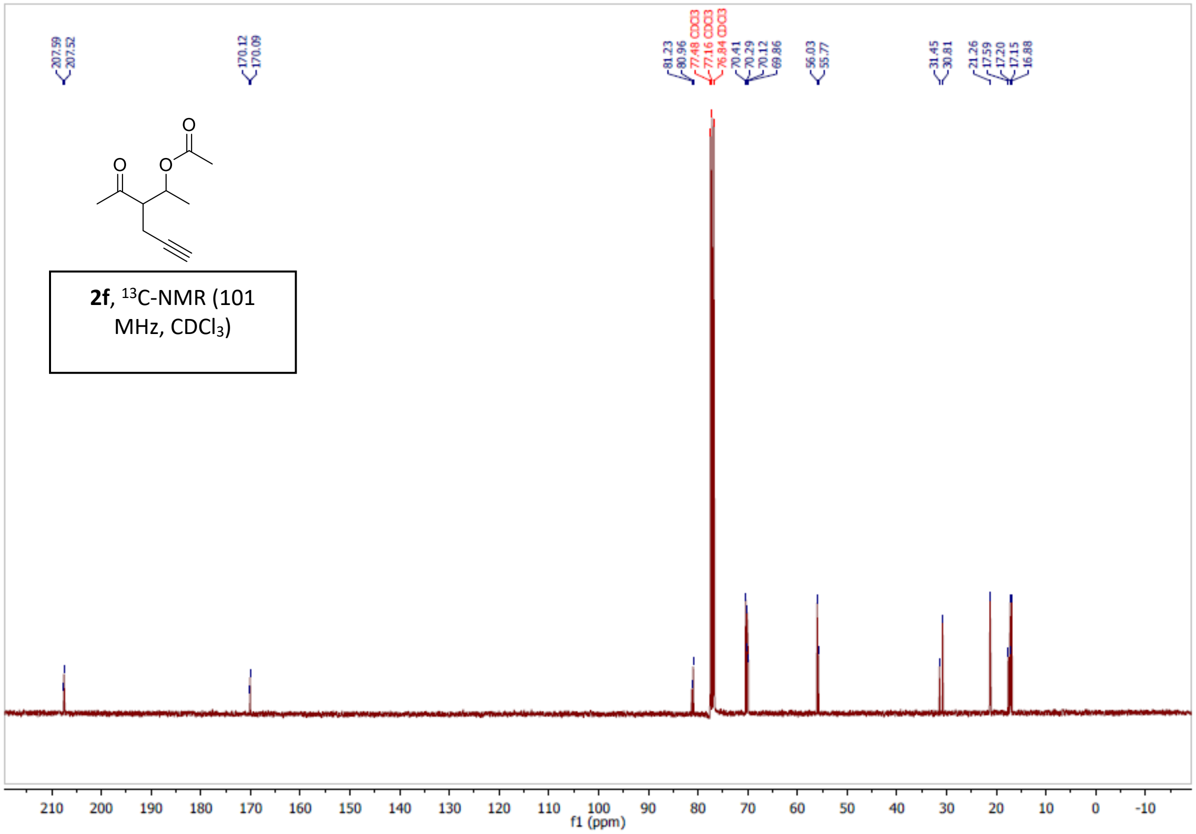
2f, ^{13}C-NMR (101 MHz, $CDCl_3$)
f1 (ppm)

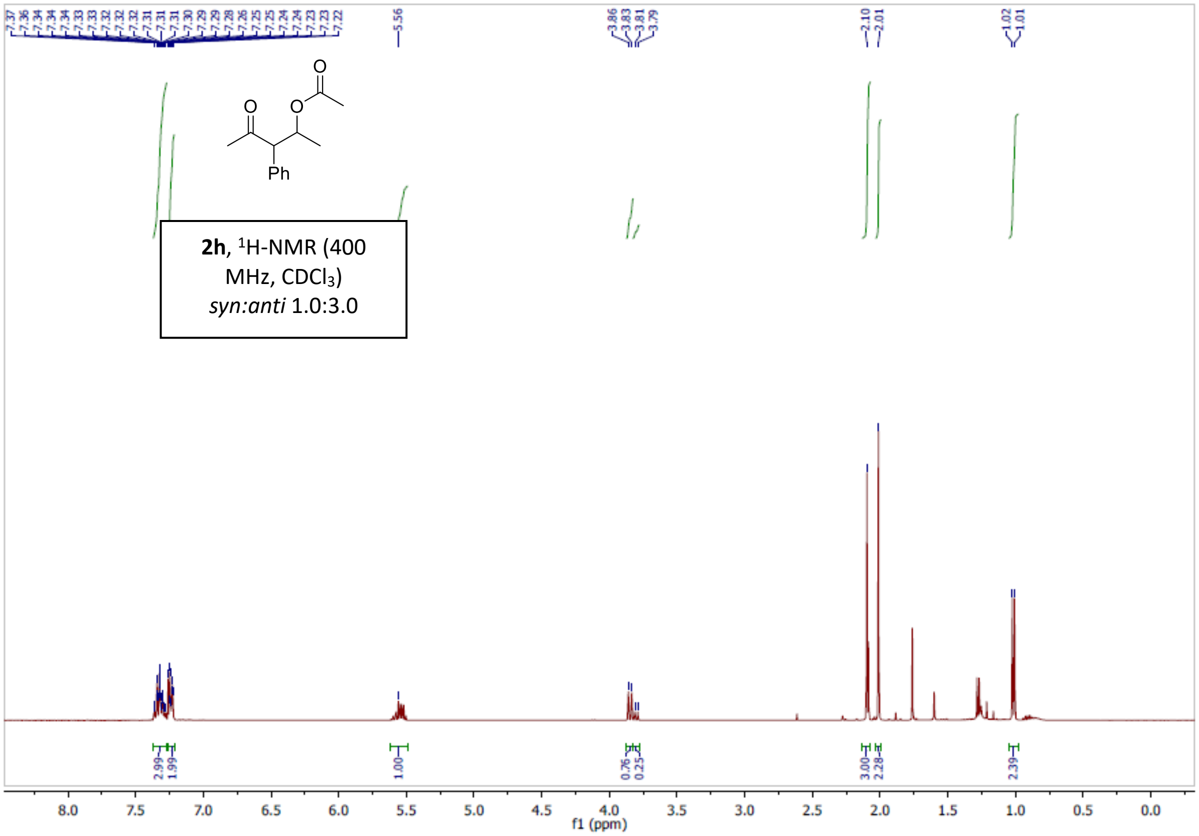
2h, 1H-NMR (400 MHz, CDCl3)
syn:anti 1.0:3.0
Ph
f1 (ppm)

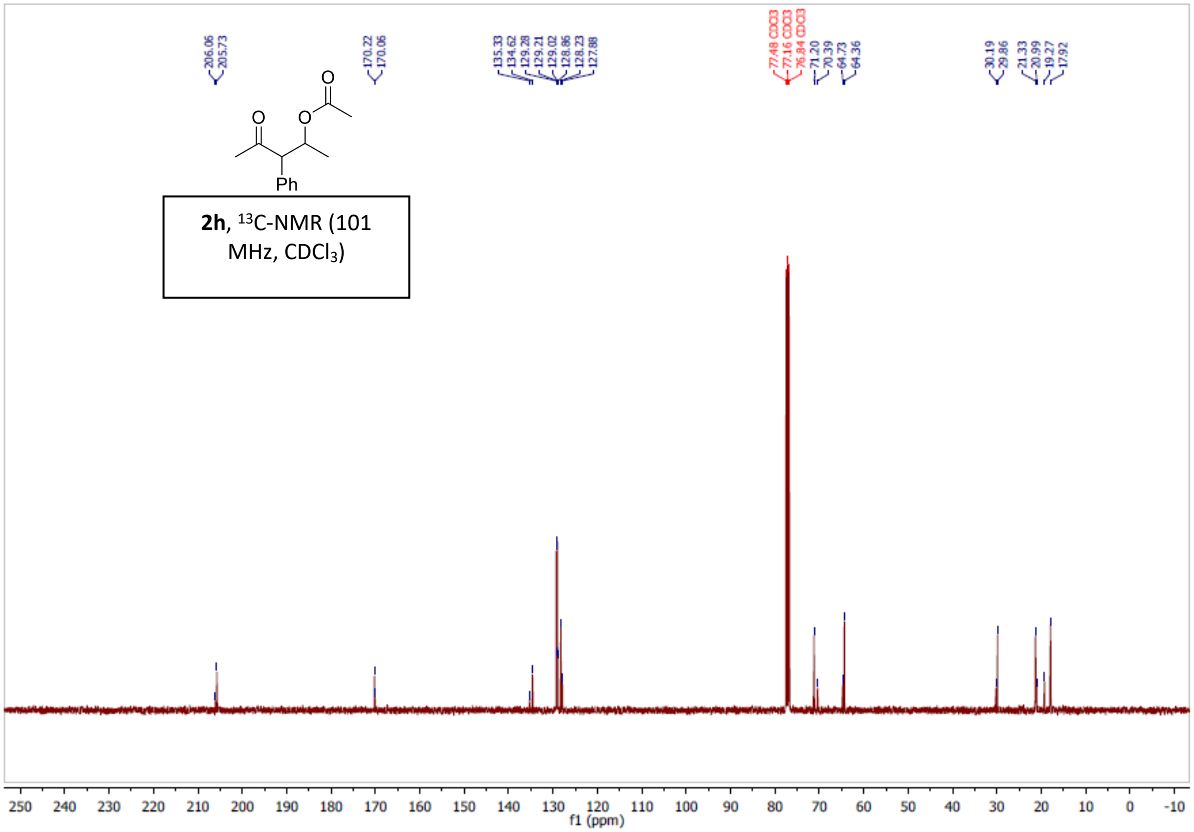
2h, 13C-NMR (101 MHz, CDCl3)
Ph
f1 (ppm)

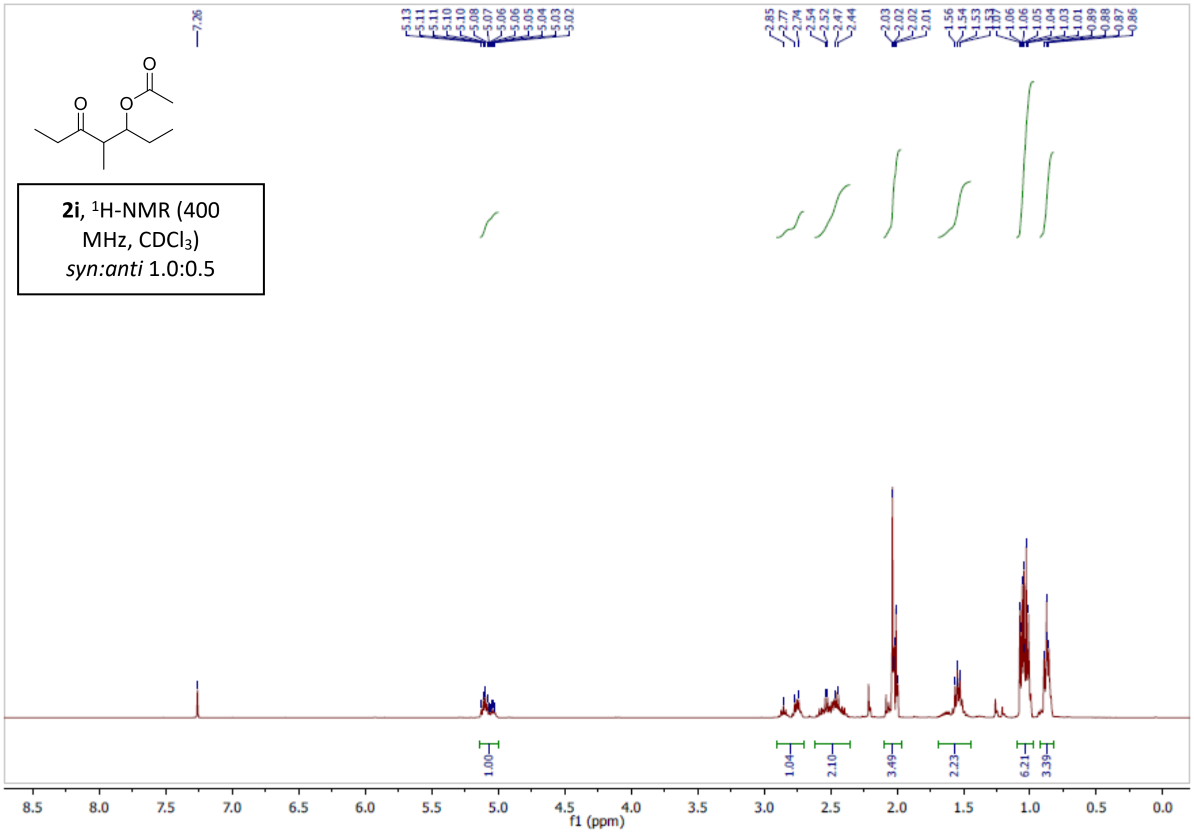
2i, ¹H-NMR (400 MHz, CDCl₃)
syn:anti 1.0:0.5
f1 (ppm)

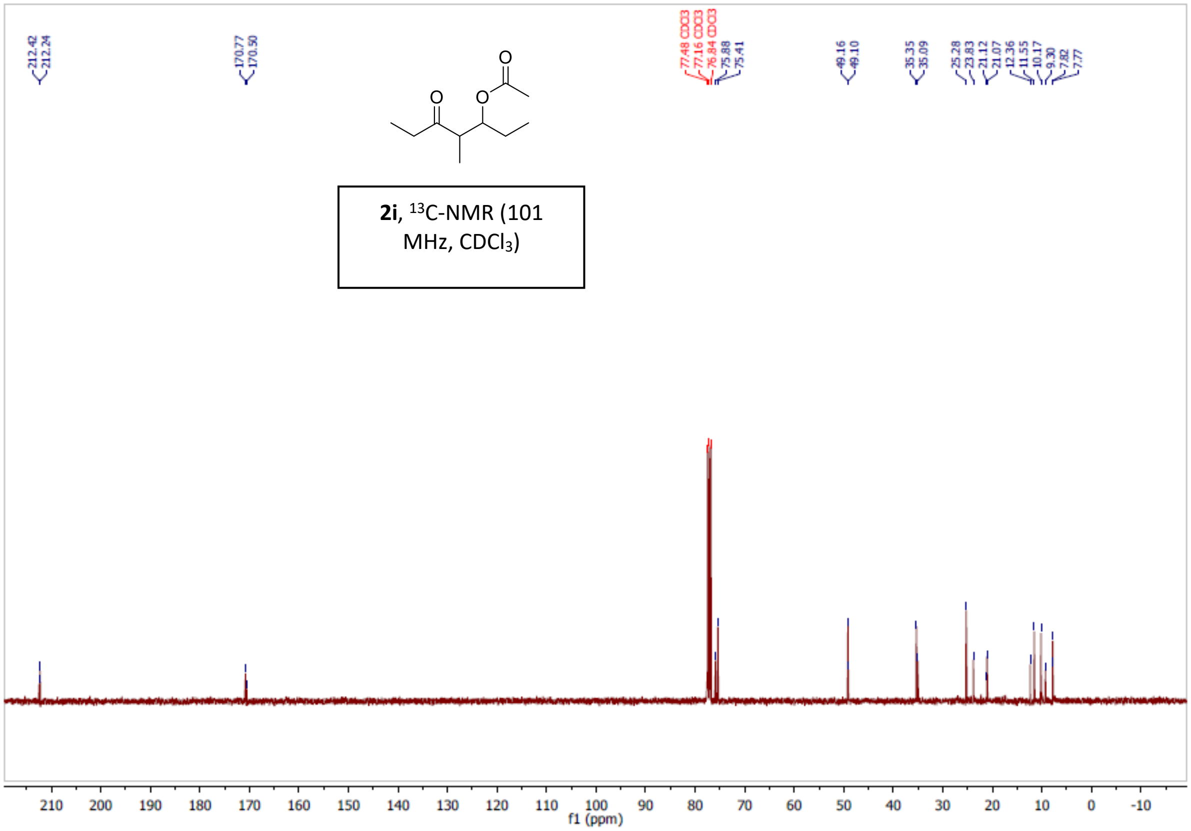
2i, ¹³C-NMR (101 MHz, CDCl₃)
f1 (ppm)

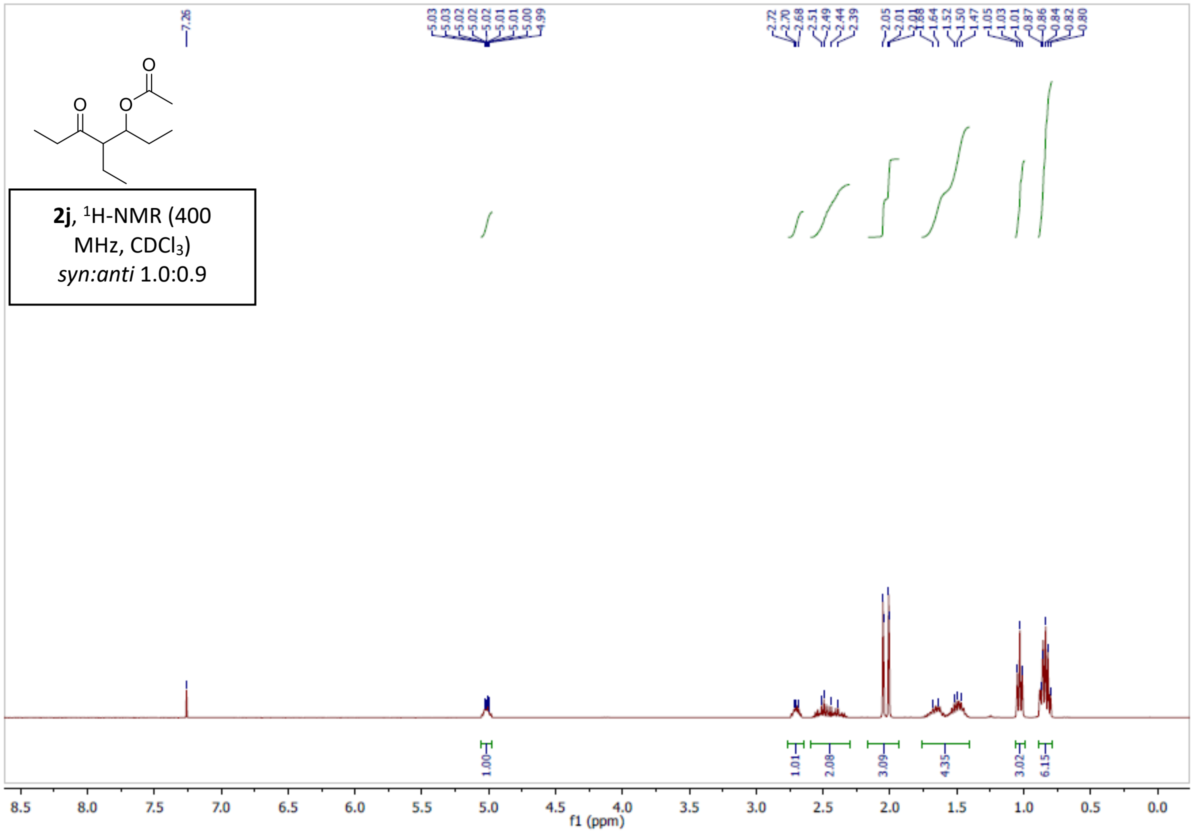

2j, ^{1}H-NMR (400 MHz, $CDCl_3$)
syn:anti 1.0:0.9

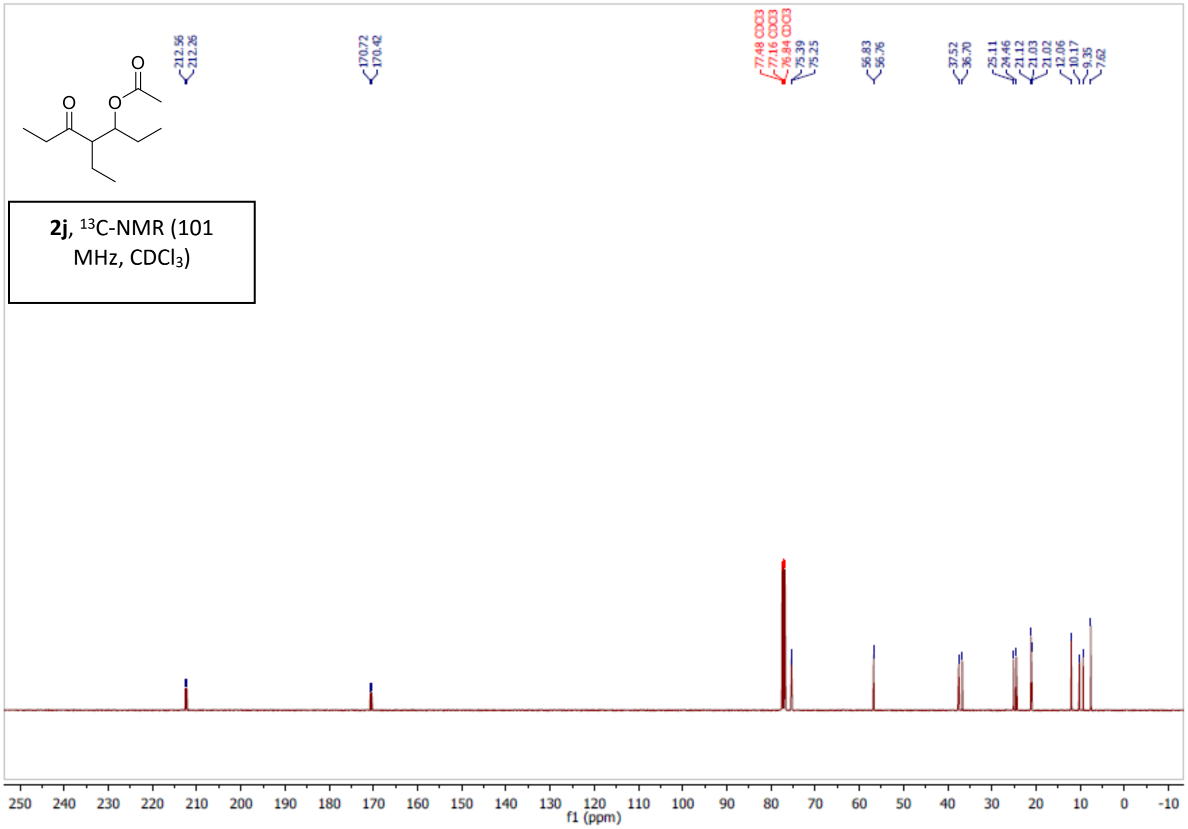

2j, ^{13}C-NMR (101 MHz, $CDCl_3$)

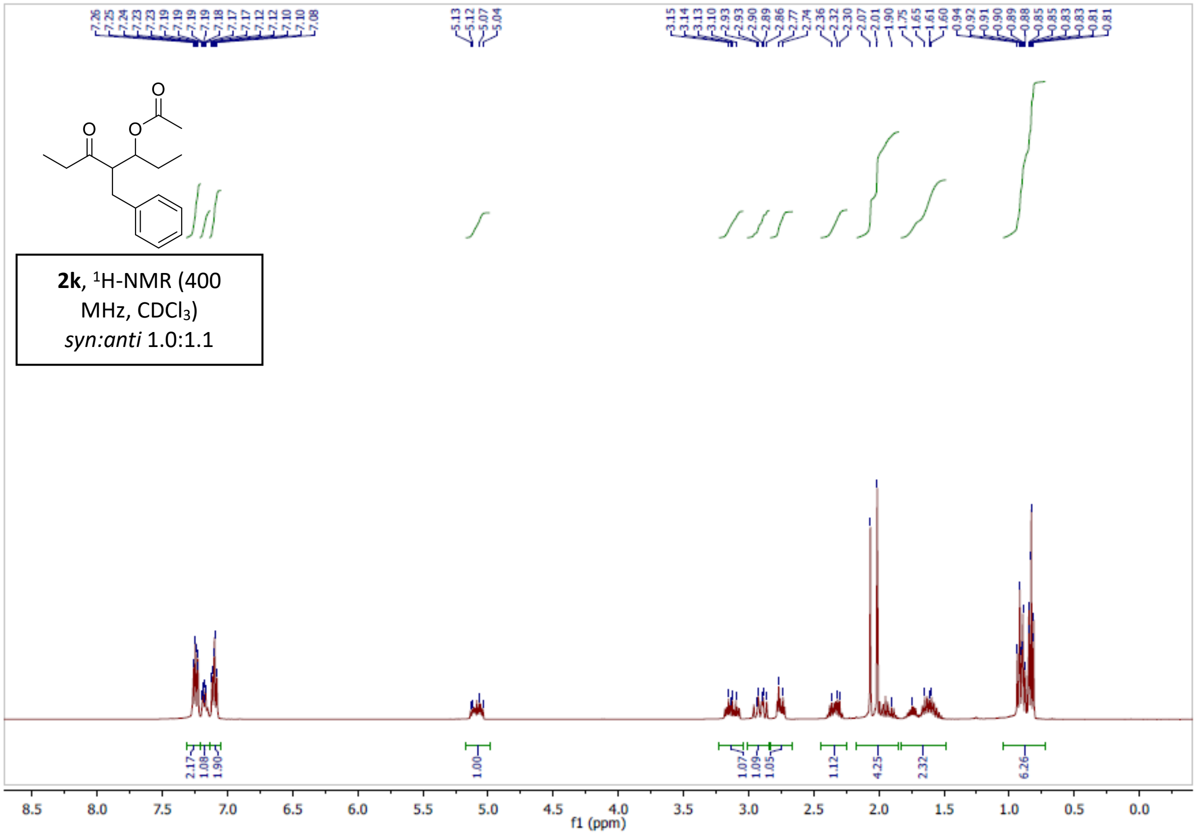
7.26 7.25 7.24 7.23 7.23 7.19 7.19 7.19 7.18 7.17 7.17 7.12 7.12 7.10 7.10 7.08
5.13 5.12 5.07 5.04
3.15 3.14 3.13 3.10 2.93 2.93 2.90 2.89 2.86 2.77 2.74 2.36 2.32 2.30 2.07 2.01 1.90 1.75 1.65 1.61 1.60 0.94 0.92 0.91 0.90 0.90 0.89 0.88 0.88 0.85 0.85 0.83 0.83 0.81 0.81
O
O
O
2k, ^{1}H-NMR (400 MHz, $CDCl_3$)
syn:anti 1.0:1.1
2.17 1.08 1.90 1.00 1.07 1.09 1.05 1.12 4.25 2.32 6.26
8.5 8.0 7.5 7.0 6.5 6.0 5.5 5.0 4.5 4.0 3.5 3.0 2.5 2.0 1.5 1.0 0.5 0.0
f1 (ppm)

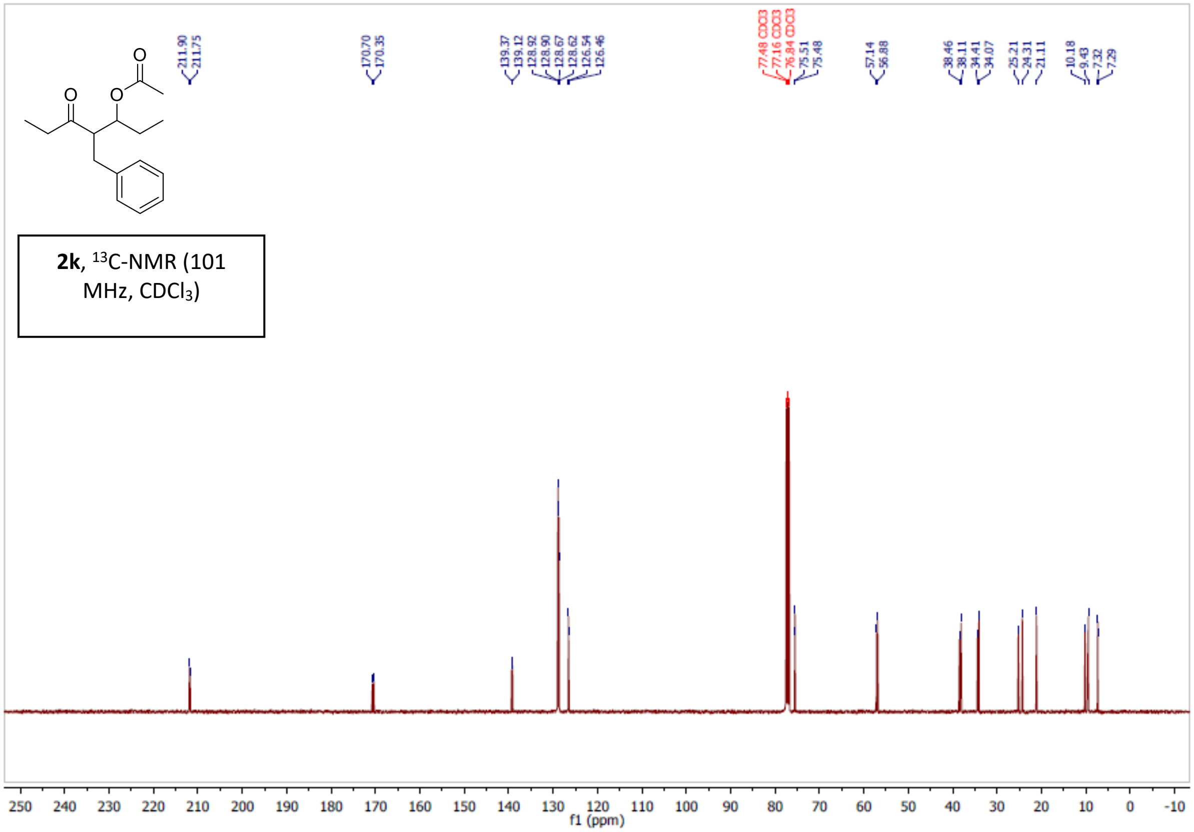
211.90 211.75
170.70 170.35
139.37 139.12 128.92 128.90 128.67 128.62 126.54 126.46
77.48 CDCl3 77.16 CDCl3 76.84 CDCl3 75.51 75.48
57.14 56.88
38.46 38.11 34.41 34.07
25.21 24.31 21.11
10.18 9.43 7.32 7.29
O
O
O
2k, ^{13}C-NMR (101 MHz, $CDCl_3$)
250 240 230 220 210 200 190 180 170 160 150 140 130 120 110 100 90 80 70 60 50 40 30 20 10 0 -10
f1 (ppm)

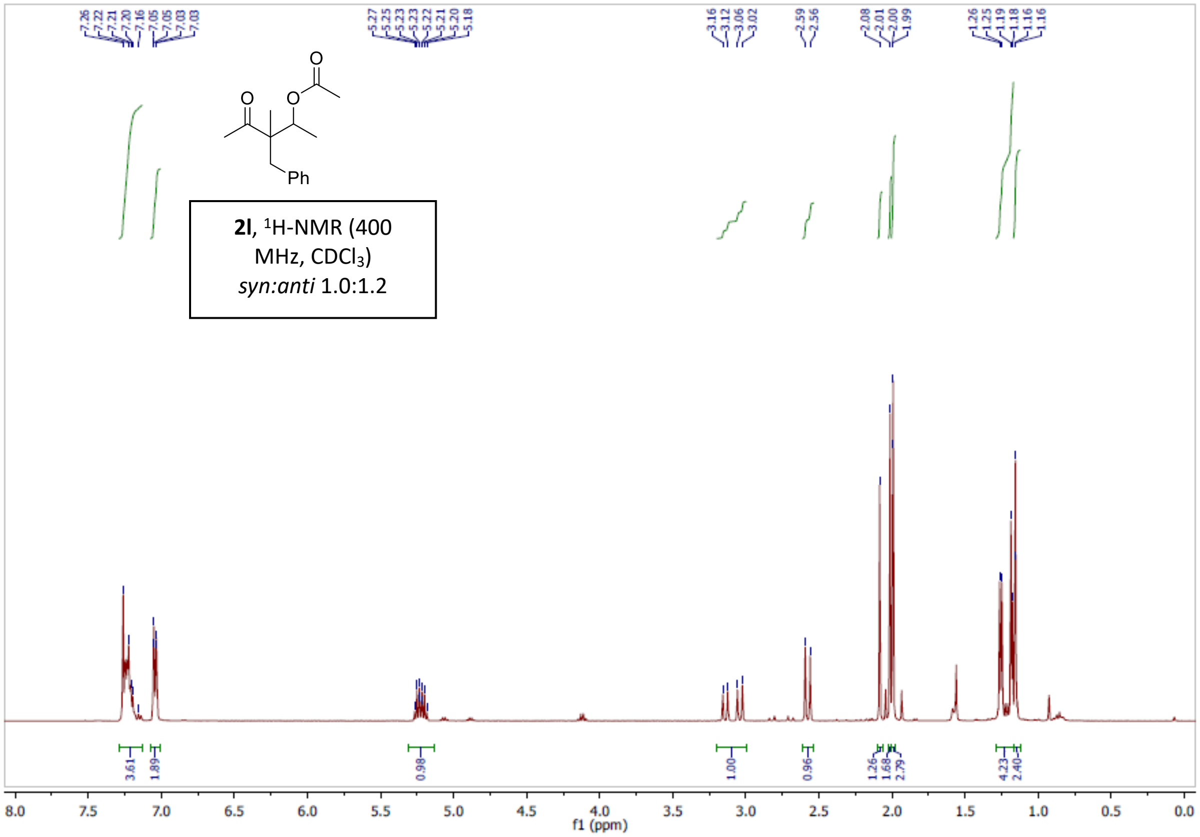

2l, ^{1}H-NMR (400 MHz, $CDCl_3$)
syn:anti 1.0:1.2
Ph
f1 (ppm)

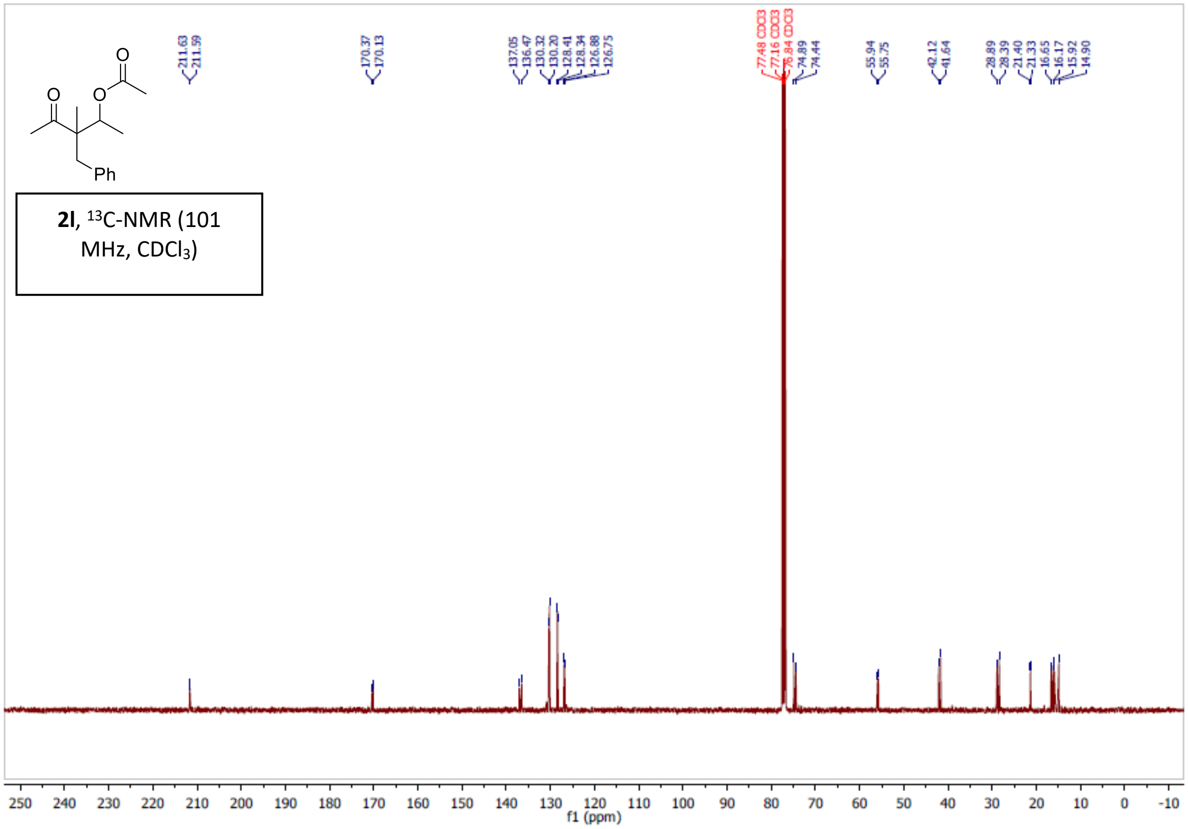

2l, ^{13}C-NMR (101 MHz, $CDCl_3$)
Ph
f1 (ppm)

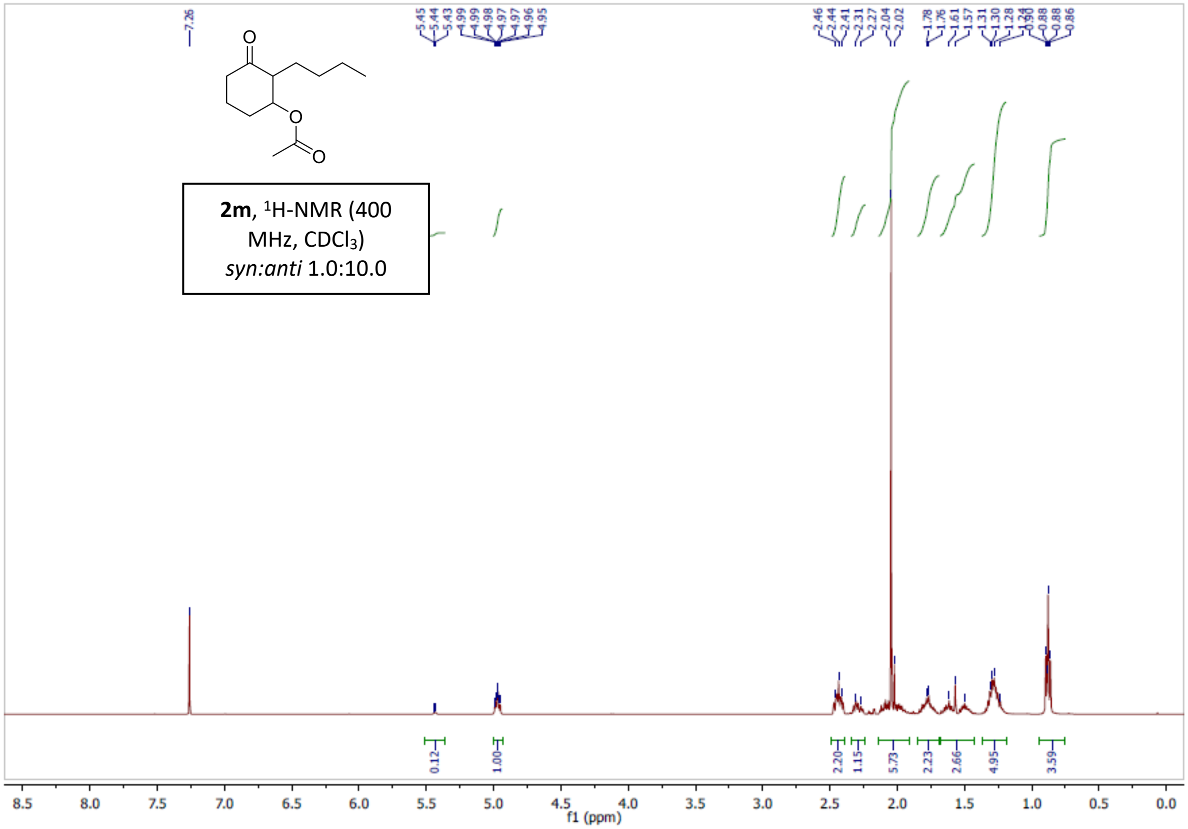

2m, ^{1}H-NMR (400 MHz, $CDCl_3$)
syn:anti 1.0:10.0
f1 (ppm)

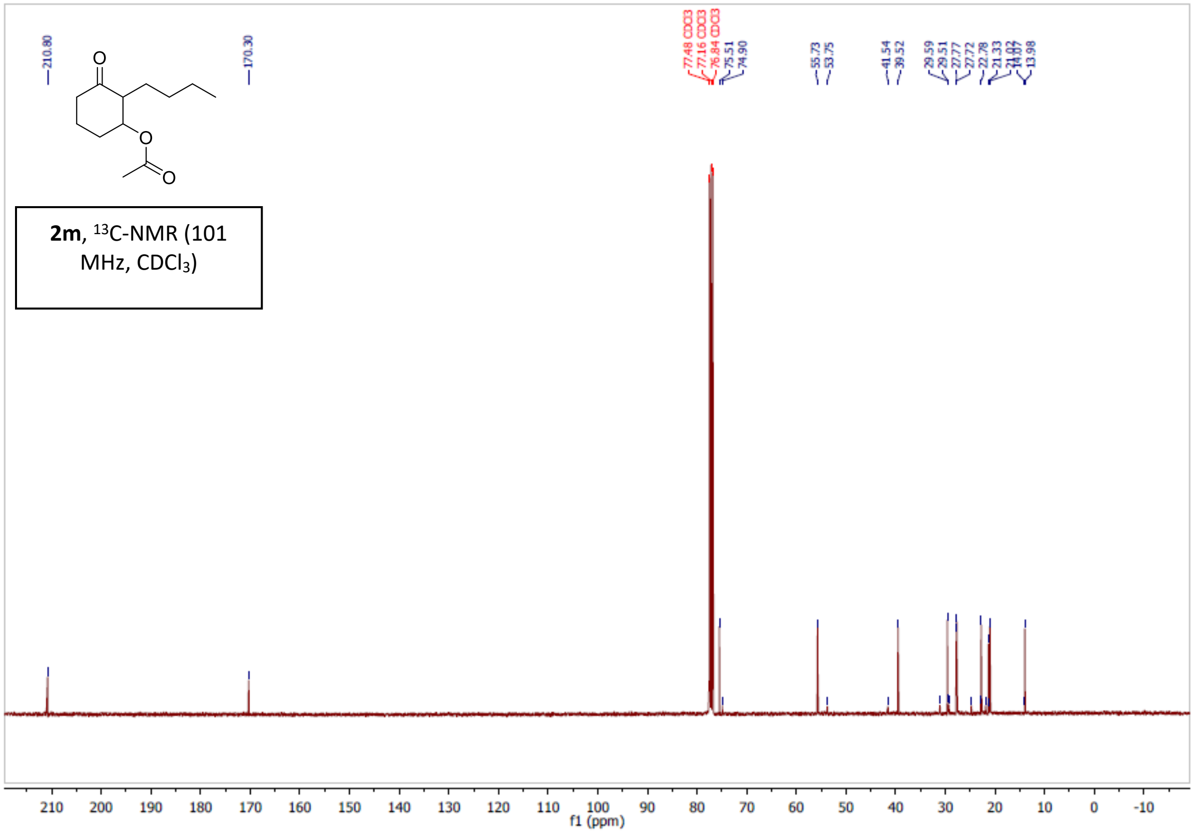

2m, ^{13}C-NMR (101 MHz, $CDCl_3$)
f1 (ppm)

1H-13C HSQC spectra of 1a, 1i and 1k

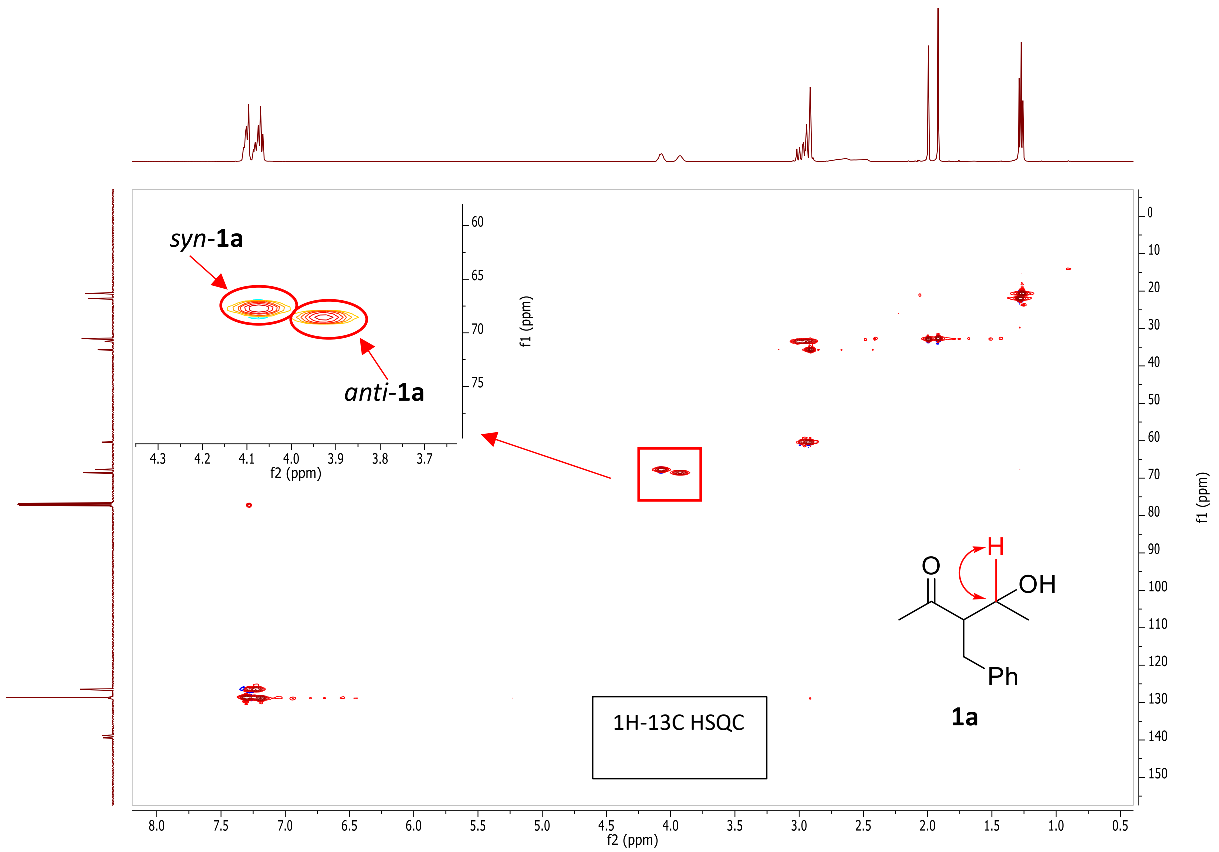

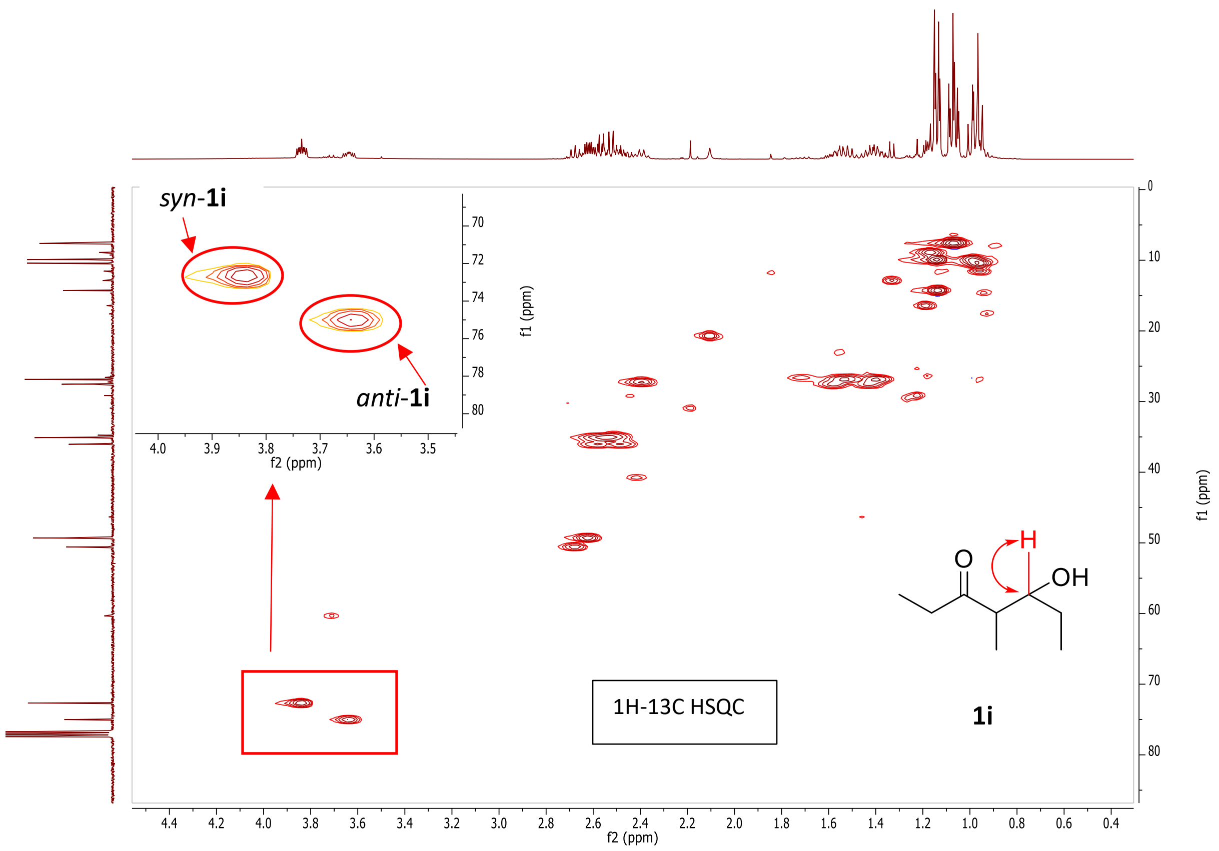

syn-1i
anti-1i
f2 (ppm)
f1 (ppm)
1H-13C HSQC
O
H
OH
1i

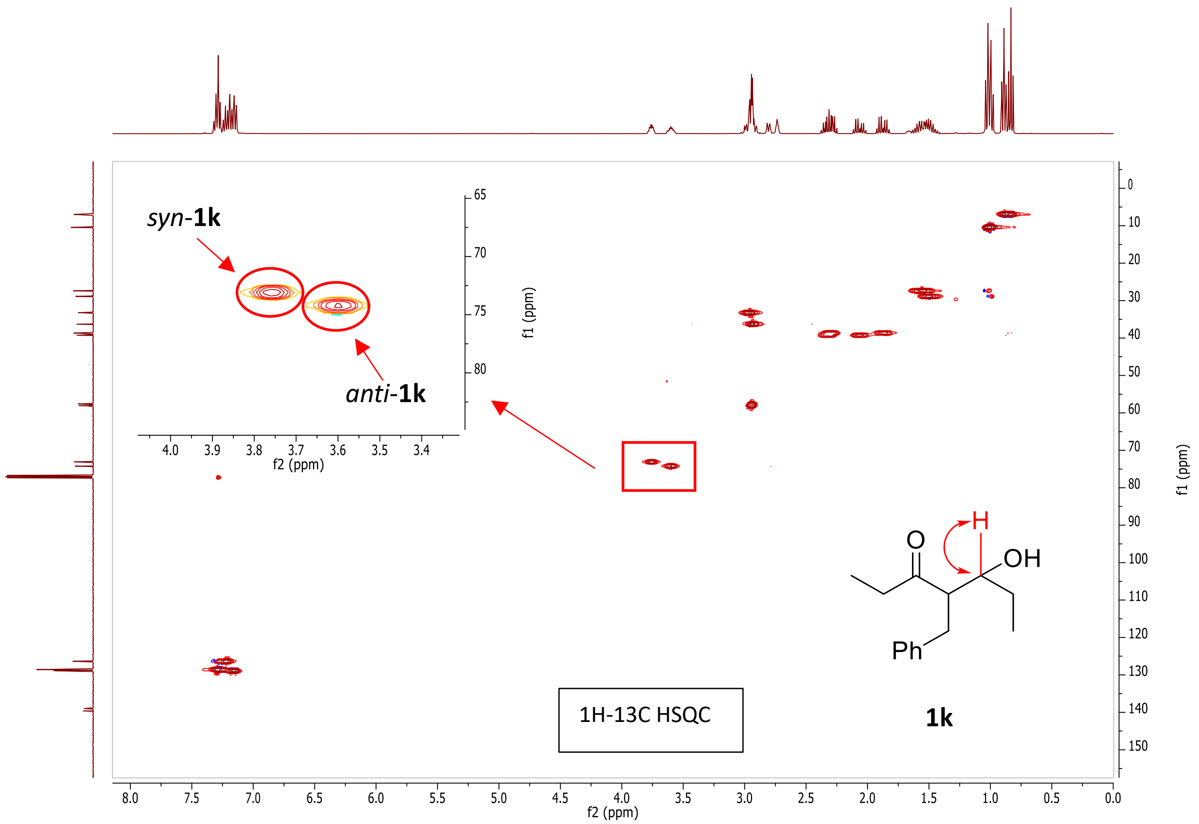

syn-1k
anti-1k
f2 (ppm)
f1 (ppm)
1H-13C HSQC
O
H
OH
Ph
1k
